# Population Genetics of Atlantic Salmon (*Salmo salar*) in Prince Edward Island, Canada

**DOI:** 10.1002/ece3.71285

**Published:** 2025-05-14

**Authors:** Carissa M. Grove, Scott D. Roloson, Kyle M. Knysh, Scott A. Pavey, David K. Cairns, Robert F. Gilmour, Michael R. van den Heuvel

**Affiliations:** ^1^ Canadian Rivers Institute University of Prince Edward Island Charlottetown Prince Edward Island Canada; ^2^ Fisheries and Oceans Canada Charlottetown Prince Edward Island Canada; ^3^ Canadian Rivers Institute University of New Brunswick Saint John New Brunswick Canada; ^4^ Department of Sustainable Design Engineering University of Prince Edward Island Charlottetown Prince Edward Island Canada

**Keywords:** Atlantic salmon, diversity, genetics, population, stocking

## Abstract

Atlantic salmon ( *Salmo salar* ) have experienced population declines across their native range. Widespread stocking has been a population recovery strategy, but there is a growing awareness that stocking may put genetic integrity at risk. In Prince Edward Island, Canada, over 37 million salmon have been stocked since 1880. This study used a panel of six microsatellites and next‐generation sequencing to evaluate the genetic composition of 884 individuals from 20 rivers. Bayesian clustering methods inferred groupings that were generally consistent with the spatial distribution of rivers. A cluster in northeastern PEI was the most distinct, clustering separately across all methods. Distance between rivers accounted for 25.8% of the variations, whereas stocking intensity did not predict genetic variation. The genetic composition of the most heavily stocked river changed over a few years, suggesting that wild free‐ranging fish could outcompete stocked fish. Currently, PEI has multiple genetic stocks that are consistent with the post‐glacial biogeography rather than stocking history. Clarification of these adaptations is required to guide the incorporation of genetics into management strategies for the benefit of Atlantic salmon conservation.

## Graphical abstract

Microsatellites were used to evaluate Atlantic salmon populations on PEI and compare to stocking efforts in the region. Relationships between stocking and genetics were unclear, and groupings were more consistent with the geographical distribution of rivers. Post‐glacial biogeography and subsequent colonization better fit the distribution of multiple genetic groups on PEI.

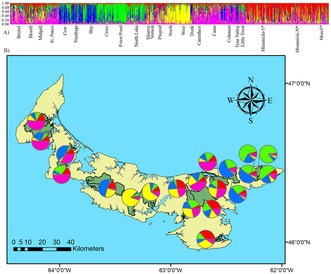

## 1 Introduction

Atlantic salmon ( *Salmo salar* ) populations have declined across their range, including in eastern Canada (Chaput 2012; Pardo et al. 2021). A complete moratorium on commercial fishing of Atlantic salmon in North America, imposed in 2000, has not facilitated substantial population recovery (ICES 2019), and populations in many regions of eastern Canada remain threatened or endangered (COSEWIC 2010). Declines can be attributed to marine mortality of smolts and adults, as well as region‐specific environmental stressors that affect freshwater habitats, which can influence juvenile production (Hvidsten et al. 2015; Forseth et al. 2017; Lennox et al. 2021).

There are a wide range of detrimental anthropogenic modifications of freshwater habitat that can affect juvenile rearing. These can include impediments to fish passage (Nyqvist et al. 2017) and increased sediment deposition (Julien and Bergeron 2006). Interactions with non‐native species, such as Rainbow trout ( *Oncorhynchus mykiss* ) may pose further threats, but interspecific competition is not fully understood (Thibault et al. 2009; Roloson et al. 2018). Regional variables such as acid rain in Nova Scotia (Watt 1986; Kroglund et al. 2008; Gibson et al. 2009) or open‐net pen salmon aquaculture, whereby escapees may interbreed with native stocks (Carr and Whoriskey 2006; Karlsson et al. 2016; Bradbury et al. 2020) may affect wild fish survival and reproductive success. Stocking for population enhancement may have unintended consequences as conspecifics may have stronger effects on resident individuals than competitive pressures associated with interspecific competition (Buoro et al. 2016).

The homing behavior of Atlantic salmon to natal rivers has facilitated the evolution of genetically distinct populations that optimize fitness in local environmental conditions (Garcia de Leaniz et al. 2006; Aykanat et al. 2015; Cauwelier et al. 2018; Wellband et al. 2018). Adaptation occurs over many years, with Atlantic salmon recolonizing North America after the last ice age ~10,000 before present (BP; Bernatchez and Wilson 1998; Rougement and Bernatchez 2018). A small proportion of returning spawners (3%–6%) may stray to other typically close rivers, leading to inter‐river genetic exchange (Keefer and Caudill 2014; Nilsen et al. 2023). This produces a hierarchical genetic structure in Atlantic salmon, with identifiable groupings at scales of continents, regions and rivers (Lehnert et al. 2023). In addition to distance among rivers, Atlantic salmon genotypes also vary with climate and habitat characteristics (Bradbury et al. 2014). The enhancement of salmonids through stocking has historically been undertaken without any regard for local genetic structure and adaptations (Stewart et al. 2002; Ayllon et al. 2006; Ciborowski et al. 2007; Savary et al. 2017). It has been suggested to minimize alterations in native genotypes to optimize smolt production, size and ultimately marine survival (Stewart et al. 2002; Waples and Naish 2009; Hargrove et al. 2021).

Atlantic salmon research in North America has traditionally focused on large (≥ 50 km in length) iconic salmon rivers leaving the contributions of multitudes of small rivers understudied. On Prince Edward Island (PEI), Canada, rivers are short and have ideal temperature conditions for salmonids due to the abundance of groundwater input (Knysh et al. 2016; Roloson et al. 2018). At least 71 PEI rivers are thought to have hosted Atlantic salmon populations in pre‐colonial times (Cairns et al. 2010). However, between 2000 and 2019 Atlantic salmon have been detected in only 40 PEI rivers, of which only 12 have yielded detections in every monitoring year, suggesting that most salmon rivers have precarious populations (Cairns and MacFarlane 2015; Cairns et al. 2023). Atlantic salmon stocking has been intense on PEI, with more than 37 million released since 1880, most of which were early‐stage juveniles (Cairns et al. 2023). Beginning in 1975, a concerted effort was launched to build early Atlantic salmon runs (return prior to September) that would provide summer angling opportunities (Ducharme 1977; Bielak et al. 1991). This was accomplished by stocking the progeny of early‐run broodstock from the Miramichi River (Davidson and Bielak 1993). The effects of mass stocking on the genetic composition of PEI Atlantic salmon populations is unclear. A recent North American study that included five PEI rivers found two distinct genetic groupings were present on PEI; one was grouped with the surrounding Gulf of St. Lawrence population and the other unique to PEI (Moore et al. 2014a). Moore et al. (2014a) hypothesized that the three Gulf‐aligned sites reflected intense stocking of salmon, while the unique northeast PEI sites reflected an ancestral PEI genotype.

The objective of the present study was to further refine the understanding of the genetic composition of Atlantic salmon on PEI populations, in the context of stocking. We hypothesized that there would be a high genetic similarity and greater admixture of salmon populations in rivers that have been heavily stocked, while the putatively native genetic signature, showing genetic differentiation, would occur in infrequently stocked rivers. This hypothesis was examined by sampling juvenile Atlantic salmon in all PEI rivers known to still contain this species (Guignion 2009). Genetic diversity was evaluated using DNA microsatellites and next‐generation sequencing with fin clips obtained from juveniles and smolts. Stocking records were compiled from multiple resources to assess the relationship between stocking and genetic composition of rivers.

## 2 Methods

### 2.1 Study Location

**FIGURE 1:**
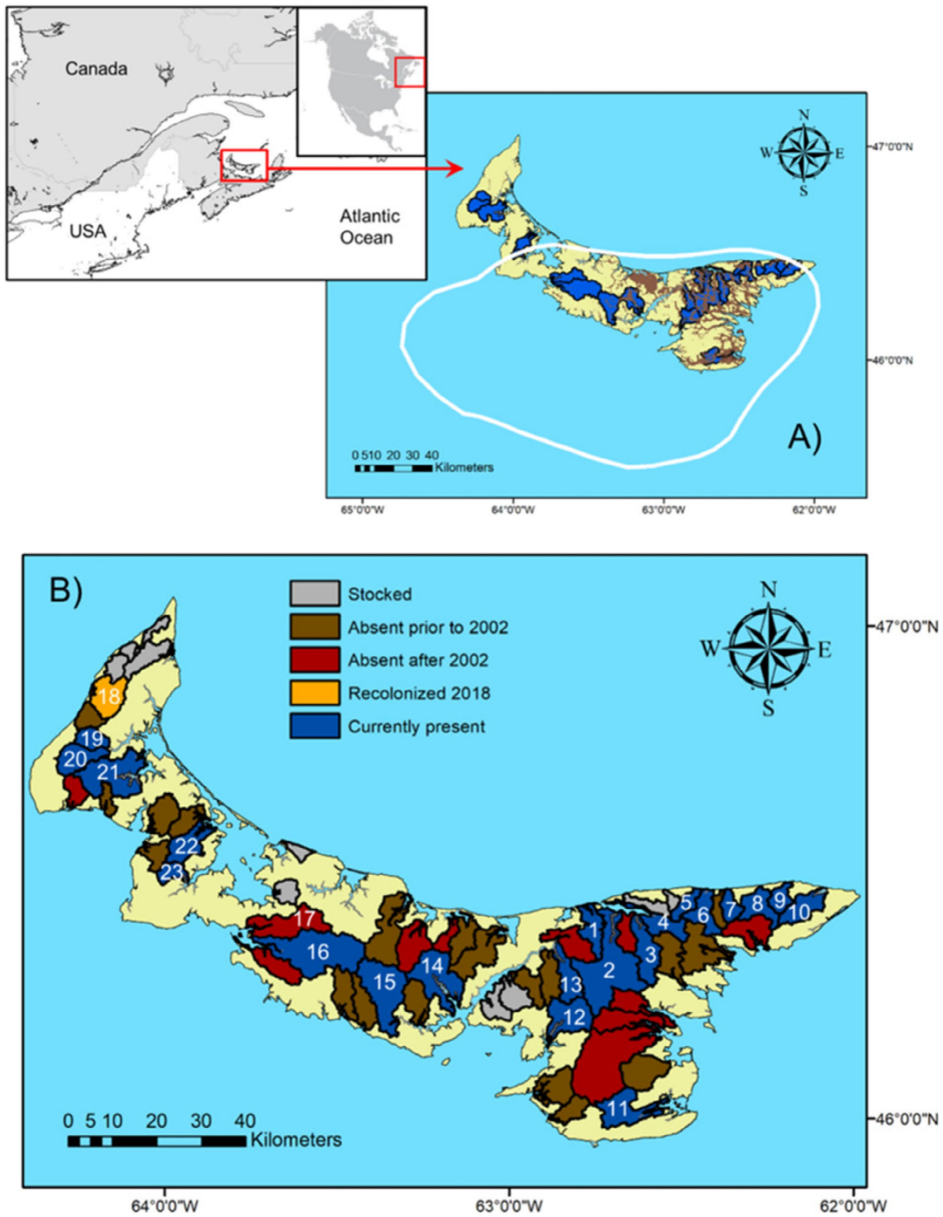
Prince Edward Island, Canada, where (A) blue areas are current watershed boundaries where Atlantic salmon sampling occurred, and brown areas indicate post‐glacial alluvial fans from the PEI surficial geology data (van de Poll 1988). The white line represents the approximate glacial layer at 13,000 years BP (Dalton et al. 2023). (B) Rivers known to contain Atlantic salmon in the past 100 years. Gray watershed boundaries are regions that have been stocked but no records on extirpation; brown boundaries where salmon were extirpated prior to 2002; red boundaries show sites where extirpation occurred after 2002; blue and orange boundaries are watersheds that still have Atlantic salmon present. Blue and orange watersheds are 1—Bristol Creek, 2—Morell River, 3—Midgell River, 4—St. Peters River, 5—Cow River, 6—Naufrage River, 7—Hay River, 8—Cross River, 9—Priest Pond Creek, 10—North Lake Creek, 11—Murray River, 12—Vernon River, 13—Pisquid River, 14—North River, 15—West River, 16—Dunk River, 17—Wilmot River, 18—Miminegash (orange; not included in this study, recolonized after 2018), 19—Cains Brook (Mill River), 20—Carruthers Brook (Mill River), 21—Trout Coleman, 22—Tyne Valley, 23—Little Trout.

PEI is located in the Gulf of St. Lawrence and is Canada's smallest province with an area of 5660 km^2^. Parts of central and eastern PEI were among the last to be deglaciated during the Pleistocene glaciation, approximately 10,000–13,000 years BP (Shaw 2005; Dalton et al. 2020, 2023). Fluvial deposits made post‐glaciation show that this initially resulted in an extensive, interconnected, north and east flowing fluvial network, including glacial lakes that now form salt water embayments (van de Poll 1983; Figure 1a). While this interconnected north and east flowing fluvial network no longer exists, and some of these systems became southward flowing, these fluvial deposits remain the beds of current day streams. Portions of the Laurentide glacier that covered PEI began recession at the westernmost portion of the island, followed by the northeast tip. The last remnant glacial mass was covering the area of interconnected glacio‐fluvial deposits separate from the most northeastern and southeastern rivers (van de Poll 1983; Dalton et al. 2020). Nevertheless, 8000 years BP, PEI shifted to a wide peninsula, with the Northumberland strait area previously draining tributaries from PEI and New Brunswick as an eastern flowing river (Shaw 2005). The modern‐day island was re‐exposed approximately 6000 years ago and its lithology is composed of Carbo‐Permian sandstone that is overlain with glacial till (van de Poll 1983; Shaw 2005). Holocene marine transgression has resulted in shorter river systems on PEI with large estuaries (van de Poll 1988; Shaw 2005). As glaciers receded, Atlantic salmon widely established populations in suitable rivers (Dunfield 1985). Post‐European colonization, as Atlantic salmon populations declined, stocking programs were introduced (Dunfield 1985; Cairns et al. 2010, 2023).

### 2.2 PEI Stocking History

Atlantic salmon stocking records for PEI were derived from published and unpublished records with corrections and additions from previous compilations (Cairns et al. 2010, 2023). Total salmon stocked per river, life stage when stocked, and broodstock origin were compiled from these reports to assess how these have changed over time and how total stocking numbers influence genetic structure. The earliest records of stocking date back to 1880 and stocking still occurs presently. This study focused on rivers from which tissue was collected. Sources and complete records for all PEI can be found in Supporting Information up to 2023 (Table S1).

### 2.3 Genetic Sample Collection

Fish were captured by electrofishing and handled using the approved University of Prince Edward Island Animal User Protocol 16‐044 following Canadian Council on Animal Care (CCAC) standards. Animals were retained briefly for sample collection and released alive in the same location. The entire known distribution of Atlantic salmon on PEI (Guignion 2009) provided the basis for river selection. A total of 22 rivers from Guignion's (2009) list, however, only caught salmon in 21 rivers and achieved the desired sample size of 40 individuals in only 16 rivers. The five other sites ranged in sample size from 7 to 37 individuals (Figure 1, Table 1). Tissue sample collection took place during 2016 and 2017 (Table 1). Atlantic salmon were subsequently discovered in one additional watershed in Western PEI in 2018 (Figure 1b) but samples were not able to be incorporated in the present study. Electrofishing capture efforts (Smith‐root LR‐24, Vancouver, WA, USA) were completed in all 22 rivers in or around riffle and shallow sections. Electrofishing was conducted in reaches of rivers either known to show salmon spawning activity or known to contain salmon from previous electrofishing efforts. Juvenile salmon including young‐of‐the‐year (YOY), parr, and smolts were caught and retained briefly for tissue collection. Capture efforts continued until a minimum nominal sample size of 40 individuals was obtained, where possible. Electrofishing reaches varied in size, and in most rivers, multiple reaches were fished to achieve a full sample size. If the sample size for a river was not reached after 1 day, electrofishing was repeated over multiple days at new sites or, in some cases, over 2 years, 2016 and 2017. Some rivers did not produce the nominal sample size after multi‐day and year electrofishing efforts. Adipose fins were cut with a pair of sterile surgical scissors and placed in a microcentrifuge tube filled with 95% ethanol. Sampling equipment was rinsed with 100% ethanol between individuals and washed with 100% ethanol and 10% bleach between sites. Salmon were released immediately after tissue collection, and samples were stored at room temperature until DNA extraction.

**TABLE 1:** Summary of the area, percent forest cover and percent agriculture within each watershed, and sample size of Atlantic salmon adipose fin clips collected in 2016 and 2017.

River	Area (km^2^)	Forest (%)	Agriculture (%)	2016	2017	Total
1. Bristol	43.4	55.4	27.4	50	0	50
2. Morell	175.5	55.9	28.9	45	0	45
3. Midgell	62.2	65.9	20.6	12	50	62
4. St. Peters	46.2	45.2	36.6	50	0	50
5. Cow	22.9	79.6	7.3	8	44	52
6. Naufrage	43.5	74.0	11.2	0	57	57
7. Hay	24.0	84.2	6.7	49	0	49
8. Cross	45.0	82.4	10.6	51	0	51
9. Priest Pond	24.8	83.8	10.2	0	56	56
10. North Lake Creek	47.5	74.5	14.9	59	0	59
11. Murray	70.1	65.1	19.6	11	0	11
12. Vernon	70.2	42.4	41.5	6	8	14
13. Pisquid	49.3	50.7	34.8	7	33	40
14. North River	97.1	15.5	54.1	27	10	37
15. West River	113.4	51.1	37.7	47	0	47
16. Dunk River	166.6	23.5	67.6	7	0	7
17. Wilmot	82.9	11.0	76.2	0	0	0
18. Mill Carruthers	50.1	56.1	31.9	47	0	47
19. Mill Cains	28.6	68.8	22.5	41	13	54
20. Trout Coleman	108.1	45.2	39.7	47	0	47
21. Tyne Valley	51.5	51.0	34.1	7	3	10
22. Little Trout	23.8	56.0	29.0	0	24	24
Total				540	344	884

### 2.4 DNA Extraction

A modification of the Chelex extraction method was used for DNA extraction (Walsh et al. 1991). A 10% (w/v) Bio‐rad Chelex100 resin (Hercules, CA, USA) slurry was used for extractions with adipose fin clip tissue. Between samples, forceps were sterilized by soaking in 100% ethanol followed by burning. Samples were vortexed for 15 s, followed by centrifugation at 10,000 *g* for 15 s in an IEC microlite RF refrigerated microcentrifuge (Thermo Electron Corporation; Waltham, MA, USA) at room temperature. Samples were incubated for 20 min at 95°C, then removed and vortexed for 15 s, followed by the last centrifugation as previously described.

### 2.5 Library Preparation

Samples were genotyped using next‐generation sequencing of six microsatellite loci. Preparatory steps were as described in Zhan et al. (2017) with modifications. These included multiplexing PCR with all microsatellite primers, indexing PCR to label individual Atlantic salmon, product purification and size selection, quantification, and pooling for Illumina sequencing. A panel of six microsatellites was chosen from Moore et al. (2014a) and forward and reverse microsatellite primers were supplied from IDT (Integrative DNA Technologies; Coralville, IA, USA; Table 2) that were tailed with Illumina sequencing primers. Libraries were completed in multiplex reactions and performed on an Eppendorf Mastercycler EP Gradient Thermal Cycler (Eppendorf, Hamburg, Germany) or Bio‐radT100 Thermal Cycler (Hercules, CA, USA) using the following parameters: 94°C for 15 min, followed by 20 cycles of 94°C for 30 s, 57°C for 180 s, 72°C for 60 s, with a final extension at 68°C for 30 min.

**TABLE 2:** Selected microsatellite loci and primer sequences.

Locus name	Forward primer (5′–3′)	Reverse primer (5′–3′)
SsaD486	TCGCTGTGTATCAGTATTTTGG	ACTCGGATAACACTCACAGGTC
SSsp2210	AAGTATTCATGCACACACATTCACTGC	CAAGACCCTTTTTCCAATGGGATTC
SSsp2215	ACTAGCCAGGTGTCCTGCCGGTC	AGGGTCAGTCAGTCACACCATGCAC
Ssa85	AGGTGGGTCCTCCAAGCTAC	ACCCGCTCCTCACTTAATC
Ssa197	GGGTTGAGTAGGGAGGCTTG	TGGCAGGGATTTGACATAAC
Ssosl417	TTGTTCAGTGTATATGTGTCCCAT	GATCTTCACTGCCACCTTATGACC

Indexing sequences were added to the multiplexed PCR products with a second PCR step. Forty forward indexing oligonucleotides and 25 reverse oligonucleotides (IDT; Coralville, IA, USA) were used in unique combinations to differentiate between 884 individuals submitted for Illumina sequencing. Reactions were performed using the following parameters: 95°C for 2 min, followed by 18 cycles of 95°C for 20 s, 60°C for 60 s, 72°C for 60 s, with a final extension at 72°C for 10 min.

Indexed PCR products were cleaned using Magbio High prep PCR reagent (Gaithersburg, MD, USA) with a ratio of 0.9:1 (beads: DNA) to select for amplicons > 200 bp; anything smaller than 200 bp could not be a product of the chosen microsatellites. This was followed by quantification using the PerfeCTa NGS Library Quantification Kit for Illumina Sequencing Platforms (Quanta Bio, Beverly, MA, USA) with a Bio‐rad CFX Connect Real‐Time PCR Detection System (Hercules, CA, USA). Samples were then pooled and sent to Genome Quebec for Illumina MiSeq PE200 v3 analysis. Following sequencing, the software MEGASAT (Zhan et al. 2017) was used to demultiplex and score microsatellite alleles. Evaluation of histogram outputs was used to confirm and check allele scores, and if 50% or more of the alleles were missing from an individual, it was excluded from any further analyses.

### 2.6 Statistical Analysis

Microsatellite data within populations were analyzed using multiple tests. Mean alleles per locus, allelic richness, Garza‐Williamson index, and observed and expected heterozygosity were estimated using ARLEQUIN version 3.5.2.2 (Excoffier and Lischer 2010). Departures from Hardy–Weinberg equilibrium (HWE) were tested using a locus‐by‐locus test type with 1,000,000 steps in Markov chain and 100,000 dememorization steps using ARLEQUIN 3.5.2.2. Linkage disequilibrium (LD) between loci pairs was tested using GENEPOP version 4.8 (Rousset 2008) set to 1000 iterations per batch and 100 batches. Significance values for HWE and LD were corrected using false discovery rate (FDR) in R version 3.6.3 (R Core Team 2016).

Population structure and differences between populations were evaluated in multiple ways. Pairwise Fst and analysis of molecular variance (AMOVA) were estimated using ARLEQUIN 3.5.2.2. Allele scores were evaluated to look for the presence of private alleles between PEI rivers and genetic groupings; as this would help support differences seen between genetic groupings. Data from Moore et al. (2014b) were used to compare PEI genetic data to other northwestern Atlantic rivers, especially within the Gulf of St. Lawrence. Laboratory variation of the allele scores for microsatellites was adjusted by adding or subtracting base pairs to match Moore et al. (2014b; Table A1). An unrooted neighbor joining tree was constructed using Cavalli‐Sforza chord distances, assessing differences from genetic drift, with the software package Phylip version 3.698 (Felsenstein 1993). Sites from each of Moore et al. (2014b) groupings were included in the tree, as well as all PEI sites from their study. Uneven sampling can influence predictions and was mainly evident when constructing the phylogenetic tree; thus, any site with < 20 individuals was excluded from this analysis. Four sites from this study were excluded from the tree due to low sample size: Murray, Vernon, Dunk, and Tyne Valley. The number of possible genetic groupings in PEI was determined using the Bayesian clustering program STRUCTURE version 2.3.4 (Pritchard et al. 2000). STRUCTURE was run with the admixture model, 50,000 burn‐in and 100,000 MCMC repetitions with a range of *K* values from 1 to 10 repeated 10 times for each *K*. Only three Moore et al. (2014b) sites were included based on their tree groupings: two branches of the Miramichi River and Morell River. The appropriate *K* value was determined using Clumpak (Kopelman et al. 2015) and methods by Evanno et al. (2005). Distance between rivers may account for some of the variation seen between groupings. To test this, Google Earth version 7.3.2 (2022) was used to measure the distance between river mouths using the shortest route by water. Geographical distance between rivers was then correlated with Fst by using a Pearson's correlation in R version 3.6.3.

Genetic groupings and diversity within groups were correlated with total salmon stocked to assess stocking effects on PEI Atlantic salmon genetic structure. Using normal probability plots and the Brown‐Forsythe test, the normality and homogeneity of variance assumptions for stocking, stocking intensity, and genetic data were analyzed. As stocking data were zero‐inflated, they did not conform to the assumptions of parametric statistics. Watersheds were categorized according to which genetic group was dominant based on the mean of all fish to be able to compare these groups to the number of salmon stocked. A Mann–Whitney *U*‐test was used to determine if genetic groupings differed in stocking numbers. For the five‐population model, a Spearman correlation was used to determine the relationship of total stocked fish on the genetic groupings. To assess if stocking influences admixture within populations, another spearman correlation was used to evaluate the relationship of stocking and the proportions of each genetic group within a population. These analyses were performed in Statistica v.13.5 and R version 4.3.2. The critical level of statistical significance was set at *p* < 0.05.

## 3 Results

### 3.1 Atlantic Salmon Stocking in PEI

The stocking database contains records of 37,086,338 Atlantic salmon released to PEI waters between 1880 and 2023 (Table 3, Table S1). Numbers are minima because stocking releases in some years and locations were not recorded. Stocking can be grouped into three time periods where numbers, broodstock origin, and life stages varied (Table S1). In the earliest period considered (1880–1974), salmon were widely stocked in response to reported population declines that had occurred since European colonization. This was followed by the period 1975–1987, where efforts focused primarily on producing strong summer runs to increase angling opportunity, mostly on the Morell River. In the most recent stocking era, 1988–2023, broodstock were collected from the Morell River to supply salmon for stocking programs.

**TABLE 3:** Total Atlantic salmon stocked in study rivers from 1880 to 2023 and last recorded year stocked.

River	Total stocked	Last year stocked
Morell	12,811,263	2023
Dunk River	2,686,253	2002
Midgell	2,219,431	1997
Naufrage	1,136,440	1962
St. Peters	1,077,040	1957
North River	993,500	1964
West River	595,228	2022
Murray	382,000	1931
Trout Coleman	369,348	2002
North Lake Creek	139,620	1964
Tyne Valley	117,000	1927
Wilmot	105,000	1926
Vernon	75,000	1899
Cross	76,120	1937
Mill	60,164	2002
Pisquid	0	N/A
Priest Pond Creek	0	N/A
Little Trout	0	N/A
Bristol	0	N/A
Cow	0	N/A
Hay	0	N/A
Total	22,498,435	

**FIGURE 2:**
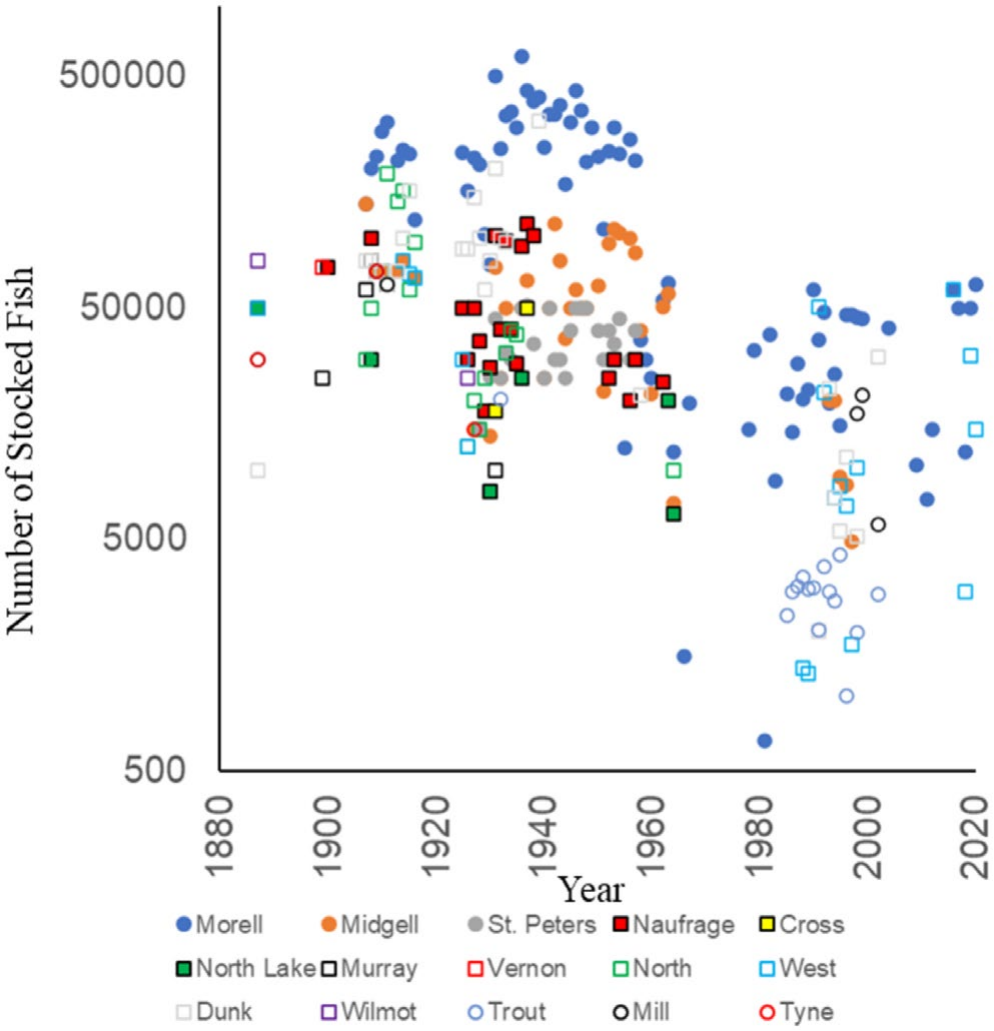
Atlantic salmon stocking history in Prince Edward Island, Canada from 1880 to 2020 showing the number of rivers stocked over time for each.

Between 1880 and 1974, Atlantic salmon were released as YOY (fry, fingerlings and 0+ parr). Broodstock were sourced first from the Dunk River, PEI. Other rivers used as sources of broodstock were River Phillip (Nova Scotia), as well as Winter River and ‘other PEI locations’. From 1899 to 1916 the only sources mentioned were the Saint John River, New Brunswick (3 years) and the Miramichi River, New Brunswick (14 years). Between 1917 and 1974, stocking sources were not documented or of speculative origin. In this first stocking era, salmon were stocked widely, into a total of 54 rivers, including 14 rivers that were sampled in this study (Figure 2, Table S1). This was the only timeframe in which salmon were stocked in the northeastern salmon rivers of the province. Stocked rivers in the area included the Naufrage River which was stocked annually from 1925 to 1938 with a total of 1.1 million salmon. Cross Creek received 76,120 salmon in three stocking years between 1931 and 1937 and North Lake Creek received 139,620 in 4 years between 1930 and 1964. Releases in the Northeast area were < 4% of the 34,988,739 salmon that were stocked between 1880 and 1974. In total, 94% of the salmon stocked in PEI were stocked during this early period.

In response to continual salmon declines, the period between 1975 and 1987 focused on restoring salmon on the Morell River, which had historically been the most important salmon angling river. In addition to the widespread failure of previous stocking programs, the impetus for the shift was falling catch reports on the Morell River; in the 1970's as few as four salmon per year were captured (Bielak et al. 1991; Davidson and Bielak 1992). Broodstock were sourced from early Atlantic salmon runs in the Miramichi River, with additional fish from the Restigouche River, both in New Brunswick. This period accounted for < 0.5% of the salmon stocked on PEI and most (167,446 of 177,935, 95.2%) of the fish in this period were released in the Morell River, with the remainder (8489, 4.8%) in the Mill River (Table S1).

By the late 1980s, annual Atlantic salmon returns to the Morell River had increased to > 1000 fish, with a high early‐run component (Davidson and Angus 1994). In the 1988–2023 period, broodstock were exclusively sourced from the Morell River. In total, 1.1 million salmon were stocked between 1988 and 2003 into Mill River (2.8%), Trout River Coleman (4%), Morell River (39%), Midgell River (5.6%), Valleyfield River (3.2%), West River (9.9%), and Dunk River (7.5%) (Table 1, Figure 2, Table S1). After 2004, 663,800 salmon were stocked into the Morell River (74%) and West River (26%). The 1988–2023 period accounts for 5.1% of all hatchery salmon released. A total of six rivers in this study were never documented to have received any stocked Atlantic salmon: Priest Pond Creek, Hay River, Cow River, Little Trout, Bristol Creek and Pisquid River.

### 3.2 DNA Microsatellite Alleles for PEI

The panel of six microsatellites was applied to 21 Atlantic salmon populations, providing results for a total of 752 individuals once those with 50% or more missing data were removed. The number of alleles per locus ranged from 7 (SsaD486) to 16 (SsosI417) and mean allelic richness ranged from 3.7 to 9.3 among populations. Mean expected and observed heterozygosity were 0.69 and 0.67, respectively. The number of significant comparisons for HWE after correction for FDR decreased from 42 to 31 out of 126 comparisons. Furthermore, only four rivers had no significant HWE comparisons: Morell, Midgell, St. Peters, and North Lake Creek. Only 30 tests out of 315 comparisons of LD remained significant after FDR correction. No pair of loci was found to be consistently in LD. Nine of the 21 rivers had one or more locus pairs in significant LD.

### 3.3 Population Structure

**FIGURE 3:**
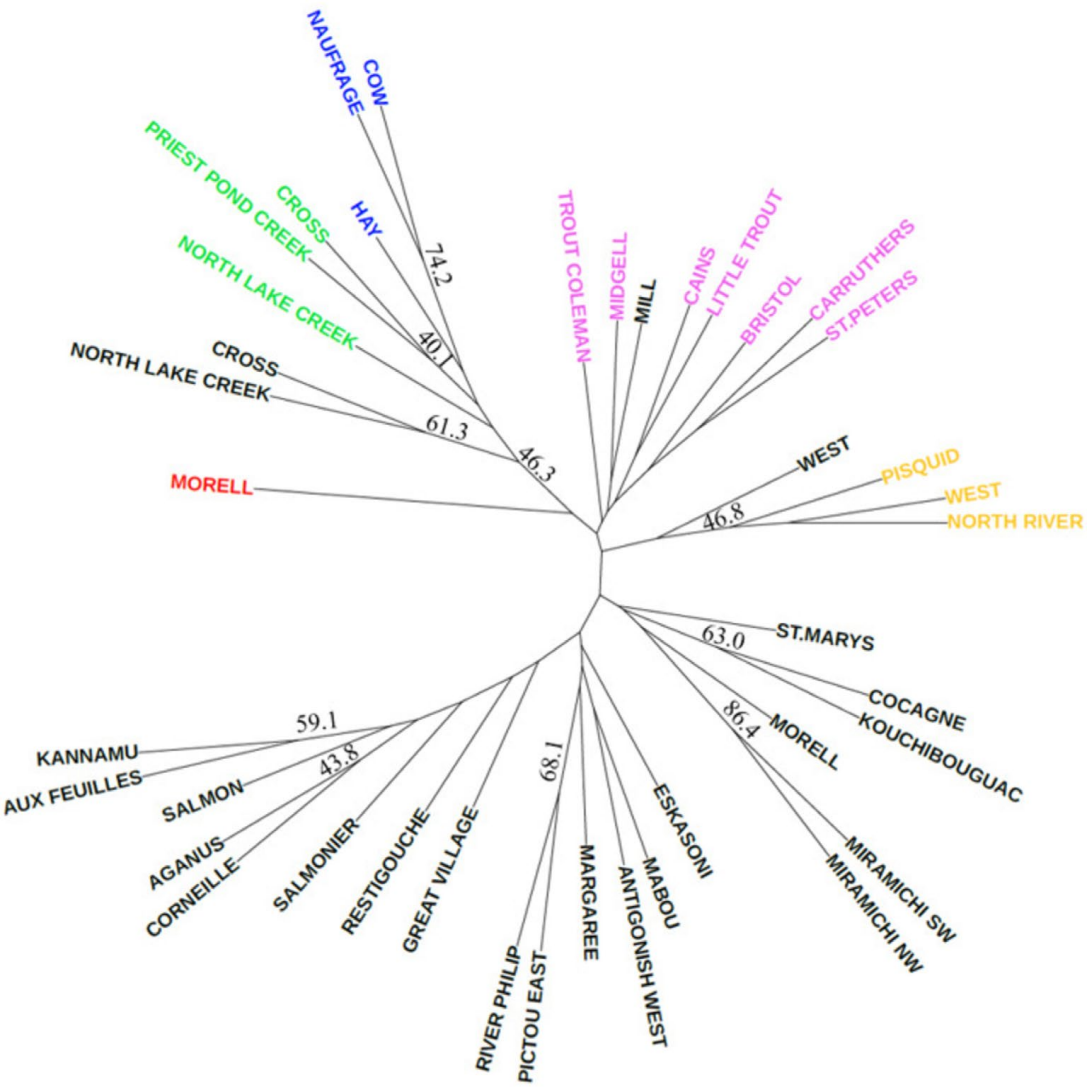
Unrooted neighbor‐joining tree of Cavalli‐Sforza chord distances of Atlantic salmon populations using the software Phylip. Bootstrap values (1000 replicates) are shown. Rivers presented in colors other than black are sites where samples were collected during this study to allow comparisons to previous work (sites indicated in black). Populations of salmon in the rivers having the same color are part of the same haplotyped in the *K* = 5 STRUCTURE model.

**FIGURE A1:**
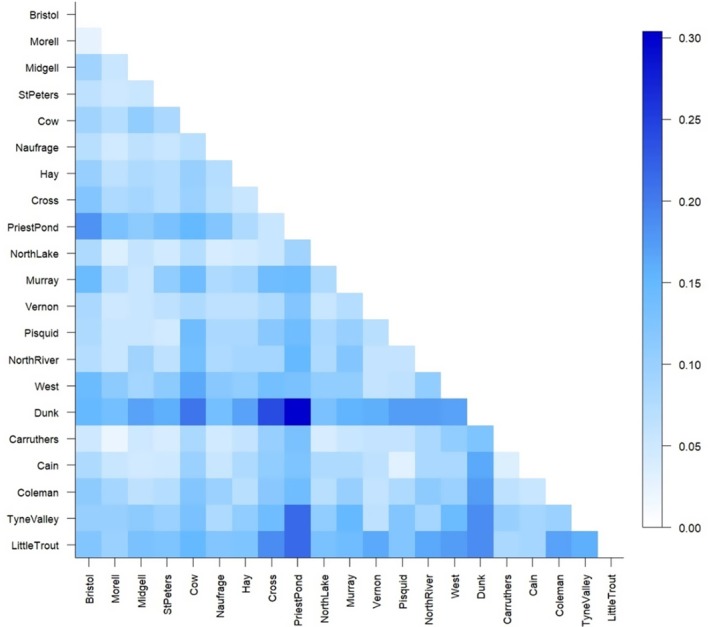
Heat map of pairwise *F*_ST_ values for 21 PEI Atlantic salmon populations. Darker cells indicate larger *F*_ST_ estimates thus greater genetic divergence, whereas lighter or white cells indicate lower estimates.

The average pairwise *F*_st_ among PEI rivers was 0.092 with values ranging from 0.025 to 0.22; all comparisons between population pairs were statistically significant (Figure A1). Locus‐by‐locus variance among and within PEI populations was 9.2% and 90.8%, respectively (AMOVA). Five rivers in Northeastern PEI had an allele (144) at the SSsp2210 locus that was not present in any other PEI rivers. The unrooted neighbor‐joining tree contains both PEI sites from this study and numerous Moore et al. (2014b) sites. It shows several clusters that are consistent with geographical distribution (Figure 3). In general, PEI sites cluster based on region or drainage area consistent with three larger groupings. The northeastern cluster includes Cow River moving east to North Lake Creek. All the southern draining rivers, West River, North River and Pisquid, cluster together and share an estuary complex. The remaining cluster consists of northern draining PEI rivers excluding the aforementioned northeastern cluster. The Morell River is the only site from this PEI dataset that does not group regionally or with any other PEI rivers. When the results from the present study were compared to the results Moore et al. (2014b), all rivers grouped consistently over time excluding the Morell River. The Morell, which grouped with the Miramichi River in the past study, now occupies a separate node on the tree (Figure 3), suggesting a change in the genetic composition of the Morell River Atlantic salmon captured in 2016 as compared to 2010.

**FIGURE 4:**
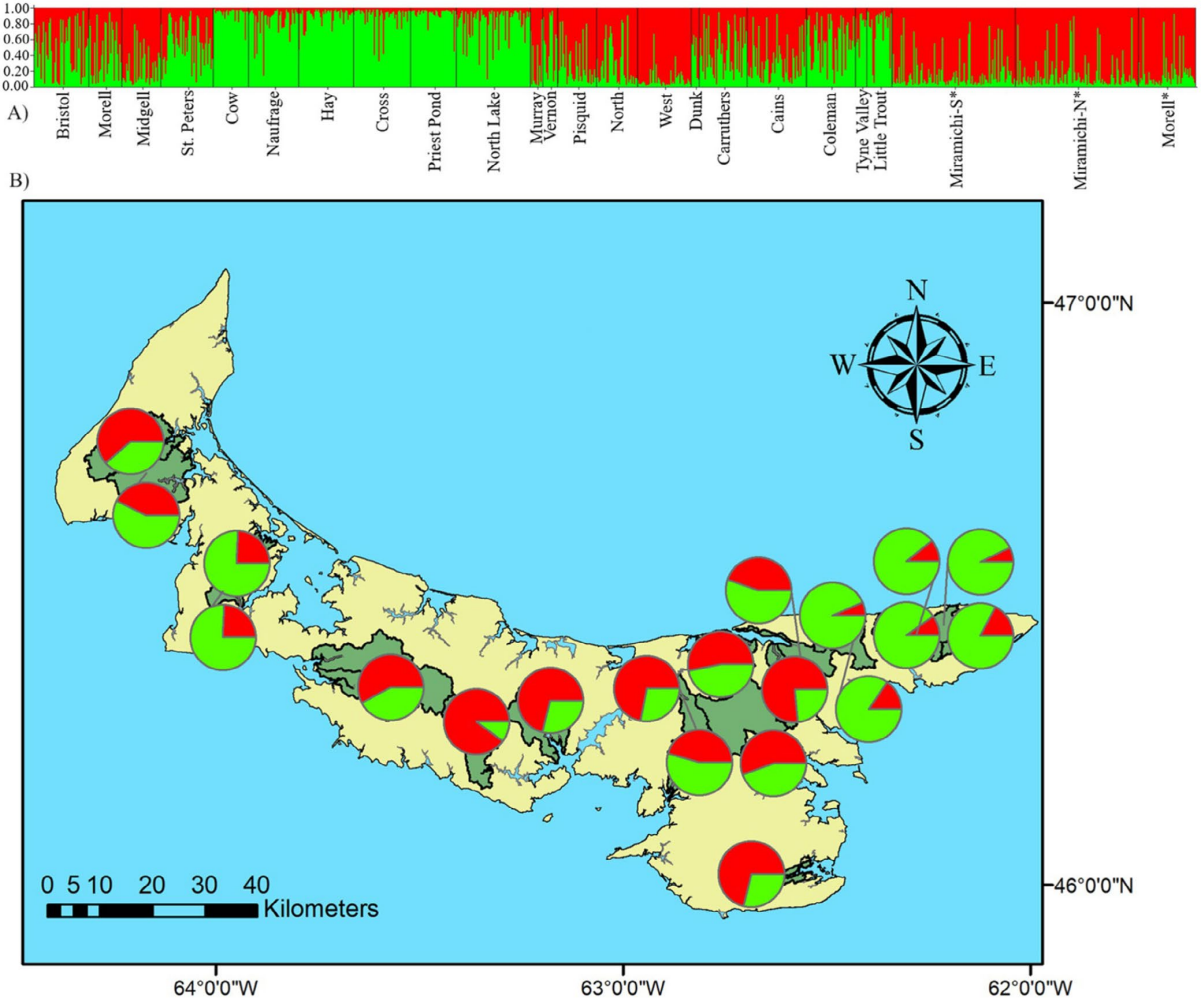
Results of STRUCTURE analysis for two genetic clusters (*K* = 2, Δ*K* = 109.9) of PEI Atlantic salmon (A) Individual membership coefficients within each watershed, and (B) Mean membership coefficient summarized by watershed. *Sites included from Moore et al. (2014a).

**FIGURE A2:**
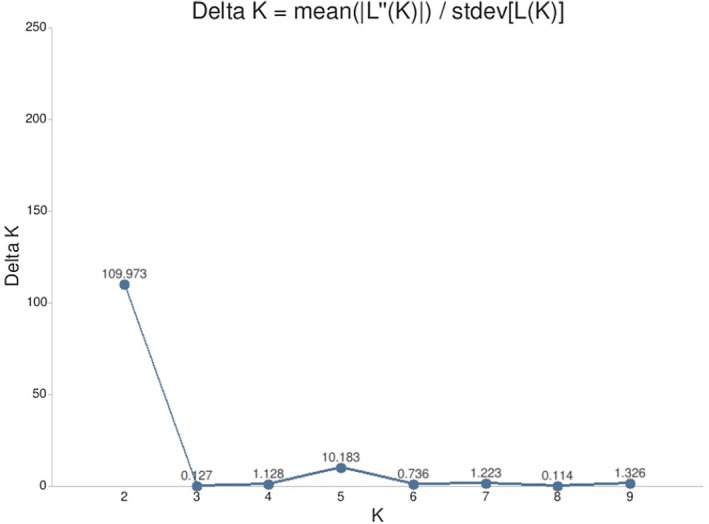
Delta *K* to determine appropriate *K* values from STRUCTURE data.

Evaluation of the change of *K* from the STRUCTURE analysis suggested two values of *K* corresponding to two Δ*K* peaks (number of potential groupings; Figure A2). The *K* = 2 model has two groups referred to as Group 1 (predominant red) and Group 2 (predominant green). Rivers in Group 1 are in highest densities in central PEI and include both north‐and south‐draining rivers, whereas Group 2 is split in eastern and western PEI and is mostly north‐draining except for Vernon River. Four sites had a composition of > 80% to genetic Group 1, including West River and the Moore et al. (2014b) Miramichi and Morell River. All other sites in Group 1 had a composition of 53% to 76%. The northeastern cluster had a composition of > 80% to Group 2 (Figure 4). Other rivers with high proportions of this grouping include Vernon, Coleman, Tyne Valley, and Little Trout Rivers, with values ranging from 55% to 76%.

**FIGURE 5:**
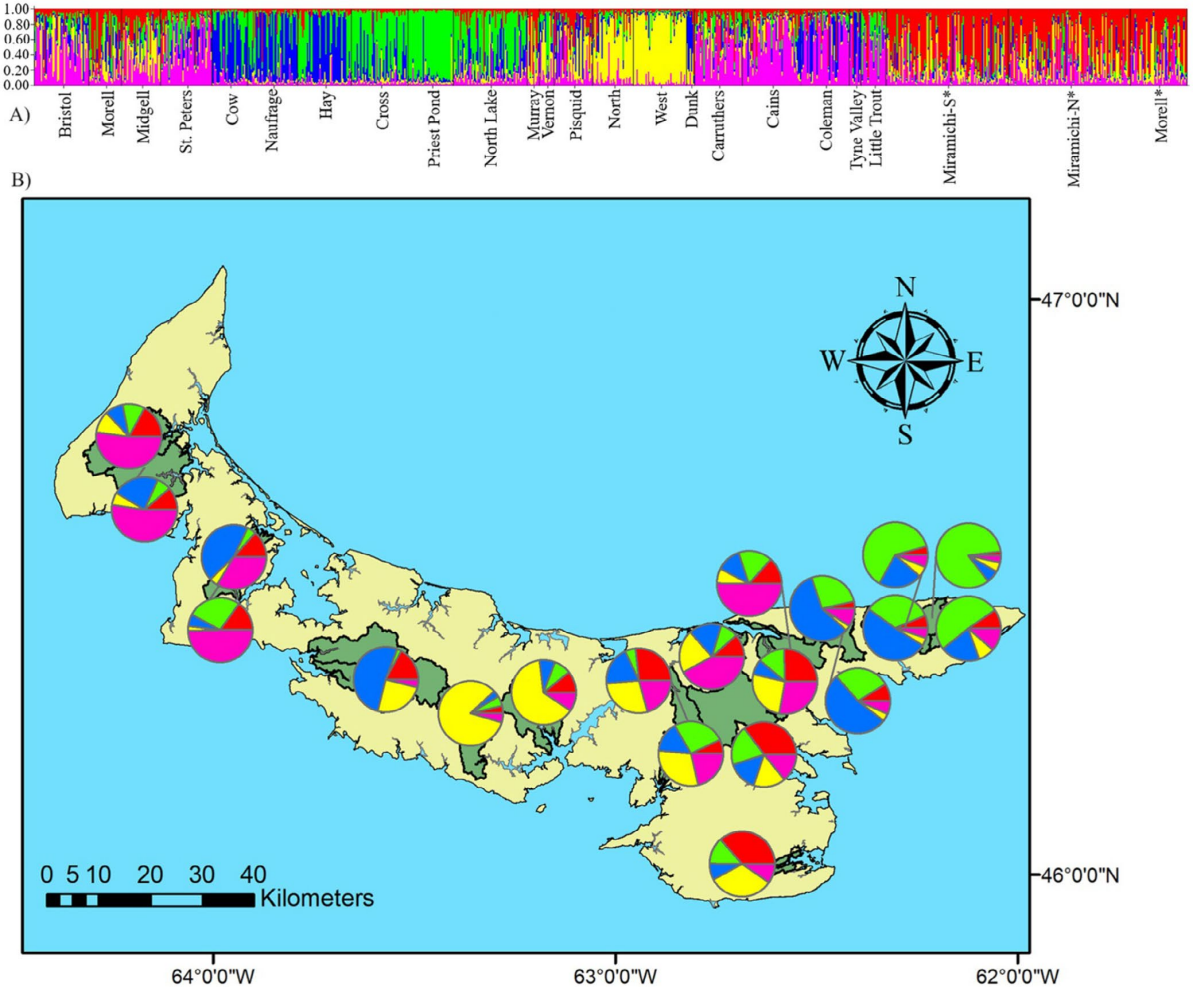
Results of STRUCTURE analysis for five genetic clusters (*K* = 5, Δ*K* = 10.2) from PEI Atlantic salmon: (A) Individual membership coefficients within each watershed, and (B) Mean membership coefficient summarized by watershed. *Sites included from Moore et al. (2014a).

The *K* = 5 model is divided into groups referred to as Northeastern 1 (predominantly green), Northeastern 2 (predominant blue), Northern (predominant pink), Southern (predominant yellow), and Morell (predominant red; Figure 5). This substructure is similar to the clusters determined by the tree and is largely consistent with the regional groupings (Figure 5). The northeastern cluster is further subdivided into two groups in this model, with each having higher proportions of either Northeastern 1 or 2 group. Cross River, Priest Pond, and North Lake form Northeastern 1, whereas Cow, Naufrage, and Hay compose the Northeastern 2 group. This subdivision is also evident on the tree. The other northern draining rivers identified from the tree grouping share similarities in this model as well and make up the Northern group. The North, Pisquid, and West rivers make up the Southern grouping. The Morell River is the only PEI river in the Morell grouping. The rivers sampled in 2010 included from Moore et al. (2014b) the Miramichi River branches and Morell River, which group together. In the present study, the Morell River, sampled in 2016, was less similar to the Miramichi and had more contribution from both northeastern PEI groupings.

### 3.4 Influence of Stocking and Geographical Distance on Genetic Structure

Coastal distance between stream mouth and Fst was weakly, but significantly correlated (*r* = 0.26, *p* = 0.0002), suggesting a role of isolation by distance in structuring rivers. However, the distance between watersheds and stocking is not independent variables since stocking was not conducted in a geographically random manner. To determine if stocked numbers and intensity were correlated with genetic structure, watersheds were initially categorized according to the two‐population structure model based on the majority of fish (over 50%) with total salmon stocked as the independent variable. For the purposes of this analysis, Cains and Carruthers Brook genetics data were collapsed into one population, Mill River. Group 2, including the northeastern shore watersheds from North Lake Creek to St. Peters (*n* = 7) and the Tyne, Vernon, Trout Coleman, and Little Trout rivers had a mean (SEM, *n*) of 271,869.81 (128,610, 11) salmon stocked. Group 1, inclusive of the Morell River and two adjacent watersheds, Mill River, and the remaining locations on the south shore east of the Dunk River had a mean (SEM, *n*) of 2,153,647 (617,017, 9) stocked salmon. Despite an order of magnitude difference in stocking numbers, there was no significant difference in total salmon stocked between the two genetic groups from the *K* = 2 STRUCTURE model using the Mann–Whitney *U*‐test, as there were at least two watersheds in both populations that had no record of stocking. There were no significant correlations between the number stocked and the proportion of each genetic group within a watershed. A similar analysis was conducted with total salmon stocked, and the *K* = 5 STRUCTURE model. There were no significant correlations between numbers stocked and the genetic groupings or the proportion of each genetic group within a watershed using either Spearman correlations.

## 4 Discussion

This study of all known Atlantic salmon rivers on PEI determined that populations from the most northeastern region possess genotypes that are not predominant in other regions of PEI. Our compilation of Atlantic salmon stocking records on PEI showed that a small number of watersheds have received a high proportion of the stocked Atlantic salmon over the past 140 years, with five of the surveyed watersheds accounting for 87% of the stocking in the rivers assessed in this study. Stocking efforts, however, have diminished substantially since the 1970s. Our analysis found that there was no relationship between stocking numbers and Atlantic salmon population structure within watersheds.

The present study confirms there are multiple Atlantic salmon genetic groups in PEI. Two genetic groups (Northeastern 1 and 2) were identified in northeastern PEI that are dissimilar to the rest of PEI. These results support those of Moore et al. (2014a) who examined Atlantic salmon genetics over the entire North American range. Among the 12 genetic groupings identified in Moore et al. (2014a) the study identified Atlantic salmon from northeastern PEI as a sub‐group of the broader Gulf of St. Lawrence genetic group. The present study examined PEI salmon populations on a larger scale and confirmed a unique northeastern PEI genetic group, which was further divided into two subgroups not identified previously. It was determined that this first subgroup was geographically confined to the most northeastern shore of PEI, which includes the two previously studied rivers by Moore et al. (2014a), as well as Priest Pond Creek; whereas Cow, Naufrage, and Hay make up the other northeast subgroup.

One possibility for the different genetic grouping in northeastern PEI is that this represents an ancestral strain as speculated by Moore et al. (2014a), and that the ancestral strain has been largely reduced or eliminated by widespread Atlantic salmon stocking in other rivers on PEI. Stocking can increase admixture and decrease divergence within populations, disrupting the natural structure of these systems (Valiquette et al. 2014; Savary et al. 2017; Wellband et al. 2018). It is difficult to interpret the impacts of historical stocking on contemporary genetic structure. First, the historical records are undoubtedly incomplete with many undocumented stocking efforts and unreported brood stock origins (Cairns et al. 2010, 2023). Hatchery‐reared Atlantic salmon have also been shown to have higher straying rates (3%–15%) than their free‐ranging counterparts (3%–6%; Keefer and Caudill 2014; Nilsen et al. 2023). For example, Pisquid, which is noted as not being stocked, is in close proximity (~5 km) to stocked rivers (having received > 500,000 fish; Cairns et al. 2010, 2023) and may have been influenced by strays from these stocking efforts given similarities in its genetic composition to the region. Alternatively, it is also possible that Pisquid was stocked but never documented. There are also poor records of the contemporary extirpation of salmon populations in rivers, such as from frequent fish kills encountered on PEI (Roloson et al. 2022) or the extensive deforestation and habitat degradation that occurred post‐European colonization (PEI Department of Environment, Energy, and Climate Action 2004). The extirpation of these populations makes it challenging to tease out all the regional differences as there are large geographic areas with no Atlantic salmon left on PEI.

The Morell River population suggests that the genetic composition of stocked populations can change to those of assumed wild populations, leading to the lack of association between genetic structure and intensity of stocking on PEI. While the genetic structure of other streams on PEI tested in both the present study and that of Moore et al. (2014a) were similar, the genetic composition of the Morell River population changed to reflect additional contribution from Northeastern 1 and 2 genetic groups and less Miramichi genetic influence. This shift occurred during a period of high returns based on local redd counts. The number of redds in the northeastern region (Naufrage to North Lake Creek) rose 3.7‐fold between 2010 and 2013 and remained high until 2017 (Souris Wildlife, unpublished data). There was strong evidence of significant straying from natal streams during this period as three streams in the region that were absent of salmon in 2010 (Bear, Hay and Cow Rivers) had established new populations just prior to the present study (Souris Wildlife, unpublished data). Atlantic salmon are known to stray to non‐natal rivers during spawning as one of their life history strategies (Keefer and Caudill 2014; Ulvan et al. 2018). Straying rates have been shown to vary based on population and can increase during periods of high returns, where in some instances they have been shown to stray to rivers > 100 km away (Ulvan et al. 2018). Thus, it is likely that a combination of wild salmon straying to the Morell River, the more recent Morell River broodstock being derived from that river, and the historically poor success of early run Atlantic salmon on PEI caused a rapid shift away from the past genetic composition. Shift towards native genetics is supported by the results of Bootsma et al. (2021) that native genetic structure may be able to ‘purge’ non‐native genetics originating from stocking.

Observed genetic differences appear related to the biogeographical history of PEI, whereby genetic structure could reflect multiple invasion events during glacial recessions between 6000 and 13,000 years BP. North American and European populations of Atlantic salmon are believed to have diverged over 600,000 years BP (Rougement and Bernatchez 2018). However, the second and most recent colonization of North America occurred approximately 10,000 BP after the Laurentian glaciers receded (Bernatchez and Wilson 1998; Rougement and Bernatchez 2018). Lehnert et al. (2019) showed Atlantic salmon recolonization after the Pleistocene glaciers (~10,000 BP) likely occurred from multiple glacial refugia, containing both European and North American genotypes, leading to more complex variations and establishments of salmon populations. Regions of PEI were deglaciated at different times, potentially allowing multiple colonization events of Atlantic salmon. Western PEI was free of glaciers before the rest of the island. As glaciers continued to recede, the northeastern shore was exposed before central and remaining eastern PEI (Figure 1; Shaw 2005; Shaw et al. 2006; Dalton et al. 2020, 2023). Surficial deposits provide evidence for a large fluvial network extending from central to eastern PEI. From Cross River moving east to North Lake Creek, it indicates that these rivers may not have been as interconnected with the larger fluvial network that was present in the majority of eastern PEI (van de Poll 1988). Atlantic salmon are the only known host for the parasitic glochidial stages of the eastern pearlshell mussel ( *Margaritifera margaritifera* ) that must have migrated to PEI attached to Atlantic salmon. While the mussel occurs in all of the streams around St. Peters Bay, with evidence of it occurring in the Northeastern 2 group, it is absent from the Northeastern 1 rivers, further suggesting these populations could have resulted from independent colonization events (Gallant 2022).

Life history traits are known to vary among populations due to the local environment and may have contributed to PEI population structure. PEI streams are presently extensively groundwater‐fed, and short (≤ 45 km) in length (Knysh et al. 2016; Roloson et al. 2018). Anecdotally, PEI Atlantic salmon are often described as ‘late run’ salmon (Cairns and MacFarlane 2015) and the hydrological conditions and lack of refugia in small streams do not favor salmon entering the river until immediately before spawning (Reed et al. 2017). Cairns and MacFarlane (2015) note that run timing and size at the time of river entry have changed in larger PEI rivers (i.e., Morell River) that have been heavily stocked, now being represented by ‘early‐run’ and smaller salmon. Atlantic salmon in North Lake Creek are known to exhibit late run timing (authors, unpublished data). Similar timing differences associated with genetic differences are seen elsewhere, as is the case for the Miramichi River in New Brunswick, Canada, the longest river in the region, where there are genetic differences between upper and lower river Atlantic salmon, but not between two adjacent branches (Wellband et al. 2018). Salmon in the higher reaches start migration upriver earlier in the year (June–July) than lower‐reach fish (Chaput et al. 2016; Wellband et al. 2018). In Norway, the large Teno River also has two subpopulations with low differentiation but with considerable phenotypic differences such as life history strategies, age at maturity, and juvenile growth (Aykanat et al. 2015). It has been demonstrated multiples times that changes in single genes can have large impacts on Atlantic salmon phenotypes (Ayllon et al. 2015; Barson et al. 2015; Cauwelier et al. 2018).

Both Northeastern genetic groupings correspond to the most stable salmon populations on PEI (Guignion 2009; Cairns and MacFarlane 2015; Guignion et al. 2019). In a time when salmon are declining in much of their native range (COSEWIC 2010), Atlantic salmon populations in northeastern PEI have been self‐sustaining. While genetics may be one factor associated with population success, Atlantic salmon presence in PEI rivers has been associated with higher forest cover at the landscape scale and the presence of suitably sized substrate on a reach scale (Roloson et al. 2018). The watersheds of northeastern PEI remain 70%–80% forested and are among the highest‐quality salmon habitat still present on PEI (PEI Department of Environment, Energy, and Climate Action 2010). An abundance of quality habitat may have allowed populations to remain abundant enough to be robust to any intrusions from stocking, a phenomenon which has been observed in other fish species (Bootsma et al. 2021). As northeastern PEI rivers were stocked in the early to middle part of the last century, particularly the Naufrage River (last stocked in 1962), there is the possibility that the reestablishment or maintenance of native genetic composition through natural selection, successful returns and juvenile recruitment of native genetic variants has also occurred over multiple generations on PEI.

Stocking programs, such as those illustrated herein, have historically been conducted with no regard for maintaining local genetic diversity. While habitat is critical, it is likely that the historical disregard for genetic factors has contributed to the failure of stocking programs to re‐establish wild, self‐sustaining populations (Ayllon et al. 2006; Ciborowski et al. 2007). Supportive stocking with closely related stocks may allow for populations to regain some genetic variation similar to the original population (Savary et al. 2017). Recent advances in fisheries science have started to relate genotypic changes to phenotypes with the potential to understand fitness under varying environmental regimes (e.g., Aykanat et al. 2015; Ayllon et al. 2015; Cauwelier et al. 2018). More work needs to be done to improve the resolution of stock structure and phenotypic differences of declining Atlantic salmon populations.

While it is challenging to disentangle independent historical events such as stocking and biogeographical colonization events, especially with many extirpated populations, this research demonstrates the contemporary genetic diversity of Atlantic salmon populations on PEI. Beyond describing the extent of the unique genetic signature observed in northeast PEI (Moore et al. 2014a), this research found that the stocking history on PEI did not account for differences in genetic patterns found in all PEI salmon populations. There are potentially multiple genetic groups that could be resultant from the dynamic history of biogeography, glacial recession, and potential straying on PEI. This research generates important questions for future research on the dynamic genetic structure of remnant salmon populations in the province, whether stocking influences recruitment and how straying might play a role in the groupings seen here. Additionally, the study prompts important management considerations regarding the movement of Atlantic salmon between watersheds for stocking purposes. Further research on this topic will help to inform managers on which genetic groups would be most suitable to use in watersheds where recolonization efforts may take place.

## Author Contributions

**Carissa M. Grove:** conceptualization (equal), data curation (lead), formal analysis (lead), investigation (lead), methodology (lead), writing – original draft (lead), writing – review and editing (equal). **Scott D. Roloson:** conceptualization (equal), data curation (supporting), methodology (supporting), writing – original draft (supporting), writing – review and editing (equal). **Kyle M. Knysh:** formal analysis (supporting), methodology (supporting), writing – original draft (supporting), writing – review and editing (equal). **Scott A. Pavey:** formal analysis (supporting), methodology (supporting), supervision (equal), writing – review and editing (supporting). **David K. Cairns:** data curation (supporting), formal analysis (supporting), writing – review and editing (supporting). **Robert F. Gilmour Jr.:** funding acquisition (equal), writing – review and editing (supporting). **Michael R. van den Heuvel:** conceptualization (equal), formal analysis (supporting), funding acquisition (equal), methodology (supporting), supervision (lead), writing – original draft (supporting), writing – review and editing (equal).

## Conflicts of Interest

The authors declare no conflicts of interest.

## Supporting information


Appendix S1

Appendix S2

## Data Availability

Microsatellite genotypes can be found at https://doi.org/10.5061/dryad.xksn02vst. Stocking data is in [Link], [Link].

## APPENDIX 1

### 1.1

**TABLE A1:** Standardization rules for comparison of microsatellite data between Moore et al. (2014) and the present study.

Locus	Grove study to Moore et al. (2014)
**SSsp2210**	No Change
**SSsp2215**	Add 1bp
**Ssa197**	Subtract 5bp
**Ssa85**	Subtract 3bp
**SsaD486**	Add 4bp
**SsosI417**	Subtract 2bp
