# Supplementary material for: Population Genetics of Atlantic Salmon (Salmo salar) in Prince Edward Island, Canada
Source: Ecol Evol. 2025 May 14;15(5):e71285. doi: 10.1002/ece3.71285 (PMC12078067; doi:10.1002/ece3.71285)
Supplement: Supplementary file 2 — Appendix S2 [file ECE3-15-e71285-s002.zip › supp.docx]

Supplemental Tables and Figured below

| River name Year | Located at or near | Watershed | Stream | 1880 | 1881 | 1882 |
| --- | --- | --- | --- | --- | --- | --- |
| Broodstock origin^a,b^ |  | area (km^2^)^c^ | area (m^2^)^c^ | Mostly DR, some RP-NS | DR, WR, other PEI locations | Most, perhaps all, from DR |
| Run timing of broodstock |  |  |  |  |  |  |
| Young salmon were reared in semi-natural ponds |  |  |  | None | None | None |
| Stage |  |  |  | Fry | Fry | Fry |
| Tignish River | Tignish | 44.5 | 58,241 |  |  |  |
| Mill River (Cains+Carruthers Brooks) | Cascumpeque | 78.8 | 58,300 |  |  |  |
| Trout River (Coleman) | Coleman | 107.1 | 140,202 | 100,000 | 60,000 |  |
| Trout River (Tyne Valley) | Tyne Valley | 48.3 | 63,281 |  |  |  |
| Indian River | Indian River | 23.9 | 31,326 |  |  |  |
| Hy Brook, Morrisons Pond | Darnley |  |  |  |  |  |
| Trout River (Millvale) | Millvale | 53.3 | 69,787 |  |  |  |
| Hunter River | Hunter River | 88.8 | 116,259 |  |  |  |
| Wheatley River | Wheatley River | 58.0 | 75,914 |  |  |  |
| Black River | Brackley Point | 20.9 | 27,307 |  |  |  |
| Bells Creek (Gurneys River) | West Covehead | 28.9 | 37,819 |  |  |  |
| Winter River | Suffolk | 69.6 | 91,112 |  |  |  |
| Morell River | Morell | 170.6 | 237,176 | 100,000 | 60,000 |  |
| Marie River | Marie | 29.3 | 38,408 |  |  |  |
| Midgell River | Midgell | 63.8 | 83,532 |  |  |  |
| St. Peters River | St. Peters | 44.6 | 58,333 |  |  |  |
| Schooner Pond | St. Peters | 19.4 | 25,424 |  |  |  |
| McAskill Creek (Goose River) | Goose River | 10.6 | 13,876 |  |  |  |
| Naufrage River | Naufrage | 43.6 | 57,037 |  |  |  |
| Cross Creek | Hermanville | 44.3 | 57,992 |  |  |  |
| North Lake Creek | North Lake | 47.7 | 62,495 |  |  |  |
| Bakers River^e^ | Location uncertain |  |  |  |  |  |
| Black Pond Creek | Black Pond, Red Point | 14.3 | 18,751 |  |  |  |
| Souris River | Souris | 53.2 | 69,578 |  |  |  |
| Rollo Bay | Rollo Bay | 21.4 | 28,045 |  |  |  |
| Fortune River | Dingwells Mills | 75.4 | 98,652 |  |  |  |
| Cardigan River | Cardigan | 44.6 | 58,411 |  |  |  |
| Brudenell River | Brudenell | 55.3 | 72,379 |  | 60,000 |  |
| Montague River | Montague | 76.3 | 99,883 |  |  |  |
| Valleyfield River | Valleyfield | 87.7 | 127,500 |  |  |  |
| Sturgeon River | Sturgeon | 60.4 | 79,068 |  |  |  |
| Murray River | Murray River | 71.0 | 92,905 |  |  |  |
| Fox River | Murray River | 10.6 | 13,939 |  |  |  |
| Belle River | Belle River | 35.9 | 47,022 |  |  |  |
| Vernon River | Vernon Bridge | 69.2 | 90,536 |  |  |  |
| Forbes Creek (southeast branch of Fullertons Creek) | Mount Herbert |  |  |  |  |  |
| Johnstons River | Johnstons River | 39.3 | 51,421 |  |  |  |
| Glenfinnan River (Sherrys Creek) | Glenfinnan | 33.3 | 43,553 |  |  |  |
| Head of Hillsborough River | Mount Stewart | 53.1 | 69,512 |  |  |  |
| Hillsborough (East) River, unspecified location | Mount Stewart |  |  |  |  |  |
| North River | Milton | 99.0 | 129,651 |  |  |  |
| Clyde River | Clyde River | 41.7 | 54,549 |  |  |  |
| West River | Bonshaw | 114.1 | 184,500 |  |  |  |
| Desable River | Desable | 43.7 | 57,246 |  |  |  |
| Westmoreland River | Crapaud | 43.2 | 56,500 |  |  |  |
| Tryon River | Tryon | 56.4 | 73,767 |  |  |  |
| Dunk River | Freetown | 165.7 | 193,078 | 300,000 | 195,000 |  |
| Wilmot River | Wilmot Valley | 83.4 | 109,177 |  |  |  |
| Miminigash River | Miminegash | 26.7 | 34,939 |  |  |  |
| Skinners Pond | Skinners Pond | 8.8 | 11,534 |  |  |  |
| Nail Pond | Nail Pond | 16.4 | 21,525 |  |  |  |
| Curtisdale River^f^ | Location unknown |  |  |  |  |  |
| Inspector River | Location unknown |  |  |  |  |  |
| Mores River^g^ | Location unknown |  |  |  |  |  |
| Trout Newbarton | Location unknown |  |  |  |  |  |
| Breakdown by river not reported |  |  |  |  |  | 1,060,000 |
| Total number released |  |  |  | 500,000 | 375,000 | 1,060,000 |
| Total number released, annual sums only |  | 2,596 | 3,391,445 | 500,000 | 375,000 | 1,060,000 |

Table S1: Reported number of Atlantic salmon stocked in Prince Edward Island waters, 1880-2023

Table S1 continued

| River name Year | 1883 | 1884 | 1885 |  | 1887 | 1888 | 1889 | 1890 | 1891 |
| --- | --- | --- | --- | --- | --- | --- | --- | --- | --- |
| Broodstock origin^a,b^ | DR |  | DR | Possibly DR, possibly NS | DR |  |  |  |  |
| Run timing of broodstock |  |  |  |  |  |  |  |  |  |
| Young salmon were reared in semi-natural ponds | None | None | None | None | None |  |  |  |  |
| Stage | Fry | Fry | Fry | Fry | Fry |  |  |  |  |
| Tignish River |  |  |  |  |  |  |  |  |  |
| Mill River (Cains+Carruthers Brooks) |  |  |  |  |  |  |  |  |  |
| Trout River (Coleman) |  | x^d^ |  |  | 30,000 |  |  |  |  |
| Trout River (Tyne Valley) |  |  |  |  | 30,000 |  |  |  |  |
| Indian River |  |  |  |  |  |  |  |  |  |
| Hy Brook, Morrisons Pond |  |  |  |  |  |  |  |  |  |
| Trout River (Millvale) |  |  |  |  | 30,000 |  |  |  |  |
| Hunter River |  | x |  |  |  |  |  |  |  |
| Wheatley River |  | x |  |  |  |  |  |  |  |
| Black River |  |  |  |  |  |  |  |  |  |
| Bells Creek (Gurneys River) |  |  |  |  |  |  |  |  |  |
| Winter River |  |  |  |  |  |  |  |  |  |
| Morell River |  | x |  |  |  |  |  |  |  |
| Marie River |  |  |  |  |  |  |  |  |  |
| Midgell River |  |  |  |  |  |  |  |  |  |
| St. Peters River |  |  |  |  |  |  |  |  |  |
| Schooner Pond |  |  |  |  |  |  |  |  |  |
| McAskill Creek (Goose River) |  |  |  |  |  |  |  |  |  |
| Naufrage River |  |  |  |  |  |  |  |  |  |
| Cross Creek |  |  |  |  |  |  |  |  |  |
| North Lake Creek |  |  |  |  | 50,000 |  |  |  |  |
| Bakers River^e^ |  |  |  |  |  |  |  |  |  |
| Black Pond Creek |  |  |  |  |  |  |  |  |  |
| Souris River |  |  |  |  |  |  |  |  |  |
| Rollo Bay |  |  |  |  |  |  |  |  |  |
| Fortune River |  |  |  |  |  |  |  |  |  |
| Cardigan River |  |  |  |  |  |  |  |  |  |
| Brudenell River |  | x |  |  |  |  |  |  |  |
| Montague River |  | x |  |  |  |  |  |  |  |
| Valleyfield River |  |  |  |  |  |  |  |  |  |
| Sturgeon River |  |  |  |  |  |  |  |  |  |
| Murray River |  |  |  |  |  |  |  |  |  |
| Fox River |  |  |  |  |  |  |  |  |  |
| Belle River |  |  |  |  |  |  |  |  |  |
| Vernon River |  |  |  |  |  |  |  |  |  |
| Forbes Creek (southeast branch of Fullertons Creek) |  |  |  |  |  |  |  |  |  |
| Johnstons River |  |  |  |  | 50,000 |  |  |  |  |
| Glenfinnan River (Sherrys Creek) |  |  |  |  |  |  |  |  |  |
| Head of Hillsborough River |  |  |  |  |  |  |  |  |  |
| Hillsborough (East) River, unspecified location |  |  |  |  |  |  |  |  |  |
| North River |  |  |  |  |  |  |  |  |  |
| Clyde River |  |  |  |  |  |  |  |  |  |
| West River |  | x |  |  | 50,000 |  |  |  |  |
| Desable River |  |  |  |  | 30,000 |  |  |  |  |
| Westmoreland River |  |  |  |  | 30,000 |  |  |  |  |
| Tryon River |  |  |  |  | 10,000 |  |  |  |  |
| Dunk River |  | x |  |  | 100,000 |  |  |  |  |
| Wilmot River |  | x |  |  | 80,000 |  |  |  |  |
| Miminigash River |  |  |  |  |  |  |  |  |  |
| Skinners Pond |  |  |  |  |  |  |  |  |  |
| Nail Pond |  |  |  |  |  |  |  |  |  |
| Curtisdale River^f^ |  |  |  |  |  |  |  |  |  |
| Inspector River |  |  |  |  | 10,000 |  |  |  |  |
| Mores River^g^ |  |  |  |  |  |  |  |  |  |
| Trout Newbarton |  | x |  |  |  |  |  |  |  |
| Breakdown by river not reported | 1,210,000 | 1,000,000 | 1,100,000 | 400,000 |  |  |  |  |  |
| Total number released | 1,210,000 | 1,000,000 | 1,100,000 | 400,000 | 500,000 |  |  |  |  |
| Total number released, annual sums only | 1,210,000 | 1,000,000 | 1,100,000 | 400,000 | 500,000 |  |  |  |  |

Table S1 continued

| River name Year | 1892 | 1893 | 1894 | 1895 | 1896 | 1897 | 1898 | 1899 | 1900 |
| --- | --- | --- | --- | --- | --- | --- | --- | --- | --- |
| Broodstock origin^a,b^ |  |  |  |  |  |  |  | SJ-NB, MR-NB | SJ-NB |
| Run timing of broodstock |  |  |  |  |  |  |  |  |  |
| Young salmon were reared in semi-natural ponds |  |  |  |  |  |  |  | None | None |
| Stage |  |  |  |  |  |  |  | Fry | Fry |
| Tignish River |  |  |  |  |  |  |  |  |  |
| Mill River (Cains+Carruthers Brooks) |  |  |  |  |  |  |  |  |  |
| Trout River (Coleman) |  |  |  |  |  |  |  |  |  |
| Trout River (Tyne Valley) |  |  |  |  |  |  |  |  |  |
| Indian River |  |  |  |  |  |  |  |  |  |
| Hy Brook, Morrisons Pond |  |  |  |  |  |  |  |  |  |
| Trout River (Millvale) |  |  |  |  |  |  |  |  |  |
| Hunter River |  |  |  |  |  |  |  |  |  |
| Wheatley River |  |  |  |  |  |  |  |  | 75,000 |
| Black River |  |  |  |  |  |  |  |  |  |
| Bells Creek (Gurneys River) |  |  |  |  |  |  |  |  |  |
| Winter River |  |  |  |  |  |  |  |  |  |
| Morell River |  |  |  |  |  |  |  |  | 75,000 |
| Marie River |  |  |  |  |  |  |  |  |  |
| Midgell River |  |  |  |  |  |  |  |  |  |
| St. Peters River |  |  |  |  |  |  |  |  |  |
| Schooner Pond |  |  |  |  |  |  |  |  |  |
| McAskill Creek (Goose River) |  |  |  |  |  |  |  |  |  |
| Naufrage River |  |  |  |  |  |  |  |  | 75,000 |
| Cross Creek |  |  |  |  |  |  |  |  |  |
| North Lake Creek |  |  |  |  |  |  |  |  |  |
| Bakers River^e^ |  |  |  |  |  |  |  |  |  |
| Black Pond Creek |  |  |  |  |  |  |  |  |  |
| Souris River |  |  |  |  |  |  |  |  |  |
| Rollo Bay |  |  |  |  |  |  |  |  |  |
| Fortune River |  |  |  |  |  |  |  |  |  |
| Cardigan River |  |  |  |  |  |  |  |  |  |
| Brudenell River |  |  |  |  |  |  |  |  |  |
| Montague River |  |  |  |  |  |  |  |  |  |
| Valleyfield River |  |  |  |  |  |  |  |  |  |
| Sturgeon River |  |  |  |  |  |  |  |  |  |
| Murray River |  |  |  |  |  |  |  | 25,000 |  |
| Fox River |  |  |  |  |  |  |  | 25,000 |  |
| Belle River |  |  |  |  |  |  |  |  |  |
| Vernon River |  |  |  |  |  |  |  | 75,000 |  |
| Forbes Creek (southeast branch of Fullertons Creek) |  |  |  |  |  |  |  |  |  |
| Johnstons River |  |  |  |  |  |  |  |  |  |
| Glenfinnan River (Sherrys Creek) |  |  |  |  |  |  |  |  |  |
| Head of Hillsborough River |  |  |  |  |  |  |  |  |  |
| Hillsborough (East) River, unspecified location |  |  |  |  |  |  |  |  |  |
| North River |  |  |  |  |  |  |  |  |  |
| Clyde River |  |  |  |  |  |  |  |  |  |
| West River |  |  |  |  |  |  |  |  |  |
| Desable River |  |  |  |  |  |  |  |  |  |
| Westmoreland River |  |  |  |  |  |  |  |  |  |
| Tryon River |  |  |  |  |  |  |  |  |  |
| Dunk River |  |  |  |  |  |  |  |  |  |
| Wilmot River |  |  |  |  |  |  |  |  |  |
| Miminigash River |  |  |  |  |  |  |  |  |  |
| Skinners Pond |  |  |  |  |  |  |  |  |  |
| Nail Pond |  |  |  |  |  |  |  |  |  |
| Curtisdale River^f^ |  |  |  |  |  |  |  |  |  |
| Inspector River |  |  |  |  |  |  |  |  |  |
| Mores River^g^ |  |  |  |  |  |  |  |  |  |
| Trout Newbarton |  |  |  |  |  |  |  |  |  |
| Breakdown by river not reported |  |  |  |  |  |  |  |  |  |
| Total number released |  |  |  |  |  |  |  | 125,000 | 225,000 |
| Total number released, annual sums only |  |  |  |  |  |  |  | 125,000 | 225,000 |
|  |  |  |  |  |  |  |  |  |  |

| River name Year | 1901 | 1902 | 1903 | 1904 | 1905 | 1906 | 1907 | 1908 | 1909 |
| --- | --- | --- | --- | --- | --- | --- | --- | --- | --- |
| Broodstock origin^a,b^ |  |  |  |  |  |  | SJ-NB, MR-NB | MR-NB | MR-NB |
| Run timing of broodstock |  |  |  |  |  |  |  |  |  |
| Young salmon were reared in semi-natural ponds |  |  |  |  |  | None | None | None | None |
| Stage |  |  |  |  |  | Fry | Fry | Fry | Fry |
| Tignish River |  |  |  |  |  |  |  |  |  |
| Mill River (Cains+Carruthers Brooks) |  |  |  |  |  |  |  |  |  |
| Trout River (Coleman) |  |  |  |  |  |  |  |  | 72,000 |
| Trout River (Tyne Valley) |  |  |  |  |  |  |  |  | 72,000 |
| Indian River |  |  |  |  |  |  |  |  |  |
| Hy Brook, Morrisons Pond |  |  |  |  |  |  |  |  |  |
| Trout River (Millvale) |  |  |  |  |  |  |  |  |  |
| Hunter River |  |  |  |  |  |  |  |  |  |
| Wheatley River |  |  |  |  |  | 100,000 | 60,000 | 60,000 | 72,000 |
| Black River |  |  |  |  |  |  | 60,000 |  | 72,000 |
| Bells Creek (Gurneys River) |  |  |  |  |  |  |  |  |  |
| Winter River |  |  |  |  |  | 300,000 | 160,000 | 200,000 | 370,000 |
| Morell River |  |  |  |  |  | 200,000 | 140,000 | 200,000 | 226,000 |
| Marie River |  |  |  |  |  |  |  |  |  |
| Midgell River |  |  |  |  |  |  | 140,000 |  | 72,000 |
| St. Peters River |  |  |  |  |  |  |  |  |  |
| Schooner Pond |  |  |  |  |  |  |  |  |  |
| McAskill Creek (Goose River) |  |  |  |  |  |  |  |  |  |
| Naufrage River |  |  |  |  |  |  |  | 100,000 |  |
| Cross Creek |  |  |  |  |  |  |  |  |  |
| North Lake Creek |  |  |  |  |  |  |  | 30,000 |  |
| Bakers River^e^ |  |  |  |  |  |  |  |  |  |
| Black Pond Creek |  |  |  |  |  |  |  |  |  |
| Souris River |  |  |  |  |  |  |  |  |  |
| Rollo Bay |  |  |  |  |  |  |  |  |  |
| Fortune River |  |  |  |  |  |  | 60,000 | 100,000 |  |
| Cardigan River |  |  |  |  |  |  |  |  |  |
| Brudenell River |  |  |  |  |  |  |  |  |  |
| Montague River |  |  |  |  |  |  |  |  |  |
| Valleyfield River |  |  |  |  |  |  |  |  |  |
| Sturgeon River |  |  |  |  |  |  |  |  |  |
| Murray River |  |  |  |  |  |  | 60,000 | 80,000 | 72,000 |
| Fox River |  |  |  |  |  |  |  |  |  |
| Belle River |  |  |  |  |  |  |  |  |  |
| Vernon River |  |  |  |  |  |  |  |  |  |
| Forbes Creek (southeast branch of Fullertons Creek) |  |  |  |  |  |  |  |  |  |
| Johnstons River |  |  |  |  |  |  |  |  |  |
| Glenfinnan River (Sherrys Creek) |  |  |  |  |  |  |  |  |  |
| Head of Hillsborough River |  |  |  |  |  |  |  |  |  |
| Hillsborough (East) River, unspecified location |  |  |  |  |  |  |  |  |  |
| North River |  |  |  |  |  |  | 30,000 | 50,000 |  |
| Clyde River |  |  |  |  |  |  |  |  | 72,000 |
| West River |  |  |  |  |  |  |  |  |  |
| Desable River |  |  |  |  |  |  |  |  |  |
| Westmoreland River |  |  |  |  |  |  |  |  |  |
| Tryon River |  |  |  |  |  |  |  |  |  |
| Dunk River |  |  |  |  |  | 100,000 | 80,000 | 80,000 | 72,000 |
| Wilmot River |  |  |  |  |  |  |  |  |  |
| Miminigash River |  |  |  |  |  |  |  |  |  |
| Skinners Pond |  |  |  |  |  |  |  |  |  |
| Nail Pond |  |  |  |  |  |  |  |  |  |
| Curtisdale River^f^ |  |  |  |  |  |  |  |  |  |
| Inspector River |  |  |  |  |  |  |  |  |  |
| Mores River^g^ |  |  |  |  |  | 20,000 |  |  |  |
| Trout Newbarton |  |  |  |  |  |  |  |  |  |
| Breakdown by river not reported |  |  |  |  |  |  |  |  |  |
| Total number released |  |  |  |  |  | 720,000 | 790,000 | 900,000 | 1,172,000 |
| Total number released, annual sums only |  |  |  |  |  | 720,000 | 790,000 | 900,000 | 1,172,000 |

Table S1 continued

Table S1 continued

| River name Year | 1910 | 1911 | 1912 | 1913 | 1914 | 1915 | 1915 | 1915 |
| --- | --- | --- | --- | --- | --- | --- | --- | --- |
| Broodstock origin^a,b^ | MR-NB | MR-NB |  | MR-NB | MR-NB | MR-NB | MR-NB | MR-NB |
| Run timing of broodstock |  |  |  |  |  |  |  |  |
| Young salmon were reared in semi-natural ponds | None | None |  | None | None | None | None | None |
| Stage | Fry | Fry |  | Fry | Fry | Fry | Finger- lings | Total, all stages |
| Tignish River |  |  |  |  |  |  |  |  |
| Mill River (Cains+Carruthers Brooks) |  |  |  |  |  |  |  |  |
| Trout River (Coleman) |  | 63,000 |  |  |  |  |  |  |
| Trout River (Tyne Valley) |  |  |  |  |  |  |  |  |
| Indian River |  | 63,000 |  | 72,000 | 80,000 | 60,000 |  | 60,000 |
| Hy Brook, Morrisons Pond |  |  |  |  |  |  |  |  |
| Trout River (Millvale) |  |  |  |  |  |  |  |  |
| Hunter River |  |  |  |  |  |  |  |  |
| Wheatley River |  |  |  | 72,000 | 80,000 | 70,000 |  | 70,000 |
| Black River | 72,000 |  |  |  |  |  |  |  |
| Bells Creek (Gurneys River) |  |  |  | 72,000 |  |  |  |  |
| Winter River | 432,000 | 189,000 |  | 72,000 | 80,000 | 100,000 | 24,909 | 124,909 |
| Morell River | 288,000 | 315,000 |  | 216,000 | 240,000 | 230,000 |  | 230,000 |
| Marie River |  |  |  |  |  |  |  |  |
| Midgell River | 72,000 |  |  | 72,000 | 80,000 |  |  |  |
| St. Peters River |  |  |  |  |  |  |  |  |
| Schooner Pond |  |  |  |  |  |  |  |  |
| McAskill Creek (Goose River) |  |  |  |  |  |  |  |  |
| Naufrage River |  |  |  |  |  |  |  |  |
| Cross Creek |  |  |  |  |  |  |  |  |
| North Lake Creek |  |  |  |  |  |  |  |  |
| Bakers River^e^ |  | 252,000 |  |  |  |  |  |  |
| Black Pond Creek |  |  |  |  |  |  |  |  |
| Souris River |  |  |  |  |  |  |  |  |
| Rollo Bay |  |  |  |  |  |  |  |  |
| Fortune River |  |  |  |  |  |  |  |  |
| Cardigan River |  |  |  |  |  |  |  |  |
| Brudenell River |  |  |  |  |  |  |  |  |
| Montague River |  |  |  |  |  |  |  |  |
| Valleyfield River |  |  |  |  |  |  |  |  |
| Sturgeon River |  |  |  |  |  |  |  |  |
| Murray River | 72,000 | 63,000 |  |  |  |  |  |  |
| Fox River |  |  |  |  |  |  |  |  |
| Belle River |  |  |  | 200,000 | 80,000 | 60,000 |  | 60,000 |
| Vernon River |  |  |  |  |  |  |  |  |
| Forbes Creek (southeast branch of Fullertons Creek) |  |  |  |  | 60,000 |  |  |  |
| Johnstons River |  |  |  |  |  |  |  |  |
| Glenfinnan River (Sherrys Creek) |  |  |  | 72,000 |  |  |  |  |
| Head of Hillsborough River |  |  |  |  | 80,000 | 60,000 |  | 60,000 |
| Hillsborough (East) River, unspecified location |  |  |  |  |  |  |  |  |
| North River | 72,000 | 189,000 |  | 144,000 | 160,000 | 60,000 |  | 60,000 |
| Clyde River | 72,000 |  |  |  |  |  |  |  |
| West River |  |  |  |  | 80,000 | 70,000 |  | 70,000 |
| Desable River |  |  |  |  |  |  |  |  |
| Westmoreland River |  |  |  |  |  |  |  |  |
| Tryon River |  |  |  |  |  |  |  |  |
| Dunk River | 72,000 | 63,000 |  | 72,000 | 100,000 | 160,000 |  | 160,000 |
| Wilmot River |  |  |  |  |  |  |  |  |
| Miminigash River |  |  |  |  |  |  |  |  |
| Skinners Pond |  |  |  |  |  |  |  |  |
| Nail Pond |  |  |  |  |  |  |  |  |
| Curtisdale River^f^ |  |  |  |  |  |  |  |  |
| Inspector River |  |  |  |  |  |  |  |  |
| Mores River^g^ |  |  |  |  |  |  |  |  |
| Trout Newbarton |  |  |  |  |  |  |  |  |
| Breakdown by river not reported |  |  |  |  |  |  |  |  |
| Total number released | 1,152,000 | 1,197,000 |  | 1,064,000 | 1,120,000 | 870,000 | 24,909 | 894,909 |
| Total number released, annual sums only | 1,152,000 | 1,197,000 |  | 1,064,000 | 1,120,000 |  |  | 894,909 |

Table S1 continued

| River name Year | 1916 | 1916 | 1917 | 1918 | 1919 | 1920 | 1921 | 1922 |
| --- | --- | --- | --- | --- | --- | --- | --- | --- |
| Broodstock origin^a,b^ | MR-NB | MR-NB |  |  |  |  |  |  |
| Run timing of broodstock |  |  |  |  |  |  |  |  |
| Young salmon were reared in semi-natural ponds | None | None | None | None | None |  |  |  |
| Stage | Finger- lings | Total, all stages | Fry | Fry | Fry |  |  |  |
| Tignish River |  |  |  |  |  |  |  |  |
| Mill River (Cains+Carruthers Brooks) |  |  |  |  |  |  |  |  |
| Trout River (Coleman) |  |  |  |  |  |  |  |  |
| Trout River (Tyne Valley) |  |  |  |  |  |  |  |  |
| Indian River |  |  |  |  |  |  |  |  |
| Hy Brook, Morrisons Pond |  |  |  |  |  |  |  |  |
| Trout River (Millvale) |  |  |  |  |  |  |  |  |
| Hunter River |  | 40,000 |  |  |  |  |  |  |
| Wheatley River |  |  |  |  |  |  |  |  |
| Black River |  |  |  |  |  |  |  |  |
| Bells Creek (Gurneys River) |  |  |  |  |  |  |  |  |
| Winter River |  | 67,500 |  |  |  |  |  |  |
| Morell River |  | 120,000 |  |  |  |  |  |  |
| Marie River |  |  |  |  |  |  |  |  |
| Midgell River |  | 67,500 |  |  |  |  |  |  |
| St. Peters River |  |  |  |  |  |  |  |  |
| Schooner Pond |  |  |  |  |  |  |  |  |
| McAskill Creek (Goose River) |  |  |  |  |  |  |  |  |
| Naufrage River |  |  |  |  |  |  |  |  |
| Cross Creek |  |  |  |  |  |  |  |  |
| North Lake Creek |  |  |  |  |  |  |  |  |
| Bakers River^e^ |  |  |  |  |  |  |  |  |
| Black Pond Creek |  |  |  |  |  |  |  |  |
| Souris River |  |  |  |  |  |  |  |  |
| Rollo Bay |  |  |  |  |  |  |  |  |
| Fortune River |  |  |  |  |  |  |  |  |
| Cardigan River |  |  |  |  |  |  |  |  |
| Brudenell River |  |  |  |  |  |  |  |  |
| Montague River |  |  |  |  |  |  |  |  |
| Valleyfield River |  |  |  |  |  |  |  |  |
| Sturgeon River |  |  |  |  |  |  |  |  |
| Murray River |  |  |  |  |  |  |  |  |
| Fox River |  |  |  |  |  |  |  |  |
| Belle River |  | 40,000 |  |  |  |  |  |  |
| Vernon River |  |  |  |  |  |  |  |  |
| Forbes Creek (southeast branch of Fullertons Creek) |  |  |  |  |  |  |  |  |
| Johnstons River | 1,000 | 1,000 |  |  |  |  |  |  |
| Glenfinnan River (Sherrys Creek) |  | 67,500 |  |  |  |  |  |  |
| Head of Hillsborough River |  |  |  |  |  |  |  |  |
| Hillsborough (East) River, unspecified location |  |  |  |  |  |  |  |  |
| North River |  | 95,500 |  |  |  |  |  |  |
| Clyde River |  |  |  |  |  |  |  |  |
| West River |  | 67,500 |  |  |  |  |  |  |
| Desable River |  |  |  |  |  |  |  |  |
| Westmoreland River |  |  |  |  |  |  |  |  |
| Tryon River |  |  |  |  |  |  |  |  |
| Dunk River |  |  |  |  |  |  |  |  |
| Wilmot River |  |  |  |  |  |  |  |  |
| Miminigash River |  |  |  |  |  |  |  |  |
| Skinners Pond |  |  |  |  |  |  |  |  |
| Nail Pond |  |  |  |  |  |  |  |  |
| Curtisdale River^f^ | 355 | 355 |  |  |  |  |  |  |
| Inspector River |  |  |  |  |  |  |  |  |
| Mores River^g^ | 1,000 | 1,000 |  |  |  |  |  |  |
| Trout Newbarton |  |  |  |  |  |  |  |  |
| Breakdown by river not reported |  |  |  |  |  |  |  |  |
| Total number released | 2,355 | 567,855 |  |  |  |  |  |  |
| Total number released, annual sums only |  | 567,855 |  |  |  |  |  |  |

Table S1 continued

| River name Year | 1923 | 1924 | 1925 | 1926 | 1927 | 1928 | 1929 | 1929 |
| --- | --- | --- | --- | --- | --- | --- | --- | --- |
| Broodstock origin^a,b^ |  |  | Probably MR | Probably MR | Probably MR | Probably MR | Probably MR | Probably MR |
| Run timing of broodstock |  |  |  |  |  |  |  |  |
| Young salmon were reared in semi-natural ponds |  |  | None | None | None | None | None | None |
| Stage |  |  |  |  | Fry | Fry | Fry | Finger- lings |
| Tignish River |  |  |  |  |  |  |  |  |
| Mill River (Cains+Carruthers Brooks) |  |  |  |  |  |  |  |  |
| Trout River (Coleman) |  |  |  |  |  |  |  |  |
| Trout River (Tyne Valley) |  |  |  |  | 15,000 |  |  |  |
| Indian River |  |  | 40,000 |  |  | 25,000 |  |  |
| Hy Brook, Morrisons Pond |  |  |  |  |  |  |  |  |
| Trout River (Millvale) |  |  |  |  |  |  |  |  |
| Hunter River |  |  |  |  |  | 15,000 |  |  |
| Wheatley River |  |  |  |  |  |  |  |  |
| Black River |  |  |  |  |  |  |  |  |
| Bells Creek (Gurneys River) |  |  |  |  |  |  |  |  |
| Winter River |  |  | 90,000 | 49,600 | 80,000 | 75,000 | 35,520 |  |
| Morell River |  |  | 234,987 | 160,000 | 222,900 | 207,653 | 101,040 | 2,567 |
| Marie River |  |  |  |  |  |  |  |  |
| Midgell River |  |  |  |  | 15,000 | 15,000 |  |  |
| St. Peters River |  |  | 50,000 | 30,000 | 50,000 | 15,000 |  |  |
| Schooner Pond |  |  |  |  | 15,000 | 10,000 | 15,000 |  |
| McAskill Creek (Goose River) |  |  |  |  |  |  |  |  |
| Naufrage River |  |  | 50,000 | 30,000 | 50,000 | 36,000 | 18,000 |  |
| Cross Creek |  |  |  |  |  |  |  |  |
| North Lake Creek |  |  |  |  |  |  |  |  |
| Bakers River^e^ |  |  |  |  |  |  |  |  |
| Black Pond Creek |  |  |  |  |  |  |  |  |
| Souris River |  |  |  |  |  |  |  |  |
| Rollo Bay |  |  |  |  |  |  |  |  |
| Fortune River |  |  | 32,000 |  | 25,000 | 15,000 |  |  |
| Cardigan River |  |  | 50,000 | 30,000 | 25,000 | 25,000 | 18,000 |  |
| Brudenell River |  |  |  |  |  | 10,000 |  |  |
| Montague River |  |  |  |  |  |  |  |  |
| Valleyfield River |  |  |  |  |  |  |  |  |
| Sturgeon River |  |  |  |  |  |  |  |  |
| Murray River |  |  |  |  |  |  |  |  |
| Fox River |  |  |  |  |  |  |  |  |
| Belle River |  |  | 30,000 |  |  | 15,000 |  |  |
| Vernon River |  |  |  |  |  |  |  |  |
| Forbes Creek (southeast branch of Fullertons Creek) |  |  |  |  | 2,000 |  |  |  |
| Johnstons River |  |  |  |  | 25,000 | 15,000 |  |  |
| Glenfinnan River (Sherrys Creek) |  |  |  | 9,000 |  |  |  |  |
| Head of Hillsborough River |  |  | 50,000 | 30,000 |  | 25,000 | 33,000 |  |
| Hillsborough (East) River, unspecified location |  |  |  |  |  |  |  |  |
| North River |  |  |  | 12,600 | 20,000 | 15,000 | 25,000 |  |
| Clyde River |  |  |  |  |  |  |  |  |
| West River |  |  | 30,000 | 12,600 |  |  |  |  |
| Desable River |  |  |  |  |  |  |  |  |
| Westmoreland River |  |  |  |  |  |  |  |  |
| Tryon River |  |  |  |  |  |  |  |  |
| Dunk River |  |  | 90,000 | 90,000 | 150,000 | 100,000 | 60,000 |  |
| Wilmot River |  |  |  | 25,000 |  |  |  |  |
| Miminigash River |  |  |  |  |  |  |  |  |
| Skinners Pond |  |  |  |  |  |  |  |  |
| Nail Pond |  |  |  |  |  |  |  |  |
| Curtisdale River^f^ |  |  |  |  |  |  |  |  |
| Inspector River |  |  |  |  |  |  |  |  |
| Mores River^g^ |  |  |  |  |  |  |  |  |
| Trout Newbarton |  |  |  |  |  |  |  |  |
| Breakdown by river not reported |  |  |  |  |  |  |  |  |
| Total number released |  |  | 746,987 | 478,800 | 694,900 | 618,653 | 305,560 | 2,567 |
| Total number released, annual sums only |  |  | 746,987 | 478,800 | 694,900 | 618,653 |  |  |

Table S1 continued

| River name Year | 1929 | 1930 | 1931 | 1932 | 1933 | 1934 | 1935 | 1936 |
| --- | --- | --- | --- | --- | --- | --- | --- | --- |
| Broodstock origin^a,b^ | Probably MR | Probably MR | Probably from MR | Probably MR | Probably MR | Probably MR | Probably MR | Probably MR |
| Run timing of broodstock |  |  |  |  |  |  |  |  |
| Young salmon were reared in semi-natural ponds | None | None | None | None | None | None | None | None |
| Stage | Total, all stages | Fry |  |  | Advanced fry |  |  |  |
| Tignish River |  | 31,680 | 49,500 | 20,000 |  |  |  |  |
| Mill River (Cains+Carruthers Brooks) |  |  |  | 20,300 |  |  |  |  |
| Trout River (Coleman) |  |  |  |  |  |  |  |  |
| Trout River (Tyne Valley) |  |  |  |  |  |  |  |  |
| Indian River |  |  |  |  |  |  |  |  |
| Hy Brook, Morrisons Pond |  |  |  | 200 |  |  |  |  |
| Trout River (Millvale) |  |  |  |  |  |  |  |  |
| Hunter River |  |  |  |  |  |  |  |  |
| Wheatley River |  |  |  |  |  |  |  |  |
| Black River |  |  |  |  |  |  |  |  |
| Bells Creek (Gurneys River) |  |  |  |  | 30,000 | 40,000 | 18,400 |  |
| Winter River | 35,520 | 92,800 | 103,340 | 50,000 | 100,000 | 60,000 | 50,400 | 20,000 |
| Morell River | 103,607 | 76,720 | 503,436 | 242,622 | 338,054 | 349,835 | 299,608 | 609,000 |
| Marie River |  |  |  |  |  |  |  |  |
| Midgell River |  | 14,000 | 74,500 | 25,000 | 50,000 | 40,500 | 28,800 | 50,000 |
| St. Peters River |  | 25,320 | 45,000 | 25,000 | 32,000 | 30,000 | 28,800 | 50,000 |
| Schooner Pond | 15,000 | 14,000 | 25,000 | 24,000 | 25,000 | 36,000 | 38,400 | 48,000 |
| McAskill Creek (Goose River) |  |  |  |  |  |  |  | 25,000 |
| Naufrage River | 18,000 | 27,840 | 102,000 | 40,300 | 97,000 | 40,500 | 28,800 | 93,000 |
| Cross Creek |  | 8,120 | 18,000 |  |  |  |  |  |
| North Lake Creek |  | 8,120 |  |  |  |  |  | 25,000 |
| Bakers River^e^ |  |  |  |  |  |  |  |  |
| Black Pond Creek |  |  |  |  | 25,000 | 23,000 | 20,000 |  |
| Souris River |  |  |  |  |  | 40,000 | 22,800 |  |
| Rollo Bay |  |  |  |  | 30,000 |  |  |  |
| Fortune River |  |  |  |  | 47,300 | 30,000 | 28,800 | 40,000 |
| Cardigan River | 18,000 | 25,200 | 50,000 | 23,400 | 24,000 |  |  | 60,085 |
| Brudenell River |  |  |  |  |  |  |  |  |
| Montague River |  |  |  |  |  | 40,500 | 50,000 | 85,000 |
| Valleyfield River |  |  |  |  |  |  |  |  |
| Sturgeon River |  |  |  |  | 32,000 | 38,000 | 28,800 | 25,000 |
| Murray River |  |  | 10,000 |  |  |  |  |  |
| Fox River |  |  |  |  |  |  |  |  |
| Belle River |  |  |  |  |  |  |  |  |
| Vernon River |  |  |  |  |  |  |  |  |
| Forbes Creek (southeast branch of Fullertons ) |  |  |  |  |  |  |  |  |
| Johnstons River |  | 28,120 | 25,000 | 20,000 | 30,000 |  |  |  |
| Glenfinnan River (Sherrys Creek) |  |  |  |  |  |  |  |  |
| Head of Hillsborough River | 33,000 | 25,320 | 50,000 | 24,000 | 35,000 | 40,500 | 38,400 | 50,000 |
| Hillsborough (East) River, unspecified location |  |  |  |  |  |  |  |  |
| North River | 25,000 |  |  |  | 32,000 | 40,000 | 38,400 |  |
| Clyde River |  |  |  |  |  |  |  |  |
| West River |  |  |  |  |  |  |  |  |
| Desable River |  |  |  |  |  |  |  |  |
| Westmoreland River |  |  |  |  |  |  |  |  |
| Tryon River |  |  |  |  |  |  |  |  |
| Dunk River | 60,000 | 80,200 | 200,000 | 100,000 | 96,000 |  |  |  |
| Wilmot River |  |  |  |  |  |  |  |  |
| Miminigash River |  |  |  | 20,300 | 61,600 | 80,500 | 33,600 |  |
| Skinners Pond |  |  |  |  | 17,800 | 23,000 |  |  |
| Nail Pond |  |  |  |  | 17,800 | 23,000 | 20,000 |  |
| Curtisdale River^f^ |  |  |  |  |  |  |  |  |
| Inspector River |  |  |  |  |  |  |  |  |
| Mores River^g^ |  |  |  |  |  |  |  |  |
| Trout Newbarton |  |  |  |  |  |  |  |  |
| Breakdown by river not reported |  |  |  |  |  |  |  |  |
| Total number released | 308,127 | 457,440 | 1,255,776 | 635,122 | 1,120,554 | 975,335 | 774,008 | 1,180,085 |
| Total number released, annual sums only | 308,127 | 457,440 | 1,255,776 | 635,122 | 1,120,554 | 975,335 | 774,008 | 1,180,085 |

Table S1 continued

| River name Year | 1937 | 1938 | 1939 | 1940 | 1941 | 1942 | 1943 | 1944 |
| --- | --- | --- | --- | --- | --- | --- | --- | --- |
| Broodstock origin^a,b^ | Probably MR | Probably MR | Probably MR | Probably MR | Probably MR | Probably MR | Probably MR | Probably MR |
| Run timing of broodstock |  |  |  |  |  |  |  |  |
| Young salmon were reared in semi-natural ponds | None | None | None | None | None | None | None | None |
| Stage |  |  |  |  |  |  |  |  |
| Tignish River |  |  |  |  |  |  |  |  |
| Mill River (Cains+Carruthers Brooks) |  |  |  |  |  |  |  |  |
| Trout River (Coleman) |  |  |  |  |  |  |  |  |
| Trout River (Tyne Valley) |  |  |  |  |  |  |  |  |
| Indian River |  |  |  |  |  |  |  |  |
| Hy Brook, Morrisons Pond |  |  |  |  |  |  |  |  |
| Trout River (Millvale) |  |  |  |  |  |  |  |  |
| Hunter River |  |  |  |  |  |  |  |  |
| Wheatley River |  |  |  |  |  |  |  |  |
| Black River |  |  |  |  |  |  |  |  |
| Bells Creek (Gurneys River) |  |  |  |  |  |  |  |  |
| Winter River | 15,000 |  |  |  |  |  |  |  |
| Morell River | 431,970 | 388,680 | 403,900 | 246,841 | 341,080 | 341,310 | 375,600 | 170,835 |
| Marie River |  |  |  | 20,000 | 40,000 | 24,000 | 30,000 | 10,000 |
| Midgell River | 66,000 |  |  | 25,000 | 50,000 | 115,000 | 80,000 | 37,000 |
| St. Peters River | 51,000 | 34,920 |  | 25,000 | 50,000 | 30,000 | 30,000 | 25,000 |
| Schooner Pond | 30,000 | 53,200 |  |  |  |  |  |  |
| McAskill Creek (Goose River) | 30,000 |  |  |  |  |  |  |  |
| Naufrage River | 116,000 | 103,000 |  |  |  |  |  |  |
| Cross Creek | 50,000 |  |  |  |  |  |  |  |
| North Lake Creek |  |  |  |  |  |  |  |  |
| Bakers River^e^ |  |  |  |  |  |  |  |  |
| Black Pond Creek |  |  |  |  |  |  |  |  |
| Souris River |  |  |  |  |  |  |  |  |
| Rollo Bay |  |  |  |  |  |  |  |  |
| Fortune River |  |  |  |  |  |  |  |  |
| Cardigan River |  |  |  |  | 50,000 | 60,000 |  |  |
| Brudenell River |  |  |  |  |  |  |  |  |
| Montague River | 81,600 |  |  |  |  |  |  |  |
| Valleyfield River |  |  |  |  |  |  |  |  |
| Sturgeon River | 35,000 |  |  |  |  |  |  |  |
| Murray River |  |  |  |  |  |  |  |  |
| Fox River |  |  |  |  |  |  |  |  |
| Belle River |  |  |  |  |  |  |  |  |
| Vernon River |  |  |  |  |  |  |  |  |
| Forbes Creek (southeast branch of Fullertons ) |  |  |  |  |  |  |  |  |
| Johnstons River |  |  |  |  |  |  |  |  |
| Glenfinnan River (Sherrys Creek) |  |  |  |  |  |  |  |  |
| Head of Hillsborough River | 51,000 |  |  |  |  |  |  |  |
| Hillsborough (East) River, unspecified location |  |  |  |  |  |  |  |  |
| North River |  |  |  |  |  |  |  |  |
| Clyde River |  |  |  |  |  |  |  |  |
| West River |  |  |  |  |  |  |  |  |
| Desable River |  |  |  |  |  |  |  |  |
| Westmoreland River |  |  |  |  |  |  |  |  |
| Tryon River |  |  |  |  |  |  |  |  |
| Dunk River |  |  | 320,000 |  |  |  |  |  |
| Wilmot River |  |  |  |  |  |  |  |  |
| Miminigash River |  |  |  |  |  |  |  |  |
| Skinners Pond |  |  |  |  |  |  |  |  |
| Nail Pond |  |  |  |  |  |  |  |  |
| Curtisdale River^f^ |  |  |  |  |  |  |  |  |
| Inspector River |  |  |  |  |  |  |  |  |
| Mores River^g^ |  |  |  |  |  |  |  |  |
| Trout Newbarton |  |  |  |  |  |  |  |  |
| Breakdown by river not reported |  |  |  |  |  |  |  |  |
| Total number released | 957,570 | 579,800 | 723,900 | 316,841 | 531,080 | 570,310 | 515,600 | 242,835 |
| Total number released, annual sums only | 957,570 | 579,800 | 723,900 | 316,841 | 531,080 | 570,310 | 515,600 | 242,835 |

Table S1 continued

| River name Year | 1945 | 1946 | 1947 | 1948 | 1949 | 1950 | 1951 | 1952 |
| --- | --- | --- | --- | --- | --- | --- | --- | --- |
| Broodstock origin^a,b^ | Probably MR | Probably MR | Probably MR | Probably MR | Probably MR | Probably MR | Probably MR |  |
| Run timing of broodstock |  |  |  |  |  |  |  |  |
| Young salmon were reared in semi-natural ponds | None | None | None | None | None | None | None | None |
| Stage | Fry |  |  |  |  | Fry and advanced fry |  | Fry |
| Tignish River |  |  |  |  |  |  |  |  |
| Mill River (Cains+Carruthers Brooks) |  |  |  |  |  |  |  |  |
| Trout River (Coleman) |  |  |  |  |  |  |  |  |
| Trout River (Tyne Valley) |  |  |  |  |  |  |  |  |
| Indian River |  |  |  |  |  |  |  |  |
| Hy Brook, Morrisons Pond |  |  |  |  |  |  |  |  |
| Trout River (Millvale) |  |  |  |  |  |  |  |  |
| Hunter River |  |  |  |  |  |  |  |  |
| Wheatley River |  |  |  |  |  |  |  |  |
| Black River |  |  |  |  |  |  |  |  |
| Bells Creek (Gurneys River) |  |  |  |  |  |  |  |  |
| Winter River |  |  |  |  |  |  |  |  |
| Morell River | 314,900 | 431,600 | 357,410 | 213,300 | 301,360 | 226,245 | 109,500 | 238,200 |
| Marie River |  |  |  |  |  |  |  |  |
| Midgell River | 50,000 | 60,000 | 50,000 | 50,000 |  | 62,500 | 22,000 | 95,000 |
| St. Peters River | 40,000 | 50,000 | 50,000 | 50,000 |  | 40,000 | 30,000 | 40,000 |
| Schooner Pond |  |  |  |  |  |  |  |  |
| McAskill Creek (Goose River) |  |  |  |  |  |  |  |  |
| Naufrage River |  |  |  |  |  |  |  | 25,000 |
| Cross Creek |  |  |  |  |  |  |  |  |
| North Lake Creek |  |  |  |  |  |  |  |  |
| Bakers River^e^ |  |  |  |  |  |  |  |  |
| Black Pond Creek |  |  |  |  |  |  |  |  |
| Souris River |  |  |  |  |  |  |  |  |
| Rollo Bay |  |  |  |  |  |  |  |  |
| Fortune River |  |  |  |  |  |  |  |  |
| Cardigan River |  |  |  |  |  |  |  |  |
| Brudenell River |  |  |  |  |  |  |  |  |
| Montague River |  |  |  |  |  |  |  |  |
| Valleyfield River |  |  |  |  |  |  |  |  |
| Sturgeon River |  |  |  |  |  |  |  |  |
| Murray River |  |  |  |  |  |  |  |  |
| Fox River |  |  |  |  |  |  |  |  |
| Belle River |  |  |  |  |  |  |  |  |
| Vernon River |  |  |  |  |  |  |  |  |
| Forbes Creek (southeast branch of Fullertons ) |  |  |  |  |  |  |  |  |
| Johnstons River |  |  |  |  |  |  |  |  |
| Glenfinnan River (Sherrys Creek) |  |  |  |  |  |  |  |  |
| Head of Hillsborough River |  |  |  |  |  |  |  |  |
| Hillsborough (East) River, unspecified location |  |  |  |  |  |  |  | 15,000 |
| North River |  |  |  |  |  |  |  |  |
| Clyde River |  |  |  |  |  |  |  |  |
| West River |  |  |  |  |  |  |  |  |
| Desable River |  |  |  |  |  |  |  |  |
| Westmoreland River |  |  |  |  |  |  |  |  |
| Tryon River |  |  |  |  |  |  |  |  |
| Dunk River |  |  |  |  |  |  |  |  |
| Wilmot River |  |  |  |  |  |  |  |  |
| Miminigash River |  |  |  |  |  |  |  |  |
| Skinners Pond |  |  |  |  |  |  |  |  |
| Nail Pond |  |  |  |  |  |  |  |  |
| Curtisdale River^f^ |  |  |  |  |  |  |  |  |
| Inspector River |  |  |  |  |  |  |  |  |
| Mores River^g^ |  |  |  |  |  |  |  |  |
| Trout Newbarton |  |  |  |  |  |  |  |  |
| Breakdown by river not reported |  |  |  |  |  |  |  |  |
| Total number released | 404,900 | 541,600 | 457,410 | 313,300 | 301,360 | 328,745 | 161,500 | 413,200 |
| Total number released, annual sums only | 404,900 | 541,600 | 457,410 | 313,300 | 301,360 | 328,745 | 161,500 | 413,200 |

Table S1 continued

| River name Year | 1953 | 1954 | 1955 | 1956 | 1957 | 1958 | 1959 | 1960 |
| --- | --- | --- | --- | --- | --- | --- | --- | --- |
| Broodstock origin^a,b^ |  |  |  |  |  |  |  |  |
| Run timing of broodstock |  |  |  |  |  |  |  |  |
| Young salmon were reared in semi-natural ponds | None | None | None | None | None | None | None | None |
| Stage | Fry |  |  | Advanced fry |  |  |  |  |
| Tignish River |  |  |  |  |  |  |  |  |
| Mill River (Cains+Carruthers Brooks) |  |  |  |  |  |  |  |  |
| Trout River (Coleman) |  |  |  |  |  |  |  |  |
| Trout River (Tyne Valley) |  |  |  |  |  |  |  |  |
| Indian River |  |  |  |  |  |  |  |  |
| Hy Brook, Morrisons Pond |  |  |  |  |  |  |  |  |
| Trout River (Millvale) |  |  |  |  |  |  |  |  |
| Hunter River |  |  |  |  |  |  |  |  |
| Wheatley River |  |  |  |  |  |  |  |  |
| Black River |  |  |  |  |  |  |  |  |
| Bells Creek (Gurneys River) |  |  |  |  |  |  |  |  |
| Winter River |  |  |  |  |  |  |  |  |
| Morell River | 300,000 | 229,900 | 12,360 | 267,800 | 218,000 | 36,520 | 29,800 | 25,000 |
| Marie River |  |  |  |  |  |  |  |  |
| Midgell River | 110,000 | 105,000 |  | 100,000 | 86,000 | 40,000 |  | 21,200 |
| St. Peters River | 35,000 | 45,000 |  | 30,000 | 40,000 |  |  |  |
| Schooner Pond |  |  |  |  |  |  |  |  |
| McAskill Creek (Goose River) |  |  |  |  |  |  |  |  |
| Naufrage River | 30,000 |  |  | 20,000 | 30,000 |  |  |  |
| Cross Creek |  |  |  |  |  |  |  |  |
| North Lake Creek |  |  |  |  |  |  |  |  |
| Bakers River^e^ |  |  |  |  |  |  |  |  |
| Black Pond Creek |  |  |  |  |  |  |  |  |
| Souris River |  |  |  |  |  |  |  |  |
| Rollo Bay |  |  |  |  |  |  |  |  |
| Fortune River |  |  |  |  |  |  |  |  |
| Cardigan River |  |  |  |  |  |  |  |  |
| Brudenell River |  |  |  |  |  |  |  |  |
| Montague River |  |  |  |  |  |  |  |  |
| Valleyfield River |  |  |  |  |  |  |  |  |
| Sturgeon River |  |  |  |  |  |  |  |  |
| Murray River |  |  |  |  |  |  |  |  |
| Fox River |  |  |  |  |  |  |  |  |
| Belle River |  |  |  |  |  |  |  |  |
| Vernon River |  |  |  |  |  |  |  |  |
| Forbes Creek (southeast branch of Fullertons ) |  |  |  |  |  |  |  |  |
| Johnstons River |  |  |  |  |  |  |  |  |
| Glenfinnan River (Sherrys Creek) |  |  |  |  |  |  |  |  |
| Head of Hillsborough River |  |  |  |  |  |  |  |  |
| Hillsborough (East) River, unspecified location | 20,000 | 15,000 |  | 20,000 |  |  |  |  |
| North River |  |  |  |  |  |  |  |  |
| Clyde River |  |  |  |  |  |  |  |  |
| West River |  |  |  |  |  |  |  |  |
| Desable River |  |  |  |  |  |  |  |  |
| Westmoreland River |  |  |  |  |  |  |  |  |
| Tryon River |  |  |  |  |  |  |  |  |
| Dunk River |  |  |  |  |  | 21,000 |  |  |
| Wilmot River |  |  |  |  |  |  |  |  |
| Miminigash River |  |  |  |  |  |  |  |  |
| Skinners Pond |  |  |  |  |  |  |  |  |
| Nail Pond |  |  |  |  |  |  |  |  |
| Curtisdale River^f^ |  |  |  |  |  |  |  |  |
| Inspector River |  |  |  |  |  |  |  |  |
| Mores River^g^ |  |  |  |  |  |  |  |  |
| Trout Newbarton |  |  |  |  |  |  |  |  |
| Breakdown by river not reported |  |  |  |  |  |  |  |  |
| Total number released | 495,000 | 394,900 | 12,360 | 437,800 | 374,000 | 97,520 | 29,800 | 46,200 |
| Total number released, annual sums only | 495,000 | 394,900 | 12,360 | 437,800 | 374,000 | 97,520 | 29,800 | 46,200 |

Table S1 continued

| River name Year | 1961 | 1962 | 1962 | 1962 | 1963 | 1963 | 1963 | 1964 |
| --- | --- | --- | --- | --- | --- | --- | --- | --- |
| Broodstock origin^a,b^ |  |  |  |  |  |  |  |  |
| Run timing of broodstock |  |  |  |  |  |  |  |  |
| Young salmon were reared in semi-natural ponds |  | None | None | None | None | None | None | None |
| Stage |  | 0+ parr | 1+ parr | Total | 0+ parr | 1+ parr | Total | 0+ parr |
| Tignish River |  | 24,000 |  | 24,000 | 24,000 |  | 24,000 |  |
| Mill River (Cains+Carruthers Brooks) |  |  |  |  |  |  |  |  |
| Trout River (Coleman) |  |  |  |  |  |  |  |  |
| Trout River (Tyne Valley) |  |  |  |  |  |  |  |  |
| Indian River |  |  |  |  |  |  |  |  |
| Hy Brook, Morrisons Pond |  |  |  |  |  |  |  |  |
| Trout River (Millvale) |  |  |  |  |  |  |  |  |
| Hunter River |  |  |  |  |  |  |  |  |
| Wheatley River |  |  |  |  |  |  |  |  |
| Black River |  |  |  |  |  |  |  |  |
| Bells Creek (Gurneys River) |  |  |  |  |  |  |  |  |
| Winter River |  |  |  |  |  |  |  |  |
| Morell River |  | 48,000 | 5,950 | 53,950 | 54,000 | 10,000 | 64,000 | 12,000 |
| Marie River |  |  |  |  |  |  |  |  |
| Midgell River |  | 50,400 |  | 50,400 | 54,000 | 4,000 | 58,000 | 7,200 |
| St. Peters River |  |  |  |  |  |  |  |  |
| Schooner Pond |  |  |  |  |  |  |  |  |
| McAskill Creek (Goose River) |  |  |  |  |  |  |  |  |
| Naufrage River |  | 24,000 |  | 24,000 |  |  |  |  |
| Cross Creek |  |  |  |  |  |  |  |  |
| North Lake Creek |  |  |  |  | 20,000 |  | 20,000 | 6,500 |
| Bakers River^e^ |  |  |  |  |  |  |  |  |
| Black Pond Creek |  |  |  |  |  |  |  |  |
| Souris River |  |  |  |  |  |  |  |  |
| Rollo Bay |  |  |  |  |  |  |  |  |
| Fortune River |  | 16,000 |  | 16,000 | 20,000 |  | 20,000 | 6,500 |
| Cardigan River |  |  | 5,162 | 5,162 |  |  |  |  |
| Brudenell River |  |  |  |  |  |  |  |  |
| Montague River |  |  |  |  |  |  |  |  |
| Valleyfield River |  |  |  |  |  |  |  |  |
| Sturgeon River |  |  |  |  |  |  |  |  |
| Murray River |  |  |  |  |  |  |  |  |
| Fox River |  |  |  |  |  |  |  |  |
| Belle River |  |  |  |  |  |  |  |  |
| Vernon River |  |  |  |  |  |  |  |  |
| Forbes Creek (southeast branch of Fullertons ) |  |  |  |  |  |  |  |  |
| Johnstons River |  |  |  |  |  |  |  |  |
| Glenfinnan River (Sherrys Creek) |  |  |  |  |  |  |  |  |
| Head of Hillsborough River |  |  |  |  |  |  |  |  |
| Hillsborough (East) River, unspecified location |  |  |  |  |  |  |  |  |
| North River |  |  |  |  |  |  |  | 10,000 |
| Clyde River |  |  |  |  |  |  |  |  |
| West River |  |  |  |  |  |  |  |  |
| Desable River |  |  |  |  |  |  |  |  |
| Westmoreland River |  |  |  |  |  |  |  |  |
| Tryon River |  |  |  |  |  |  |  |  |
| Dunk River |  |  |  |  |  |  |  |  |
| Wilmot River |  |  |  |  |  |  |  |  |
| Miminigash River |  |  |  |  |  |  |  |  |
| Skinners Pond |  |  |  |  |  |  |  |  |
| Nail Pond |  |  |  |  |  |  |  |  |
| Curtisdale River^f^ |  |  |  |  |  |  |  |  |
| Inspector River |  |  |  |  |  |  |  |  |
| Mores River^g^ |  |  |  |  |  |  |  |  |
| Trout Newbarton |  |  |  |  |  |  |  |  |
| Breakdown by river not reported |  |  |  |  |  |  |  |  |
| Total number released |  | 162,400 | 11,112 | 173,512 | 172,000 | 14,000 | 186,000 | 42,200 |
| Total number released, annual sums only |  |  |  | 173,512 |  |  | 186,000 | 42,200 |

Table S1 continued

| River name Year | 1965 | 1966 | 1967 | 1968 | 1969 | 1970 | 1971 | 1972 |
| --- | --- | --- | --- | --- | --- | --- | --- | --- |
| Broodstock origin^a,b^ |  |  |  |  |  |  |  |  |
| Run timing of broodstock |  |  |  |  |  |  |  |  |
| Young salmon were reared in semi-natural ponds |  | None | None |  |  |  |  |  |
| Stage |  | 0+ parr | 1+ parr |  |  |  |  |  |
| Tignish River |  |  |  |  |  |  |  |  |
| Mill River (Cains+Carruthers Brooks) |  |  |  |  |  |  |  |  |
| Trout River (Coleman) |  |  |  |  |  |  |  |  |
| Trout River (Tyne Valley) |  |  |  |  |  |  |  |  |
| Indian River |  |  |  |  |  |  |  |  |
| Hy Brook, Morrisons Pond |  |  |  |  |  |  |  |  |
| Trout River (Millvale) |  |  |  |  |  |  |  |  |
| Hunter River |  |  |  |  |  |  |  |  |
| Wheatley River |  |  |  |  |  |  |  |  |
| Black River |  |  |  |  |  |  |  |  |
| Bells Creek (Gurneys River) |  |  |  |  |  |  |  |  |
| Winter River |  |  |  |  |  |  |  |  |
| Morell River |  | 1,575 | 19,370 |  |  |  |  |  |
| Marie River |  |  |  |  |  |  |  |  |
| Midgell River |  |  |  |  |  |  |  |  |
| St. Peters River |  |  |  |  |  |  |  |  |
| Schooner Pond |  |  |  |  |  |  |  |  |
| McAskill Creek (Goose River) |  |  |  |  |  |  |  |  |
| Naufrage River |  |  |  |  |  |  |  |  |
| Cross Creek |  |  |  |  |  |  |  |  |
| North Lake Creek |  |  |  |  |  |  |  |  |
| Bakers River^e^ |  |  |  |  |  |  |  |  |
| Black Pond Creek |  |  |  |  |  |  |  |  |
| Souris River |  |  |  |  |  |  |  |  |
| Rollo Bay |  |  |  |  |  |  |  |  |
| Fortune River |  |  |  |  |  |  |  |  |
| Cardigan River |  |  |  |  |  |  |  |  |
| Brudenell River |  |  |  |  |  |  |  |  |
| Montague River |  |  |  |  |  |  |  |  |
| Valleyfield River |  |  |  |  |  |  |  |  |
| Sturgeon River |  |  |  |  |  |  |  |  |
| Murray River |  |  |  |  |  |  |  |  |
| Fox River |  |  |  |  |  |  |  |  |
| Belle River |  |  |  |  |  |  |  |  |
| Vernon River |  |  |  |  |  |  |  |  |
| Forbes Creek (southeast branch of Fullertons ) |  |  |  |  |  |  |  |  |
| Johnstons River |  |  |  |  |  |  |  |  |
| Glenfinnan River (Sherrys Creek) |  |  |  |  |  |  |  |  |
| Head of Hillsborough River |  |  |  |  |  |  |  |  |
| Hillsborough (East) River, unspecified location |  |  |  |  |  |  |  |  |
| North River |  |  |  |  |  |  |  |  |
| Clyde River |  |  |  |  |  |  |  |  |
| West River |  |  |  |  |  |  |  |  |
| Desable River |  |  |  |  |  |  |  |  |
| Westmoreland River |  |  |  |  |  |  |  |  |
| Tryon River |  |  |  |  |  |  |  |  |
| Dunk River |  |  |  |  |  |  |  |  |
| Wilmot River |  |  |  |  |  |  |  |  |
| Miminigash River |  |  |  |  |  |  |  |  |
| Skinners Pond |  |  |  |  |  |  |  |  |
| Nail Pond |  |  |  |  |  |  |  |  |
| Curtisdale River^f^ |  |  |  |  |  |  |  |  |
| Inspector River |  |  |  |  |  |  |  |  |
| Mores River^g^ |  |  |  |  |  |  |  |  |
| Trout Newbarton |  |  |  |  |  |  |  |  |
| Breakdown by river not reported |  |  |  |  |  |  |  |  |
| Total number released |  | 1,575 | 19,370 |  |  |  |  |  |
| Total number released, annual sums only |  | 1,575 | 19,370 |  |  |  |  |  |

| River name Year | 1973 | 1974 | 1975 | 1976 | 1977 | 1978 | 1979 | 1980 |
| --- | --- | --- | --- | --- | --- | --- | --- | --- |
| Broodstock origin^a,b^ |  |  | RR-NB |  |  | NWMR-NB | Most NWMR-NB, some RR-NB |  |
| Run timing of broodstock |  |  |  |  |  |  |  |  |
| Young salmon were reared in semi-natural ponds |  |  | None |  |  | None | None |  |
| Stage |  |  | 2+ smolts |  |  | Fall fingerling | 0+ parr |  |
| Tignish River |  |  |  |  |  |  |  |  |
| Mill River (Cains+Carruthers Brooks) |  |  |  |  |  |  |  |  |
| Trout River (Coleman) |  |  |  |  |  |  |  |  |
| Trout River (Tyne Valley) |  |  |  |  |  |  |  |  |
| Indian River |  |  |  |  |  |  |  |  |
| Hy Brook, Morrisons Pond |  |  |  |  |  |  |  |  |
| Trout River (Millvale) |  |  |  |  |  |  |  |  |
| Hunter River |  |  |  |  |  |  |  |  |
| Wheatley River |  |  |  |  |  |  |  |  |
| Black River |  |  |  |  |  |  |  |  |
| Bells Creek (Gurneys River) |  |  |  |  |  |  |  |  |
| Winter River |  |  |  |  |  |  |  |  |
| Morell River |  |  | 8,873 |  |  | 14,943 | 32,693 |  |
| Marie River |  |  |  |  |  |  |  |  |
| Midgell River |  |  |  |  |  |  |  |  |
| St. Peters River |  |  |  |  |  |  |  |  |
| Schooner Pond |  |  |  |  |  |  |  |  |
| McAskill Creek (Goose River) |  |  |  |  |  |  |  |  |
| Naufrage River |  |  |  |  |  |  |  |  |
| Cross Creek |  |  |  |  |  |  |  |  |
| North Lake Creek |  |  |  |  |  |  |  |  |
| Bakers River^e^ |  |  |  |  |  |  |  |  |
| Black Pond Creek |  |  |  |  |  |  |  |  |
| Souris River |  |  |  |  |  |  |  |  |
| Rollo Bay |  |  |  |  |  |  |  |  |
| Fortune River |  |  |  |  |  |  |  |  |
| Cardigan River |  |  |  |  |  |  |  |  |
| Brudenell River |  |  |  |  |  |  |  |  |
| Montague River |  |  |  |  |  |  |  |  |
| Valleyfield River |  |  |  |  |  |  |  |  |
| Sturgeon River |  |  |  |  |  |  |  |  |
| Murray River |  |  |  |  |  |  |  |  |
| Fox River |  |  |  |  |  |  |  |  |
| Belle River |  |  |  |  |  |  |  |  |
| Vernon River |  |  |  |  |  |  |  |  |
| Forbes Creek (southeast branch of Fullertons ) |  |  |  |  |  |  |  |  |
| Johnstons River |  |  |  |  |  |  |  |  |
| Glenfinnan River (Sherrys Creek) |  |  |  |  |  |  |  |  |
| Head of Hillsborough River |  |  |  |  |  |  |  |  |
| Hillsborough (East) River, unspecified location |  |  |  |  |  |  |  |  |
| North River |  |  |  |  |  |  |  |  |
| Clyde River |  |  |  |  |  |  |  |  |
| West River |  |  |  |  |  |  |  |  |
| Desable River |  |  |  |  |  |  |  |  |
| Westmoreland River |  |  |  |  |  |  |  |  |
| Tryon River |  |  |  |  |  |  |  |  |
| Dunk River |  |  |  |  |  |  |  |  |
| Wilmot River |  |  |  |  |  |  |  |  |
| Miminigash River |  |  |  |  |  |  |  |  |
| Skinners Pond |  |  |  |  |  |  |  |  |
| Nail Pond |  |  |  |  |  |  |  |  |
| Curtisdale River^f^ |  |  |  |  |  |  |  |  |
| Inspector River |  |  |  |  |  |  |  |  |
| Mores River^g^ |  |  |  |  |  |  |  |  |
| Trout Newbarton |  |  |  |  |  |  |  |  |
| Breakdown by river not reported |  |  |  |  |  |  |  |  |
| Total number released |  |  | 8,873 |  |  | 14,943 | 32,693 |  |
| Total number released, annual sums only |  |  | 8,873 |  |  | 14,943 | 32,693 |  |

Table S1 continued

Table S1 continued

| River name Year | 1981 | 1982 | 1982 | 1982 | 1983 | 1984 | 1985 | 1985 |
| --- | --- | --- | --- | --- | --- | --- | --- | --- |
| Broodstock origin^a,b^ | NWMR-NB | MR-NB | NWMR-NB | NWMR-NB | MR-NB |  | Probably MR-NB | MR-NB |
| Run timing of broodstock |  | Early | Early | Early | Early |  | early and late | early and late |
| Young salmon were reared in semi-natural ponds | None | None | None | None | None |  | Some | All |
| Stage | 2+ smolts | 0+ parr | 2+ smolts | Total | 0+ parr |  | 2+ smolts | 2+ smolts |
| Tignish River |  |  |  |  |  |  |  |  |
| Mill River (Cains+Carruthers Brooks) |  |  |  |  |  |  | 2,342 |  |
| Trout River (Coleman) |  |  |  |  |  |  |  |  |
| Trout River (Tyne Valley) |  |  |  |  |  |  |  |  |
| Indian River |  |  |  |  |  |  |  |  |
| Hy Brook, Morrisons Pond |  |  |  |  |  |  |  |  |
| Trout River (Millvale) |  |  |  |  |  |  |  |  |
| Hunter River |  |  |  |  |  |  |  |  |
| Wheatley River |  |  |  |  |  |  |  |  |
| Black River |  |  |  |  |  |  |  |  |
| Bells Creek (Gurneys River) |  |  |  |  |  |  |  |  |
| Winter River |  |  |  |  |  |  |  |  |
| Morell River | 691 | 34,764 | 3,645 | 38,409 | 9,000 |  |  | 10,997 |
| Marie River |  |  |  |  |  |  |  |  |
| Midgell River |  |  |  |  |  |  |  |  |
| St. Peters River |  |  |  |  |  |  |  |  |
| Schooner Pond |  |  |  |  |  |  |  |  |
| McAskill Creek (Goose River) |  |  |  |  |  |  |  |  |
| Naufrage River |  |  |  |  |  |  |  |  |
| Cross Creek |  |  |  |  |  |  |  |  |
| North Lake Creek |  |  |  |  |  |  |  |  |
| Bakers River^e^ |  |  |  |  |  |  |  |  |
| Black Pond Creek |  |  |  |  |  |  |  |  |
| Souris River |  |  |  |  |  |  |  |  |
| Rollo Bay |  |  |  |  |  |  |  |  |
| Fortune River |  |  |  |  |  |  |  |  |
| Cardigan River |  |  |  |  |  |  |  |  |
| Brudenell River |  |  |  |  |  |  |  |  |
| Montague River |  |  |  |  |  |  |  |  |
| Valleyfield River |  |  |  |  |  |  |  |  |
| Sturgeon River |  |  |  |  |  |  |  |  |
| Murray River |  |  |  |  |  |  |  |  |
| Fox River |  |  |  |  |  |  |  |  |
| Belle River |  |  |  |  |  |  |  |  |
| Vernon River |  |  |  |  |  |  |  |  |
| Forbes Creek (southeast branch of Fullertons ) |  |  |  |  |  |  |  |  |
| Johnstons River |  |  |  |  |  |  |  |  |
| Glenfinnan River (Sherrys Creek) |  |  |  |  |  |  |  |  |
| Head of Hillsborough River |  |  |  |  |  |  |  |  |
| Hillsborough (East) River, unspecified location |  |  |  |  |  |  |  |  |
| North River |  |  |  |  |  |  |  |  |
| Clyde River |  |  |  |  |  |  |  |  |
| West River |  |  |  |  |  |  |  |  |
| Desable River |  |  |  |  |  |  |  |  |
| Westmoreland River |  |  |  |  |  |  |  |  |
| Tryon River |  |  |  |  |  |  |  |  |
| Dunk River |  |  |  |  |  |  |  |  |
| Wilmot River |  |  |  |  |  |  |  |  |
| Miminigash River |  |  |  |  |  |  |  |  |
| Skinners Pond |  |  |  |  |  |  |  |  |
| Nail Pond |  |  |  |  |  |  |  |  |
| Curtisdale River^f^ |  |  |  |  |  |  |  |  |
| Inspector River |  |  |  |  |  |  |  |  |
| Mores River^g^ |  |  |  |  |  |  |  |  |
| Trout Newbarton |  |  |  |  |  |  |  |  |
| Breakdown by river not reported |  |  |  |  |  |  |  |  |
| Total number released | 691 | 34,764 | 3,645 | 38,409 | 9,000 |  | 2,342 | 10,997 |
| Total number released, annual sums only | 691 |  |  | 38,409 | 9,000 |  |  |  |

Table S1 continued

| River name Year | 1985 | 1985 | 1985 | 1986 | 1986 | 1986 | 1986 | 1986 |
| --- | --- | --- | --- | --- | --- | --- | --- | --- |
| Broodstock origin^a,b^ | MR-NB | MR-NB |  | Probably NWMR-NB | Probably NWMR-NB | Probably NWMR-NB | NWMR-NB | NWMR-NB |
| Run timing of broodstock | early and late | early and late | early and late | Probably early | Probably early | Probably early | Early | Early |
| Young salmon were reared in semi-natural ponds | None | Some |  | All | All | All | All | Most |
| Stage | 2+ smolts | Total, Morell | Total, all rivers | 2+ parr | 2+ smolts | Total, non-Morell | 2+ parr | 2+ smolts |
| Tignish River |  |  |  |  |  |  |  |  |
| Mill River (Cains+Carruthers Brooks) |  |  | 2,342 | 580 | 2,417 | 2,997 |  |  |
| Trout River (Coleman) |  |  |  |  |  |  |  |  |
| Trout River (Tyne Valley) |  |  |  |  |  |  |  |  |
| Indian River |  |  |  |  |  |  |  |  |
| Hy Brook, Morrisons Pond |  |  |  |  |  |  |  |  |
| Trout River (Millvale) |  |  |  |  |  |  |  |  |
| Hunter River |  |  |  |  |  |  |  |  |
| Wheatley River |  |  |  |  |  |  |  |  |
| Black River |  |  |  |  |  |  |  |  |
| Bells Creek (Gurneys River) |  |  |  |  |  |  |  |  |
| Winter River |  |  |  |  |  |  |  |  |
| Morell River | 10,428 | 21,425 | 21,425 |  |  |  | 570 | 14,058 |
| Marie River |  |  |  |  |  |  |  |  |
| Midgell River |  |  |  |  |  |  |  |  |
| St. Peters River |  |  |  |  |  |  |  |  |
| Schooner Pond |  |  |  |  |  |  |  |  |
| McAskill Creek (Goose River) |  |  |  |  |  |  |  |  |
| Naufrage River |  |  |  |  |  |  |  |  |
| Cross Creek |  |  |  |  |  |  |  |  |
| North Lake Creek |  |  |  |  |  |  |  |  |
| Bakers River^e^ |  |  |  |  |  |  |  |  |
| Black Pond Creek |  |  |  |  |  |  |  |  |
| Souris River |  |  |  |  |  |  |  |  |
| Rollo Bay |  |  |  |  |  |  |  |  |
| Fortune River |  |  |  |  |  |  |  |  |
| Cardigan River |  |  |  |  |  |  |  |  |
| Brudenell River |  |  |  |  |  |  |  |  |
| Montague River |  |  |  |  |  |  |  |  |
| Valleyfield River |  |  |  |  |  |  |  |  |
| Sturgeon River |  |  |  |  |  |  |  |  |
| Murray River |  |  |  |  |  |  |  |  |
| Fox River |  |  |  |  |  |  |  |  |
| Belle River |  |  |  |  |  |  |  |  |
| Vernon River |  |  |  |  |  |  |  |  |
| Forbes Creek (southeast branch of Fullertons ) |  |  |  |  |  |  |  |  |
| Johnstons River |  |  |  |  |  |  |  |  |
| Glenfinnan River (Sherrys Creek) |  |  |  |  |  |  |  |  |
| Head of Hillsborough River |  |  |  |  |  |  |  |  |
| Hillsborough (East) River, unspecified location |  |  |  |  |  |  |  |  |
| North River |  |  |  |  |  |  |  |  |
| Clyde River |  |  |  |  |  |  |  |  |
| West River |  |  |  |  |  |  |  |  |
| Desable River |  |  |  |  |  |  |  |  |
| Westmoreland River |  |  |  |  |  |  |  |  |
| Tryon River |  |  |  |  |  |  |  |  |
| Dunk River |  |  |  |  |  |  |  |  |
| Wilmot River |  |  |  |  |  |  |  |  |
| Miminigash River |  |  |  |  |  |  |  |  |
| Skinners Pond |  |  |  |  |  |  |  |  |
| Nail Pond |  |  |  |  |  |  |  |  |
| Curtisdale River^f^ |  |  |  |  |  |  |  |  |
| Inspector River |  |  |  |  |  |  |  |  |
| Mores River^g^ |  |  |  |  |  |  |  |  |
| Trout Newbarton |  |  |  |  |  |  |  |  |
| Breakdown by river not reported |  |  |  |  |  |  |  |  |
| Total number released | 10,428 | 21,425 | 23,767 | 580 | 2,417 | 2,997 | 570 | 14,058 |
| Total number released, annual sums only |  |  | 23,767 |  |  |  |  |  |

Table S1 continued

| River name Year | 1986 | 1986 | 1987 | 1987 | 1987 | 1987 | 1987 | 1987 |
| --- | --- | --- | --- | --- | --- | --- | --- | --- |
| Broodstock origin^a,b^ | NWMR-NB |  | Probably NWMR-NB | Probably NWMR-NB | Probably NWMR-NB | NWMR-NB | NWMR-NB | NWMR-NB |
| Run timing of broodstock | Early |  | Probably early | Probably early | Probably early | Early | Early | Early |
| Young salmon were reared in semi-natural ponds | Most |  | All | All | All | All | Most | Most |
| Stage | Total, Morell | Total, all rivers | 2+ parr | 2+ smolts | Total, non-Morell | 2+ parr | 2+ smolts | Total, Morell |
| Tignish River |  |  |  |  |  |  |  |  |
| Mill River (Cains+Carruthers Brooks) |  | 2,997 | 595 | 2,555 | 3,150 |  |  |  |
| Trout River (Coleman) |  |  |  |  |  |  |  |  |
| Trout River (Tyne Valley) |  |  |  |  |  |  |  |  |
| Indian River |  |  |  |  |  |  |  |  |
| Hy Brook, Morrisons Pond |  |  |  |  |  |  |  |  |
| Trout River (Millvale) |  |  |  |  |  |  |  |  |
| Hunter River |  |  |  |  |  |  |  |  |
| Wheatley River |  |  |  |  |  |  |  |  |
| Black River |  |  |  |  |  |  |  |  |
| Bells Creek (Gurneys River) |  |  |  |  |  |  |  |  |
| Winter River |  |  |  |  |  |  |  |  |
| Morell River | 14,628 | 14,628 |  |  |  | 3,479 | 25,305 | 28,784 |
| Marie River |  |  |  |  |  |  |  |  |
| Midgell River |  |  |  |  |  |  |  |  |
| St. Peters River |  |  |  |  |  |  |  |  |
| Schooner Pond |  |  |  |  |  |  |  |  |
| McAskill Creek (Goose River) |  |  |  |  |  |  |  |  |
| Naufrage River |  |  |  |  |  |  |  |  |
| Cross Creek |  |  |  |  |  |  |  |  |
| North Lake Creek |  |  |  |  |  |  |  |  |
| Bakers River^e^ |  |  |  |  |  |  |  |  |
| Black Pond Creek |  |  |  |  |  |  |  |  |
| Souris River |  |  |  |  |  |  |  |  |
| Rollo Bay |  |  |  |  |  |  |  |  |
| Fortune River |  |  |  |  |  |  |  |  |
| Cardigan River |  |  |  |  |  |  |  |  |
| Brudenell River |  |  |  |  |  |  |  |  |
| Montague River |  |  |  |  |  |  |  |  |
| Valleyfield River |  |  |  |  |  |  |  |  |
| Sturgeon River |  |  |  |  |  |  |  |  |
| Murray River |  |  |  |  |  |  |  |  |
| Fox River |  |  |  |  |  |  |  |  |
| Belle River |  |  |  |  |  |  |  |  |
| Vernon River |  |  |  |  |  |  |  |  |
| Forbes Creek (southeast branch of Fullertons ) |  |  |  |  |  |  |  |  |
| Johnstons River |  |  |  |  |  |  |  |  |
| Glenfinnan River (Sherrys Creek) |  |  |  |  |  |  |  |  |
| Head of Hillsborough River |  |  |  |  |  |  |  |  |
| Hillsborough (East) River, unspecified location |  |  |  |  |  |  |  |  |
| North River |  |  |  |  |  |  |  |  |
| Clyde River |  |  |  |  |  |  |  |  |
| West River |  |  |  |  |  |  |  |  |
| Desable River |  |  |  |  |  |  |  |  |
| Westmoreland River |  |  |  |  |  |  |  |  |
| Tryon River |  |  |  |  |  |  |  |  |
| Dunk River |  |  |  |  |  |  |  |  |
| Wilmot River |  |  |  |  |  |  |  |  |
| Miminigash River |  |  |  |  |  |  |  |  |
| Skinners Pond |  |  |  |  |  |  |  |  |
| Nail Pond |  |  |  |  |  |  |  |  |
| Curtisdale River^f^ |  |  |  |  |  |  |  |  |
| Inspector River |  |  |  |  |  |  |  |  |
| Mores River^g^ |  |  |  |  |  |  |  |  |
| Trout Newbarton |  |  |  |  |  |  |  |  |
| Breakdown by river not reported |  |  |  |  |  |  |  |  |
| Total number released | 14,628 | 17,625 | 595 | 2,555 | 3,150 | 3,479 | 25,305 | 28,784 |
| Total number released, annual sums only |  | 17,625 |  |  |  |  |  |  |

Table S1 continued

| River name Year | 1987 | 1988 | 1988 | 1988 | 1988 | 1988 | 1988 | 1988 |
| --- | --- | --- | --- | --- | --- | --- | --- | --- |
| Broodstock origin^a,b^ |  | Probably MR | Probably MR | Probably MR | Probably MR | MR | MR | MR |
| Run timing of broodstock |  | Probably early and late | Probably early and late | Probably early and late | Probably early and late | Mixed early and late | Mixed early and late | Mixed early and late |
| Young salmon were reared in semi-natural ponds |  | All | None | All | None | All | None | All |
| Stage | Total, all rivers | 2+ parr | 1+ smolts | 2+ smolts | Total, non-Morell | 2+ parr | 1+ smolts | 2+ smolts |
| Tignish River |  |  |  |  |  |  |  |  |
| Mill River (Cains+Carruthers Brooks) | 3,150 | 349 |  | 3,079 | 3,428 |  |  |  |
| Trout River (Coleman) |  |  |  |  |  |  |  |  |
| Trout River (Tyne Valley) |  |  |  |  |  |  |  |  |
| Indian River |  |  |  |  |  |  |  |  |
| Hy Brook, Morrisons Pond |  |  |  |  |  |  |  |  |
| Trout River (Millvale) |  |  |  |  |  |  |  |  |
| Hunter River |  |  |  |  |  |  |  |  |
| Wheatley River |  |  |  |  |  |  |  |  |
| Black River |  |  |  |  |  |  |  |  |
| Bells Creek (Gurneys River) |  |  |  |  |  |  |  |  |
| Winter River |  |  |  |  |  |  |  |  |
| Morell River | 28,784 |  |  |  |  | 1,208 | 5,907 | 12,982 |
| Marie River |  |  |  |  |  |  |  |  |
| Midgell River |  |  |  |  |  |  |  |  |
| St. Peters River |  |  |  |  |  |  |  |  |
| Schooner Pond |  |  |  |  |  |  |  |  |
| McAskill Creek (Goose River) |  |  |  |  |  |  |  |  |
| Naufrage River |  |  |  |  |  |  |  |  |
| Cross Creek |  |  |  |  |  |  |  |  |
| North Lake Creek |  |  |  |  |  |  |  |  |
| Bakers River^e^ |  |  |  |  |  |  |  |  |
| Black Pond Creek |  |  |  |  |  |  |  |  |
| Souris River |  |  |  |  |  |  |  |  |
| Rollo Bay |  |  |  |  |  |  |  |  |
| Fortune River |  |  |  |  |  |  |  |  |
| Cardigan River |  |  |  |  |  |  |  |  |
| Brudenell River |  |  |  |  |  |  |  |  |
| Montague River |  |  |  |  |  |  |  |  |
| Valleyfield River |  |  |  |  |  |  |  |  |
| Sturgeon River |  |  |  |  |  |  |  |  |
| Murray River |  |  |  |  |  |  |  |  |
| Fox River |  |  |  |  |  |  |  |  |
| Belle River |  |  |  |  |  |  |  |  |
| Vernon River |  |  |  |  |  |  |  |  |
| Forbes Creek (southeast branch of Fullertons ) |  |  |  |  |  |  |  |  |
| Johnstons River |  |  |  |  |  |  |  |  |
| Glenfinnan River (Sherrys Creek) |  |  |  |  |  |  |  |  |
| Head of Hillsborough River |  |  |  |  |  |  |  |  |
| Hillsborough (East) River, unspecified location |  |  |  |  |  |  |  |  |
| North River |  |  |  |  |  |  |  |  |
| Clyde River |  |  |  |  |  |  |  |  |
| West River |  |  | 1,390 |  | 1,390 |  |  |  |
| Desable River |  |  |  |  |  |  |  |  |
| Westmoreland River |  |  |  |  |  |  |  |  |
| Tryon River |  |  |  |  |  |  |  |  |
| Dunk River |  |  |  |  |  |  |  |  |
| Wilmot River |  |  |  |  |  |  |  |  |
| Miminigash River |  |  |  |  |  |  |  |  |
| Skinners Pond |  |  |  |  |  |  |  |  |
| Nail Pond |  |  |  |  |  |  |  |  |
| Curtisdale River^f^ |  |  |  |  |  |  |  |  |
| Inspector River |  |  |  |  |  |  |  |  |
| Mores River^g^ |  |  |  |  |  |  |  |  |
| Trout Newbarton |  |  |  |  |  |  |  |  |
| Breakdown by river not reported |  |  |  |  |  |  |  |  |
| Total number released | 31,934 | 349 | 1,390 | 3,079 | 4,818 | 1,208 | 5,907 | 12,982 |
| Total number released, annual sums only | 31,934 |  |  |  |  |  |  |  |

Table S1 continued

| River name Year | 1988 | 1988 | 1989 | 1989 | 1989 | 1989 | 1989 | 1989 |
| --- | --- | --- | --- | --- | --- | --- | --- | --- |
| Broodstock origin^a,b^ | MR |  | Probably MR HR | Probably MR HR | Probably MR HR | Probably MR HR | Probably MR HR | MR HR |
| Run timing of broodstock | early and late |  |  |  |  |  |  |  |
| Young salmon were reared in semi-natural ponds | Most |  | None | All | None | All | None | All |
| Stage | Total, Morell | Total, all rivers | 1+ parr | 2+ parr | 1+ smolts | 2+ smolts | Total, non-Morell | 2+ parr |
| Tignish River |  |  |  |  |  |  |  |  |
| Mill River (Cains+Carruthers Brooks) |  | 3,428 |  | 74 |  | 2,991 | 3,065 |  |
| Trout River (Coleman) |  |  |  |  |  |  |  |  |
| Trout River (Tyne Valley) |  |  |  |  |  |  |  |  |
| Indian River |  |  |  |  |  |  |  |  |
| Hy Brook, Morrisons Pond |  |  |  |  |  |  |  |  |
| Trout River (Millvale) |  |  |  |  |  |  |  |  |
| Hunter River |  |  |  |  |  |  |  |  |
| Wheatley River |  |  |  |  |  |  |  |  |
| Black River |  |  |  |  |  |  |  |  |
| Bells Creek (Gurneys River) |  |  |  |  |  |  |  |  |
| Winter River |  |  |  |  |  |  |  |  |
| Morell River | 20,097 | 20,097 |  |  |  |  |  | 1,560 |
| Marie River |  |  |  |  |  |  |  |  |
| Midgell River |  |  |  |  |  |  |  |  |
| St. Peters River |  |  |  |  |  |  |  |  |
| Schooner Pond |  |  |  |  |  |  |  |  |
| McAskill Creek (Goose River) |  |  |  |  |  |  |  |  |
| Naufrage River |  |  |  |  |  |  |  |  |
| Cross Creek |  |  |  |  |  |  |  |  |
| North Lake Creek |  |  |  |  |  |  |  |  |
| Bakers River^e^ |  |  |  |  |  |  |  |  |
| Black Pond Creek |  |  |  |  |  |  |  |  |
| Souris River |  |  |  |  |  |  |  |  |
| Rollo Bay |  |  |  |  |  |  |  |  |
| Fortune River |  |  |  |  |  |  |  |  |
| Cardigan River |  |  |  |  |  |  |  |  |
| Brudenell River |  |  |  |  |  |  |  |  |
| Montague River |  |  |  |  |  |  |  |  |
| Valleyfield River |  |  | 2,491 |  | 6,299 |  | 8,790 |  |
| Sturgeon River |  |  |  |  |  |  |  |  |
| Murray River |  |  |  |  |  |  |  |  |
| Fox River |  |  |  |  |  |  |  |  |
| Belle River |  |  |  |  |  |  |  |  |
| Vernon River |  |  |  |  |  |  |  |  |
| Forbes Creek (southeast branch of Fullertons ) |  |  |  |  |  |  |  |  |
| Johnstons River |  |  |  |  |  |  |  |  |
| Glenfinnan River (Sherrys Creek) |  |  |  |  |  |  |  |  |
| Head of Hillsborough River |  |  |  |  |  |  |  |  |
| Hillsborough (East) River, unspecified location |  |  |  |  |  |  |  |  |
| North River |  |  |  |  |  |  |  |  |
| Clyde River |  |  |  |  |  |  |  |  |
| West River |  | 1,390 |  |  |  | 1,324 | 1,324 |  |
| Desable River |  |  |  |  |  |  |  |  |
| Westmoreland River |  |  |  |  |  |  |  |  |
| Tryon River |  |  |  |  |  |  |  |  |
| Dunk River |  |  |  |  |  |  |  |  |
| Wilmot River |  |  |  |  |  |  |  |  |
| Miminigash River |  |  |  |  |  |  |  |  |
| Skinners Pond |  |  |  |  |  |  |  |  |
| Nail Pond |  |  |  |  |  |  |  |  |
| Curtisdale River^f^ |  |  |  |  |  |  |  |  |
| Inspector River |  |  |  |  |  |  |  |  |
| Mores River^g^ |  |  |  |  |  |  |  |  |
| Trout Newbarton |  |  |  |  |  |  |  |  |
| Breakdown by river not reported |  |  |  |  |  |  |  |  |
| Total number released | 20,097 | 24,915 | 2,491 | 74 | 6,299 | 4,315 | 13,179 | 1,560 |
| Total number released, annual sums only |  | 24,915 |  |  |  |  |  |  |

Table S1 continued

| River name Year | 1989 | 1989 | 1989 | 1990 | 1990 | 1990 | 1990 | 1990 |
| --- | --- | --- | --- | --- | --- | --- | --- | --- |
| Broodstock origin^a,b^ | MR HR | MR HR |  | Probably MR HR | Probably MR HR | Probably MR HR | Probably MR HR | Probably MR HR |
| Run timing of broodstock |  |  |  | early and late | early and late | early and late | early and late | early and late |
| Young salmon were reared in semi-natural ponds | All | All |  | None | All | None | All | None |
| Stage | 2+ smolts | Total, Morell | Total, all rivers | 0+ parr | 2+ parr | 1+ smolts | 2+ smolts | Total, non-Morell |
| Tignish River |  |  |  |  |  |  |  |  |
| Mill River (Cains+Carruthers Brooks) |  |  | 3,065 |  | 25 |  | 3,082 | 3,107 |
| Trout River (Coleman) |  |  |  |  |  |  |  |  |
| Trout River (Tyne Valley) |  |  |  |  |  |  |  |  |
| Indian River |  |  |  |  |  |  |  |  |
| Hy Brook, Morrisons Pond |  |  |  |  |  |  |  |  |
| Trout River (Millvale) |  |  |  |  |  |  |  |  |
| Hunter River |  |  |  |  |  |  |  |  |
| Wheatley River |  |  |  |  |  |  |  |  |
| Black River |  |  |  |  |  |  |  |  |
| Bells Creek (Gurneys River) |  |  |  |  |  |  |  |  |
| Winter River |  |  |  |  |  |  |  |  |
| Morell River | 20,650 | 22,210 | 22,210 |  |  |  |  |  |
| Marie River |  |  |  |  |  |  |  |  |
| Midgell River |  |  |  |  |  |  |  |  |
| St. Peters River |  |  |  |  |  |  |  |  |
| Schooner Pond |  |  |  |  |  |  |  |  |
| McAskill Creek (Goose River) |  |  |  |  |  |  |  |  |
| Naufrage River |  |  |  |  |  |  |  |  |
| Cross Creek |  |  |  |  |  |  |  |  |
| North Lake Creek |  |  |  |  |  |  |  |  |
| Bakers River^e^ |  |  |  |  |  |  |  |  |
| Black Pond Creek |  |  |  |  |  |  |  |  |
| Souris River |  |  |  |  |  |  |  |  |
| Rollo Bay |  |  |  |  |  |  |  |  |
| Fortune River |  |  |  |  |  |  |  |  |
| Cardigan River |  |  |  |  |  |  |  |  |
| Brudenell River |  |  |  |  |  |  |  |  |
| Montague River |  |  |  |  |  |  |  |  |
| Valleyfield River |  |  | 8,790 | 89,003 |  | 738 |  | 89,741 |
| Sturgeon River |  |  |  |  |  |  |  |  |
| Murray River |  |  |  |  |  |  |  |  |
| Fox River |  |  |  |  |  |  |  |  |
| Belle River |  |  |  |  |  |  |  |  |
| Vernon River |  |  |  |  |  |  |  |  |
| Forbes Creek (southeast branch of Fullertons ) |  |  |  |  |  |  |  |  |
| Johnstons River |  |  |  |  |  |  |  |  |
| Glenfinnan River (Sherrys Creek) |  |  |  |  |  |  |  |  |
| Head of Hillsborough River |  |  |  |  |  |  |  |  |
| Hillsborough (East) River, unspecified location |  |  |  |  |  |  |  |  |
| North River |  |  |  |  |  |  |  |  |
| Clyde River |  |  |  |  |  |  |  |  |
| West River |  |  | 1,324 |  |  |  |  |  |
| Desable River |  |  |  |  |  |  |  |  |
| Westmoreland River |  |  |  |  |  |  |  |  |
| Tryon River |  |  |  |  |  |  |  |  |
| Dunk River |  |  |  |  |  |  |  |  |
| Wilmot River |  |  |  |  |  |  |  |  |
| Miminigash River |  |  |  |  |  |  |  |  |
| Skinners Pond |  |  |  |  |  |  |  |  |
| Nail Pond |  |  |  |  |  |  |  |  |
| Curtisdale River^f^ |  |  |  |  |  |  |  |  |
| Inspector River |  |  |  |  |  |  |  |  |
| Mores River^g^ |  |  |  |  |  |  |  |  |
| Trout Newbarton |  |  |  |  |  |  |  |  |
| Breakdown by river not reported |  |  |  |  |  |  |  |  |
| Total number released | 20,650 | 22,210 | 35,389 | 89,003 | 25 | 738 | 3,082 | 92,848 |
| Total number released, annual sums only |  |  | 35,389 |  |  |  |  |  |

Table S1 continued

| River name Year | 1990 | 1990 | 1990 | 1990 | 1991 | 1991 | 1991 | 1991 |
| --- | --- | --- | --- | --- | --- | --- | --- | --- |
| Broodstock origin^a,b^ | MR HR | MR HR | MR HR |  | Probably MR HR | Probably MR HR | Probably MR HR | Probably MR HR |
| Run timing of broodstock | early and late | early and late | early and late |  | early and late | early and late | early and late | early and late |
| Young salmon were reared in semi-natural ponds | All | All | All |  | None | All | None | All |
| Stage | 2+ parr | 2+ smolts | Total, Morell | Total, all rivers | 0+ parr | 2+ parr | 1+ smolts | 2+ smolts |
| Tignish River |  |  |  |  |  |  |  |  |
| Mill River (Cains+Carruthers Brooks) |  |  |  | 3,107 |  | 159 |  | 1,873 |
| Trout River (Coleman) |  |  |  |  |  |  |  |  |
| Trout River (Tyne Valley) |  |  |  |  |  |  |  |  |
| Indian River |  |  |  |  |  |  |  |  |
| Hy Brook, Morrisons Pond |  |  |  |  |  |  |  |  |
| Trout River (Millvale) |  |  |  |  |  |  |  |  |
| Hunter River |  |  |  |  |  |  |  |  |
| Wheatley River |  |  |  |  |  |  |  |  |
| Black River |  |  |  |  |  |  |  |  |
| Bells Creek (Gurneys River) |  |  |  |  |  |  |  |  |
| Winter River |  |  |  |  |  |  |  |  |
| Morell River | 1,079 | 58,731 | 59,810 | 59,810 |  |  |  |  |
| Marie River |  |  |  |  |  |  |  |  |
| Midgell River |  |  |  |  |  |  |  |  |
| St. Peters River |  |  |  |  |  |  |  |  |
| Schooner Pond |  |  |  |  |  |  |  |  |
| McAskill Creek (Goose River) |  |  |  |  |  |  |  |  |
| Naufrage River |  |  |  |  |  |  |  |  |
| Cross Creek |  |  |  |  |  |  |  |  |
| North Lake Creek |  |  |  |  |  |  |  |  |
| Bakers River^e^ |  |  |  |  |  |  |  |  |
| Black Pond Creek |  |  |  |  |  |  |  |  |
| Souris River |  |  |  |  |  |  |  |  |
| Rollo Bay |  |  |  |  |  |  |  |  |
| Fortune River |  |  |  |  |  |  |  |  |
| Cardigan River |  |  |  |  |  |  |  |  |
| Brudenell River |  |  |  |  |  |  |  |  |
| Montague River |  |  |  |  |  |  |  |  |
| Valleyfield River |  |  |  | 89,741 | 55,723 |  | 5,259 |  |
| Sturgeon River |  |  |  |  |  |  |  |  |
| Murray River |  |  |  |  |  |  |  |  |
| Fox River |  |  |  |  |  |  |  |  |
| Belle River |  |  |  |  |  |  |  |  |
| Vernon River |  |  |  |  |  |  |  |  |
| Forbes Creek (southeast branch of Fullertons ) |  |  |  |  |  |  |  |  |
| Johnstons River |  |  |  |  |  |  |  |  |
| Glenfinnan River (Sherrys Creek) |  |  |  |  |  |  |  |  |
| Head of Hillsborough River |  |  |  |  |  |  |  |  |
| Hillsborough (East) River, unspecified location |  |  |  |  |  |  |  |  |
| North River |  |  |  |  |  |  |  |  |
| Clyde River |  |  |  |  |  |  |  |  |
| West River |  |  |  |  | 50,750 |  |  |  |
| Desable River |  |  |  |  |  |  |  |  |
| Westmoreland River |  |  |  |  |  |  |  |  |
| Tryon River |  |  |  |  |  |  |  |  |
| Dunk River |  |  |  |  |  |  |  | 2,017 |
| Wilmot River |  |  |  |  |  |  |  |  |
| Miminigash River |  |  |  |  |  |  |  |  |
| Skinners Pond |  |  |  |  |  |  |  |  |
| Nail Pond |  |  |  |  |  |  |  |  |
| Curtisdale River^f^ |  |  |  |  |  |  |  |  |
| Inspector River |  |  |  |  |  |  |  |  |
| Mores River^g^ |  |  |  |  |  |  |  |  |
| Trout Newbarton |  |  |  |  |  |  |  |  |
| Breakdown by river not reported |  |  |  |  |  |  |  |  |
| Total number released | 1,079 | 58,731 | 59,810 | 152,658 | 106,473 | 159 | 5,259 | 3,890 |
| Total number released, annual sums only |  |  |  | 152,658 |  |  |  |  |

Table S1 continued

| River name | 1991 | 1991 | 1991 | 1991 | 1991 | 1992 | 1992 | 1992 |
| --- | --- | --- | --- | --- | --- | --- | --- | --- |
| Broodstock origin^a,b^ | Probably MR HR | MR HR | MR HR | MR HR |  | Probably MR HR | Probably MR HR | Probably MR HR |
| Run timing of broodstock | early and late | early and late | early and late | early and late |  | early and late | early and late | early and late |
| Young salmon were reared in semi-natural ponds | None | All | All | All |  | None | All | All |
| Stage | Total, non-Morell | 2+ parr | 2+ smolts | Total, Morell | Total, all rivers | 0+ parr | 1+ parr | 2+ parr |
| Tignish River |  |  |  |  |  |  |  |  |
| Mill River (Cains+Carruthers Brooks) | 2,032 |  |  |  | 2,032 |  |  | 169 |
| Trout River (Coleman) |  |  |  |  |  |  |  |  |
| Trout River (Tyne Valley) |  |  |  |  |  |  |  |  |
| Indian River |  |  |  |  |  |  |  |  |
| Hy Brook, Morrisons Pond |  |  |  |  |  |  |  |  |
| Trout River (Millvale) |  |  |  |  |  |  |  |  |
| Hunter River |  |  |  |  |  |  |  |  |
| Wheatley River |  |  |  |  |  |  |  |  |
| Black River |  |  |  |  |  |  |  |  |
| Bells Creek (Gurneys River) |  |  |  |  |  |  |  |  |
| Winter River |  |  |  |  |  |  |  |  |
| Morell River |  | 2,053 | 34,443 | 36,496 | 36,496 |  |  |  |
| Marie River |  |  |  |  |  |  |  |  |
| Midgell River |  |  |  |  |  |  |  |  |
| St. Peters River |  |  |  |  |  |  |  |  |
| Schooner Pond |  |  |  |  |  |  |  |  |
| McAskill Creek (Goose River) |  |  |  |  |  |  |  |  |
| Naufrage River |  |  |  |  |  |  |  |  |
| Cross Creek |  |  |  |  |  |  |  |  |
| North Lake Creek |  |  |  |  |  |  |  |  |
| Bakers River^e^ |  |  |  |  |  |  |  |  |
| Black Pond Creek |  |  |  |  |  |  |  |  |
| Souris River |  |  |  |  |  |  |  |  |
| Rollo Bay |  |  |  |  |  |  |  |  |
| Fortune River |  |  |  |  |  |  |  |  |
| Cardigan River |  |  |  |  |  |  |  |  |
| Brudenell River |  |  |  |  |  |  |  |  |
| Montague River |  |  |  |  |  |  |  |  |
| Valleyfield River | 60,982 |  |  |  | 60,982 | 32,494 | 10,014 |  |
| Sturgeon River |  |  |  |  |  |  |  |  |
| Murray River |  |  |  |  |  |  |  |  |
| Fox River |  |  |  |  |  |  |  |  |
| Belle River |  |  |  |  |  |  |  |  |
| Vernon River |  |  |  |  |  |  |  |  |
| Forbes Creek (southeast branch of Fullertons ) |  |  |  |  |  |  |  |  |
| Johnstons River |  |  |  |  |  |  |  |  |
| Glenfinnan River (Sherrys Creek) |  |  |  |  |  |  |  |  |
| Head of Hillsborough River |  |  |  |  |  |  |  |  |
| Hillsborough (East) River, unspecified location |  |  |  |  |  |  |  |  |
| North River |  |  |  |  |  |  |  |  |
| Clyde River |  |  |  |  |  |  |  |  |
| West River | 50,750 |  |  |  | 50,750 |  | 10,173 |  |
| Desable River |  |  |  |  |  |  |  |  |
| Westmoreland River |  |  |  |  |  |  |  |  |
| Tryon River |  |  |  |  |  |  |  |  |
| Dunk River | 2,017 |  |  |  | 2,017 |  |  |  |
| Wilmot River |  |  |  |  |  |  |  |  |
| Miminigash River |  |  |  |  |  |  |  |  |
| Skinners Pond |  |  |  |  |  |  |  |  |
| Nail Pond |  |  |  |  |  |  |  |  |
| Curtisdale River^f^ |  |  |  |  |  |  |  |  |
| Inspector River |  |  |  |  |  |  |  |  |
| Mores River^g^ |  |  |  |  |  |  |  |  |
| Trout Newbarton |  |  |  |  |  |  |  |  |
| Breakdown by river not reported |  |  |  |  |  |  |  |  |
| Total number released | 115,781 | 2,053 | 34,443 | 36,496 | 152,277 | 32,494 | 20,187 | 169 |
| Total number released, annual sums only |  |  |  |  | 152,277 |  |  |  |

Table S1 continued

| River name Year | 1992 | 1992 | 1992 | 1992 | 1992 | 1992 | 1992 | 1993 |
| --- | --- | --- | --- | --- | --- | --- | --- | --- |
| Broodstock origin^a,b^ | Probably MR HR | Probably MR HR | MR HR | MR HR | MR HR | MR HR |  | Probably MR |
| Run timing of broodstock | early and late | early and late | early and late | early and late | early and late | early and late |  | early and late |
| Young salmon were reared in semi-natural ponds | Most | None | All | All | All | All |  | None |
| Stage | 2+ smolts | Total, non-Morell | 1+ parr | 2+ parr | 2+ smolts | Total, Morell | Total, all rivers | 0+ parr |
| Tignish River |  |  |  |  |  |  |  |  |
| Mill River (Cains+Carruthers Brooks) | 3,657 | 3,826 |  |  |  |  | 3,826 |  |
| Trout River (Coleman) |  |  |  |  |  |  |  |  |
| Trout River (Tyne Valley) |  |  |  |  |  |  |  |  |
| Indian River |  |  |  |  |  |  |  |  |
| Hy Brook, Morrisons Pond |  |  |  |  |  |  |  |  |
| Trout River (Millvale) |  |  |  |  |  |  |  |  |
| Hunter River |  |  |  |  |  |  |  |  |
| Wheatley River |  |  |  |  |  |  |  |  |
| Black River |  |  |  |  |  |  |  |  |
| Bells Creek (Gurneys River) |  |  |  |  |  |  |  |  |
| Winter River |  |  |  |  |  |  |  |  |
| Morell River |  |  | 2,200 | 1,851 | 43,771 | 47,822 | 47,822 |  |
| Marie River |  |  |  |  |  |  |  |  |
| Midgell River |  |  |  |  |  |  |  | 20,000 |
| St. Peters River |  |  |  |  |  |  |  |  |
| Schooner Pond |  |  |  |  |  |  |  |  |
| McAskill Creek (Goose River) |  |  |  |  |  |  |  |  |
| Naufrage River |  |  |  |  |  |  |  |  |
| Cross Creek |  |  |  |  |  |  |  |  |
| North Lake Creek |  |  |  |  |  |  |  |  |
| Bakers River^e^ |  |  |  |  |  |  |  |  |
| Black Pond Creek |  |  |  |  |  |  |  |  |
| Souris River |  |  |  |  |  |  |  |  |
| Rollo Bay |  |  |  |  |  |  |  |  |
| Fortune River |  |  |  |  |  |  |  |  |
| Cardigan River |  |  |  |  |  |  |  |  |
| Brudenell River |  |  |  |  |  |  |  |  |
| Montague River |  |  |  |  |  |  |  |  |
| Valleyfield River | 12,000 | 54,508 |  |  |  |  | 54,508 | 14,467 |
| Sturgeon River |  |  |  |  |  |  |  |  |
| Murray River |  |  |  |  |  |  |  |  |
| Fox River |  |  |  |  |  |  |  |  |
| Belle River |  |  |  |  |  |  |  |  |
| Vernon River |  |  |  |  |  |  |  |  |
| Forbes Creek (southeast branch of Fullertons ) |  |  |  |  |  |  |  |  |
| Johnstons River |  |  |  |  |  |  |  |  |
| Glenfinnan River (Sherrys Creek) |  |  |  |  |  |  |  |  |
| Head of Hillsborough River |  |  |  |  |  |  |  |  |
| Hillsborough (East) River, unspecified location |  |  |  |  |  |  |  |  |
| North River |  |  |  |  |  |  |  |  |
| Clyde River |  |  |  |  |  |  |  |  |
| West River | 11,481 | 21,654 |  |  |  |  | 21,654 |  |
| Desable River |  |  |  |  |  |  |  |  |
| Westmoreland River |  |  |  |  |  |  |  |  |
| Tryon River |  |  |  |  |  |  |  |  |
| Dunk River |  |  |  |  |  |  |  |  |
| Wilmot River |  |  |  |  |  |  |  |  |
| Miminigash River |  |  |  |  |  |  |  |  |
| Skinners Pond |  |  |  |  |  |  |  |  |
| Nail Pond |  |  |  |  |  |  |  |  |
| Curtisdale River^f^ |  |  |  |  |  |  |  |  |
| Inspector River |  |  |  |  |  |  |  |  |
| Mores River^g^ |  |  |  |  |  |  |  |  |
| Trout Newbarton |  |  |  |  |  |  |  |  |
| Breakdown by river not reported |  |  |  |  |  |  |  |  |
| Total number released | 27,138 | 79,988 | 2,200 | 1,851 | 43,771 | 47,822 | 127,810 | 34,467 |
| Total number released, annual sums only |  |  |  |  |  |  | 127,810 |  |

Table S1 continued

| River name Year | 1993 | 1993 | 1993 | 1993 | 1993 | 1993 | 1994 | 1994 |
| --- | --- | --- | --- | --- | --- | --- | --- | --- |
| Broodstock origin^a,b^ | Probably MR | Probably MR | Probably MR | Probably MR | MR |  | Probably MR HR | Probably MR HR |
| Run timing of broodstock | early and late | early and late | early and late | early and late | early and late |  | early and late | early and late |
| Young salmon were reared in semi-natural ponds | Some | All | All | None | All |  | None | All |
| Stage | 1+ parr | 2+ parr | 2+ smolts | Total, non-Morell | 1+ smolts | Total, all rivers | 0+ parr | 2+ parr |
| Tignish River |  |  |  |  |  |  |  |  |
| Mill River (Cains+Carruthers Brooks) |  | 200 | 2,772 | 2,972 |  | 2,972 |  | 127 |
| Trout River (Coleman) |  |  |  |  |  |  |  |  |
| Trout River (Tyne Valley) |  |  |  |  |  |  |  |  |
| Indian River |  |  |  |  |  |  |  |  |
| Hy Brook, Morrisons Pond |  |  |  |  |  |  |  |  |
| Trout River (Millvale) |  |  |  |  |  |  |  |  |
| Hunter River |  |  |  |  |  |  |  |  |
| Wheatley River |  |  |  |  |  |  |  |  |
| Black River |  |  |  |  |  |  |  |  |
| Bells Creek (Gurneys River) |  |  |  |  |  |  |  |  |
| Winter River |  |  |  |  |  |  |  |  |
| Morell River |  |  |  |  | 19,379 | 19,379 |  |  |
| Marie River |  |  |  |  |  |  |  |  |
| Midgell River |  |  |  | 20,000 |  | 20,000 | 20,000 |  |
| St. Peters River |  |  |  |  |  |  |  |  |
| Schooner Pond |  |  |  |  |  |  |  |  |
| McAskill Creek (Goose River) |  |  |  |  |  |  |  |  |
| Naufrage River |  |  |  |  |  |  |  |  |
| Cross Creek |  |  |  |  |  |  |  |  |
| North Lake Creek |  |  |  |  |  |  |  |  |
| Bakers River^e^ |  |  |  |  |  |  |  |  |
| Black Pond Creek |  |  |  |  |  |  |  |  |
| Souris River |  |  |  |  |  |  |  |  |
| Rollo Bay |  |  |  |  |  |  |  |  |
| Fortune River |  |  |  |  |  |  |  |  |
| Cardigan River |  |  |  |  |  |  |  |  |
| Brudenell River |  |  |  |  |  |  |  |  |
| Montague River |  |  |  |  |  |  |  |  |
| Valleyfield River | 28,898 |  |  | 43,365 |  | 43,365 | 20,000 |  |
| Sturgeon River |  |  |  |  |  |  |  |  |
| Murray River |  |  |  |  |  |  |  |  |
| Fox River |  |  |  |  |  |  |  |  |
| Belle River |  |  |  |  |  |  |  |  |
| Vernon River |  |  |  |  |  |  |  |  |
| Forbes Creek (southeast branch of Fullertons ) |  |  |  |  |  |  |  |  |
| Johnstons River |  |  |  |  |  |  |  |  |
| Glenfinnan River (Sherrys Creek) |  |  |  |  |  |  |  |  |
| Head of Hillsborough River |  |  |  |  |  |  |  |  |
| Hillsborough (East) River, unspecified location |  |  |  |  |  |  |  |  |
| North River |  |  |  |  |  |  |  |  |
| Clyde River |  |  |  |  |  |  |  |  |
| West River |  |  |  |  |  |  |  | 209 |
| Desable River |  |  |  |  |  |  |  |  |
| Westmoreland River |  |  |  |  |  |  |  |  |
| Tryon River |  |  |  |  |  |  |  |  |
| Dunk River | 17,225 |  | 5,325 | 22,550 |  | 22,550 |  | 341 |
| Wilmot River |  |  |  |  |  |  |  |  |
| Miminigash River |  |  |  |  |  |  |  |  |
| Skinners Pond |  |  |  |  |  |  |  |  |
| Nail Pond |  |  |  |  |  |  |  |  |
| Curtisdale River^f^ |  |  |  |  |  |  |  |  |
| Inspector River |  |  |  |  |  |  |  |  |
| Mores River^g^ |  |  |  |  |  |  |  |  |
| Trout Newbarton |  |  |  |  |  |  |  |  |
| Breakdown by river not reported |  |  |  |  |  |  |  |  |
| Total number released | 46,123 | 200 | 8,097 | 88,887 | 19,379 | 108,266 | 40,000 | 677 |
| Total number released, annual sums only |  |  |  |  |  | 108,266 |  |  |

Table S1 continued

| River name Year | 1994 | 1994 | 1994 | 1994 | 1994 | 1994 | 1994 | 1995 |
| --- | --- | --- | --- | --- | --- | --- | --- | --- |
| Broodstock origin^a,b^ | Probably MR HR | Probably MR HR | Probably MR HR | MR HR | MR HR | MR HR |  | Probably MR HR |
| Run timing of broodstock | early and late | early and late | early and late | early and late | early and late | early and late |  | early and late |
| Young salmon were reared in semi-natural ponds | None | All | None | All | All | All |  | None |
| Stage | 1+ smolts | 2+ smolts | Total, non-Morell | 2+ parr | 2+ smolts | Total, Morell | Total, all rivers | 1+ parr |
| Tignish River |  |  |  |  |  |  |  |  |
| Mill River (Cains+Carruthers Brooks) |  | 2,584 | 2,711 |  |  |  | 2,711 |  |
| Trout River (Coleman) |  |  |  |  |  |  |  |  |
| Trout River (Tyne Valley) |  |  |  |  |  |  |  |  |
| Indian River |  |  |  |  |  |  |  |  |
| Hy Brook, Morrisons Pond |  |  |  |  |  |  |  |  |
| Trout River (Millvale) |  |  |  |  |  |  |  |  |
| Hunter River |  |  |  |  |  |  |  |  |
| Wheatley River |  |  |  |  |  |  |  |  |
| Black River |  |  |  |  |  |  |  |  |
| Bells Creek (Gurneys River) |  |  |  |  |  |  |  |  |
| Winter River |  |  |  |  |  |  |  |  |
| Morell River |  |  |  | 737 | 25,263 | 26,000 | 26,000 |  |
| Marie River |  |  |  |  |  |  |  |  |
| Midgell River |  |  | 20,000 |  |  |  | 20,000 | 9,367 |
| St. Peters River |  |  |  |  |  |  |  |  |
| Schooner Pond |  |  |  |  |  |  |  |  |
| McAskill Creek (Goose River) |  |  |  |  |  |  |  |  |
| Naufrage River |  |  |  |  |  |  |  |  |
| Cross Creek |  |  |  |  |  |  |  |  |
| North Lake Creek |  |  |  |  |  |  |  |  |
| Bakers River^e^ |  |  |  |  |  |  |  |  |
| Black Pond Creek |  |  |  |  |  |  |  |  |
| Souris River |  |  |  |  |  |  |  |  |
| Rollo Bay |  |  |  |  |  |  |  |  |
| Fortune River |  |  |  |  |  |  |  |  |
| Cardigan River |  |  |  |  |  |  |  |  |
| Brudenell River |  |  |  |  |  |  |  |  |
| Montague River |  |  |  |  |  |  |  |  |
| Valleyfield River | 5,896 | 1,980 | 27,876 |  |  |  | 27,876 | 11,585 |
| Sturgeon River |  |  |  |  |  |  |  |  |
| Murray River |  |  |  |  |  |  |  |  |
| Fox River |  |  |  |  |  |  |  |  |
| Belle River |  |  |  |  |  |  |  |  |
| Vernon River |  |  |  |  |  |  |  |  |
| Forbes Creek (southeast branch of Fullertons ) |  |  |  |  |  |  |  |  |
| Johnstons River |  |  |  |  |  |  |  |  |
| Glenfinnan River (Sherrys Creek) |  |  |  |  |  |  |  |  |
| Head of Hillsborough River |  |  |  |  |  |  |  |  |
| Hillsborough (East) River, unspecified location |  |  |  |  |  |  |  |  |
| North River |  |  |  |  |  |  |  |  |
| Clyde River |  |  |  |  |  |  |  |  |
| West River | 3,965 | 3,355 | 7,529 |  |  |  | 7,529 |  |
| Desable River |  |  |  |  |  |  |  |  |
| Westmoreland River |  |  |  |  |  |  |  |  |
| Tryon River |  |  |  |  |  |  |  |  |
| Dunk River |  | 7,259 | 7,600 |  |  |  | 7,600 |  |
| Wilmot River |  |  |  |  |  |  |  |  |
| Miminigash River |  |  |  |  |  |  |  |  |
| Skinners Pond |  |  |  |  |  |  |  |  |
| Nail Pond |  |  |  |  |  |  |  |  |
| Curtisdale River^f^ |  |  |  |  |  |  |  |  |
| Inspector River |  |  |  |  |  |  |  |  |
| Mores River^g^ |  |  |  |  |  |  |  |  |
| Trout Newbarton |  |  |  |  |  |  |  |  |
| Breakdown by river not reported |  |  |  |  |  |  |  |  |
| Total number released | 9,861 | 15,178 | 65,716 | 737 | 25,263 | 26,000 | 91,716 | 20,952 |
| Total number released, annual sums only |  |  |  |  |  |  | 91,716 |  |

Table S1 continued

| River name Year | 1995 | 1995 | 1995 | 1995 | 1995 | 1995 | 1995 | 1996 |
| --- | --- | --- | --- | --- | --- | --- | --- | --- |
| Broodstock origin^a,b^ | Probably MR HR | Probably MR HR | Probably MR HR | MR HR | MR HR | MR HR |  | Probably MR HR |
| Run timing of broodstock | early and late | early and late | early and late | early and late | early and late | early and late |  | early and late |
| Young salmon were reared in semi-natural ponds | All | All | None | All | All | All |  | None |
| Stage | 2+ parr | 2+ smolts | Total, non-Morell | 2+ parr | 2+ smolts | Total, Morell | Total, all rivers | 1+ parr |
| Tignish River |  |  |  |  |  |  |  |  |
| Mill River (Cains+Carruthers Brooks) | 364 | 3,923 | 4,287 |  |  |  | 4,287 |  |
| Trout River (Coleman) |  |  |  |  |  |  |  |  |
| Trout River (Tyne Valley) |  |  |  |  |  |  |  |  |
| Indian River |  |  |  |  |  |  |  |  |
| Hy Brook, Morrisons Pond |  |  |  |  |  |  |  |  |
| Trout River (Millvale) |  |  |  |  |  |  |  |  |
| Hunter River |  |  |  |  |  |  |  |  |
| Wheatley River |  |  |  |  |  |  |  |  |
| Black River |  |  |  |  |  |  |  |  |
| Bells Creek (Gurneys River) |  |  |  |  |  |  |  |  |
| Winter River |  |  |  |  |  |  |  |  |
| Morell River |  |  |  | 1,862 | 13,706 | 15,568 | 15,568 |  |
| Marie River |  |  |  |  |  |  |  |  |
| Midgell River |  |  | 9,367 |  |  |  | 9,367 | 8,564 |
| St. Peters River |  |  |  |  |  |  |  |  |
| Schooner Pond |  |  |  |  |  |  |  |  |
| McAskill Creek (Goose River) |  |  |  |  |  |  |  |  |
| Naufrage River |  |  |  |  |  |  |  |  |
| Cross Creek |  |  |  |  |  |  |  |  |
| North Lake Creek |  |  |  |  |  |  |  |  |
| Bakers River^e^ |  |  |  |  |  |  |  |  |
| Black Pond Creek |  |  |  |  |  |  |  |  |
| Souris River |  |  |  |  |  |  |  |  |
| Rollo Bay |  |  |  |  |  |  |  |  |
| Fortune River |  |  |  |  |  |  |  |  |
| Cardigan River |  |  |  |  |  |  |  |  |
| Brudenell River |  |  |  |  |  |  |  |  |
| Montague River |  |  |  |  |  |  |  |  |
| Valleyfield River | 3,937 | 11,580 | 27,102 |  |  |  | 27,102 |  |
| Sturgeon River |  |  |  |  |  |  |  |  |
| Murray River |  |  |  |  |  |  |  |  |
| Fox River |  |  |  |  |  |  |  |  |
| Belle River |  |  |  |  |  |  |  |  |
| Vernon River |  |  |  |  |  |  |  |  |
| Forbes Creek (southeast branch of Fullertons ) |  |  |  |  |  |  |  |  |
| Johnstons River |  |  |  |  |  |  |  |  |
| Glenfinnan River (Sherrys Creek) |  |  |  |  |  |  |  |  |
| Head of Hillsborough River |  |  |  |  |  |  |  |  |
| Hillsborough (East) River, unspecified location |  |  |  |  |  |  |  |  |
| North River |  |  |  |  |  |  |  |  |
| Clyde River |  |  |  |  |  |  |  |  |
| West River | 2,915 | 5,623 | 8,538 |  |  |  | 8,538 |  |
| Desable River |  |  |  |  |  |  |  |  |
| Westmoreland River |  |  |  |  |  |  |  |  |
| Tryon River |  |  |  |  |  |  |  |  |
| Dunk River | 280 | 5,179 | 5,459 |  |  |  | 5,459 |  |
| Wilmot River |  |  |  |  |  |  |  |  |
| Miminigash River |  |  |  |  |  |  |  |  |
| Skinners Pond |  |  |  |  |  |  |  |  |
| Nail Pond |  |  |  |  |  |  |  |  |
| Curtisdale River^f^ |  |  |  |  |  |  |  |  |
| Inspector River |  |  |  |  |  |  |  |  |
| Mores River^g^ |  |  |  |  |  |  |  |  |
| Trout Newbarton |  |  |  |  |  |  |  |  |
| Breakdown by river not reported |  |  |  |  |  |  |  |  |
| Total number released | 7,496 | 26,305 | 54,753 | 1,862 | 13,706 | 15,568 | 70,321 | 8,564 |
| Total number released, annual sums only |  |  |  |  |  |  | 70,321 |  |

Table S1 continued

| River name Year | 1996 | 1996 | 1996 | 1996 | 1996 | 1996 | 1996 | 1996 |
| --- | --- | --- | --- | --- | --- | --- | --- | --- |
| Broodstock origin^a,b^ | Probably MR HR | Probably MR HR | Probably MR HR | Probably MR HR | MR HR | MR HR | MR HR |  |
| Run timing of broodstock | early and late | early and late | early and late | early and late | early and late | early and late | early and late |  |
| Young salmon were reared in semi-natural ponds | All | None | All |  | All | All | All |  |
| Stage | 2+ parr | 1+ smolts | 2+ smolts | Total, non-Morell | 2+ parr | 2+ smolts | Total, Morell | Total, all rivers |
| Tignish River |  |  |  |  |  |  |  |  |
| Mill River (Cains+Carruthers Brooks) |  |  | 1,065 | 1,065 |  |  |  | 1,065 |
| Trout River (Coleman) |  |  |  |  |  |  |  |  |
| Trout River (Tyne Valley) |  |  |  |  |  |  |  |  |
| Indian River |  |  |  |  |  |  |  |  |
| Hy Brook, Morrisons Pond |  |  |  |  |  |  |  |  |
| Trout River (Millvale) |  |  |  |  |  |  |  |  |
| Hunter River |  |  |  |  |  |  |  |  |
| Wheatley River |  |  |  |  |  |  |  |  |
| Black River |  |  |  |  |  |  |  |  |
| Bells Creek (Gurneys River) |  |  |  |  |  |  |  |  |
| Winter River |  |  |  |  |  |  |  |  |
| Morell River |  |  |  |  | 5,573 | 41,019 | 46,592 | 46,592 |
| Marie River |  |  |  |  |  |  |  |  |
| Midgell River |  |  |  | 8,564 |  |  |  | 8,564 |
| St. Peters River |  |  |  |  |  |  |  |  |
| Schooner Pond |  |  |  |  |  |  |  |  |
| McAskill Creek (Goose River) |  |  |  |  |  |  |  |  |
| Naufrage River |  |  |  |  |  |  |  |  |
| Cross Creek |  |  |  |  |  |  |  |  |
| North Lake Creek |  |  |  |  |  |  |  |  |
| Bakers River^e^ |  |  |  |  |  |  |  |  |
| Black Pond Creek |  |  |  |  |  |  |  |  |
| Souris River |  |  |  |  |  |  |  |  |
| Rollo Bay |  |  |  |  |  |  |  |  |
| Fortune River |  |  |  |  |  |  |  |  |
| Cardigan River |  |  |  |  |  |  |  |  |
| Brudenell River |  |  |  |  |  |  |  |  |
| Montague River |  |  |  |  |  |  |  |  |
| Valleyfield River | 140 | 1,733 | 13,432 | 15,305 |  |  |  | 15,305 |
| Sturgeon River |  |  |  |  |  |  |  |  |
| Murray River |  |  |  |  |  |  |  |  |
| Fox River |  |  |  |  |  |  |  |  |
| Belle River |  |  |  |  |  |  |  |  |
| Vernon River |  |  |  |  |  |  |  |  |
| Forbes Creek (southeast branch of Fullertons ) |  |  |  |  |  |  |  |  |
| Johnstons River |  |  |  |  |  |  |  |  |
| Glenfinnan River (Sherrys Creek) |  |  |  |  |  |  |  |  |
| Head of Hillsborough River |  |  |  |  |  |  |  |  |
| Hillsborough (East) River, unspecified location |  |  |  |  |  |  |  |  |
| North River |  |  |  |  |  |  |  |  |
| Clyde River |  |  |  |  |  |  |  |  |
| West River | 212 |  | 6,759 | 6,971 |  |  |  | 6,971 |
| Desable River |  |  |  |  |  |  |  |  |
| Westmoreland River |  |  |  |  |  |  |  |  |
| Tryon River |  |  |  |  |  |  |  |  |
| Dunk River |  |  | 11,350 | 11,350 |  |  |  | 11,350 |
| Wilmot River |  |  |  |  |  |  |  |  |
| Miminigash River |  |  |  |  |  |  |  |  |
| Skinners Pond |  |  |  |  |  |  |  |  |
| Nail Pond |  |  |  |  |  |  |  |  |
| Curtisdale River^f^ |  |  |  |  |  |  |  |  |
| Inspector River |  |  |  |  |  |  |  |  |
| Mores River^g^ |  |  |  |  |  |  |  |  |
| Trout Newbarton |  |  |  |  |  |  |  |  |
| Breakdown by river not reported |  |  |  |  |  |  |  |  |
| Total number released | 352 | 1,733 | 32,606 | 43,255 | 5,573 | 41,019 | 46,592 | 89,847 |
| Total number released, annual sums only |  |  |  |  |  |  |  | 89,847 |

Table S1 continued

| River name Year | 1997 | 1997 | 1997 | 1997 | 1997 | 1997 | 1997 | 1997 |
| --- | --- | --- | --- | --- | --- | --- | --- | --- |
| Broodstock origin^a,b^ | Probably MR HR | Probably MR HR | Probably MR HR | Probably MR HR | MR HR | MR HR | MR HR |  |
| Run timing of broodstock | early and late | early and late | early and late | early and late | early and late | early and late | early and late |  |
| Young salmon were reared in semi-natural ponds | None | None | All |  | All | All | All |  |
| Stage | 1+ parr | 1+ smolts | 2+ smolts | Total, non-Morell | 2+ parr | 2+ smolts | Total, Morell | Total, all rivers |
| Tignish River |  |  |  |  |  |  |  |  |
| Mill River (Cains+Carruthers Brooks) |  |  |  |  |  |  |  |  |
| Trout River (Coleman) |  |  |  |  |  |  |  |  |
| Trout River (Tyne Valley) |  |  |  |  |  |  |  |  |
| Indian River |  |  |  |  |  |  |  |  |
| Hy Brook, Morrisons Pond |  |  |  |  |  |  |  |  |
| Trout River (Millvale) |  |  |  |  |  |  |  |  |
| Hunter River |  |  |  |  |  |  |  |  |
| Wheatley River |  |  |  |  |  |  |  |  |
| Black River |  |  |  |  |  |  |  |  |
| Bells Creek (Gurneys River) |  |  |  |  |  |  |  |  |
| Winter River |  |  |  |  |  |  |  |  |
| Morell River |  |  |  |  | 5,597 | 41,203 | 46,800 | 46,800 |
| Marie River |  |  |  |  |  |  |  |  |
| Midgell River | 4,900 |  |  | 4,900 |  |  |  | 4,900 |
| St. Peters River |  |  |  |  |  |  |  |  |
| Schooner Pond |  |  |  |  |  |  |  |  |
| McAskill Creek (Goose River) |  |  |  |  |  |  |  |  |
| Naufrage River |  |  |  |  |  |  |  |  |
| Cross Creek |  |  |  |  |  |  |  |  |
| North Lake Creek |  |  |  |  |  |  |  |  |
| Bakers River^e^ |  |  |  |  |  |  |  |  |
| Black Pond Creek |  |  |  |  |  |  |  |  |
| Souris River |  |  |  |  |  |  |  |  |
| Rollo Bay |  |  |  |  |  |  |  |  |
| Fortune River |  |  |  |  |  |  |  |  |
| Cardigan River |  |  |  |  |  |  |  |  |
| Brudenell River |  |  |  |  |  |  |  |  |
| Montague River |  |  |  |  |  |  |  |  |
| Valleyfield River |  | 3,044 | 8,527 | 11,571 |  |  |  | 11,571 |
| Sturgeon River |  |  |  |  |  |  |  |  |
| Murray River |  |  |  |  |  |  |  |  |
| Fox River |  |  |  |  |  |  |  |  |
| Belle River |  |  |  |  |  |  |  |  |
| Vernon River |  |  |  |  |  |  |  |  |
| Forbes Creek (southeast branch of Fullertons ) |  |  |  |  |  |  |  |  |
| Johnstons River |  |  |  |  |  |  |  |  |
| Glenfinnan River (Sherrys Creek) |  |  |  |  |  |  |  |  |
| Head of Hillsborough River |  |  |  |  |  |  |  |  |
| Hillsborough (East) River, unspecified location |  |  |  |  |  |  |  |  |
| North River |  |  |  |  |  |  |  |  |
| Clyde River |  |  |  |  |  |  |  |  |
| West River |  |  | 1,766 | 1,766 |  |  |  | 1,766 |
| Desable River |  |  |  |  |  |  |  |  |
| Westmoreland River |  |  |  |  |  |  |  |  |
| Tryon River |  |  |  |  |  |  |  |  |
| Dunk River |  |  |  |  |  |  |  |  |
| Wilmot River |  |  |  |  |  |  |  |  |
| Miminigash River |  |  |  |  |  |  |  |  |
| Skinners Pond |  |  |  |  |  |  |  |  |
| Nail Pond |  |  |  |  |  |  |  |  |
| Curtisdale River^f^ |  |  |  |  |  |  |  |  |
| Inspector River |  |  |  |  |  |  |  |  |
| Mores River^g^ |  |  |  |  |  |  |  |  |
| Trout Newbarton |  |  |  |  |  |  |  |  |
| Breakdown by river not reported |  |  |  |  |  |  |  |  |
| Total number released | 4,900 | 3,044 | 10,293 | 18,237 | 5,597 | 41,203 | 46,800 | 65,037 |
| Total number released, annual sums only |  |  |  |  |  |  |  | 65,037 |

Table S1 continued

| River name Year | 1998 | 1998 | 1998 | 1998 | 1998 | 1998 | 1998 | 1998 |
| --- | --- | --- | --- | --- | --- | --- | --- | --- |
| Broodstock origin^a,b^ | Probably MR HR | Probably MR HR | Probably MR HR | Probably MR HR | MR HR | MR HR | MR HR |  |
| Run timing of broodstock | early and late | early and late | early and late | early and late | early and late | early and late | early and late |  |
| Young salmon were reared in semi-natural ponds | All | All | Most |  | All | All | All |  |
| Stage | 1+ parr | 2+ parr | 2+ smolts | Total, non-Morell | 2+ parr | 2+ smolts | Total, Morell | Total, all rivers |
| Tignish River |  |  |  |  |  |  |  |  |
| Mill River (Cains+Carruthers Brooks) |  | 136 | 1,842 | 1,978 |  |  |  | 1,978 |
| Trout River (Coleman) |  | 1,830 | 15,691 | 17,521 |  |  |  | 17,521 |
| Trout River (Tyne Valley) |  |  |  |  |  |  |  |  |
| Indian River |  |  |  |  |  |  |  |  |
| Hy Brook, Morrisons Pond |  |  |  |  |  |  |  |  |
| Trout River (Millvale) |  |  |  |  |  |  |  |  |
| Hunter River |  |  |  |  |  |  |  |  |
| Wheatley River |  |  |  |  |  |  |  |  |
| Black River |  |  |  |  |  |  |  |  |
| Bells Creek (Gurneys River) |  |  |  |  |  |  |  |  |
| Winter River |  |  |  |  |  |  |  |  |
| Morell River |  |  |  |  | 5,453 | 40,138 | 45,591 | 45,591 |
| Marie River |  |  |  |  |  |  |  |  |
| Midgell River |  |  |  |  |  |  |  |  |
| St. Peters River |  |  |  |  |  |  |  |  |
| Schooner Pond |  |  |  |  |  |  |  |  |
| McAskill Creek (Goose River) |  |  |  |  |  |  |  |  |
| Naufrage River |  |  |  |  |  |  |  |  |
| Cross Creek |  |  |  |  |  |  |  |  |
| North Lake Creek |  |  |  |  |  |  |  |  |
| Bakers River^e^ |  |  |  |  |  |  |  |  |
| Black Pond Creek |  |  |  |  |  |  |  |  |
| Souris River |  |  |  |  |  |  |  |  |
| Rollo Bay |  |  |  |  |  |  |  |  |
| Fortune River |  |  |  |  |  |  |  |  |
| Cardigan River |  |  |  |  |  |  |  |  |
| Brudenell River |  |  |  |  |  |  |  |  |
| Montague River |  |  |  |  |  |  |  |  |
| Valleyfield River | 4,200 |  | 5,400 | 9,600 |  |  |  | 9,600 |
| Sturgeon River |  |  |  |  |  |  |  |  |
| Murray River |  |  |  |  |  |  |  |  |
| Fox River |  |  |  |  |  |  |  |  |
| Belle River |  |  |  |  |  |  |  |  |
| Vernon River |  |  |  |  |  |  |  |  |
| Forbes Creek (southeast branch of Fullertons ) |  |  |  |  |  |  |  |  |
| Johnstons River |  |  |  |  |  |  |  |  |
| Glenfinnan River (Sherrys Creek) |  |  |  |  |  |  |  |  |
| Head of Hillsborough River |  |  |  |  |  |  |  |  |
| Hillsborough (East) River, unspecified location |  |  |  |  |  |  |  |  |
| North River |  |  |  |  |  |  |  |  |
| Clyde River |  |  |  |  |  |  |  |  |
| West River |  |  | 10,206 | 10,206 |  |  |  | 10,206 |
| Desable River |  |  |  |  |  |  |  |  |
| Westmoreland River |  |  |  |  |  |  |  |  |
| Tryon River |  |  |  |  |  |  |  |  |
| Dunk River |  | 616 | 4,562 | 5,178 |  |  |  | 5,178 |
| Wilmot River |  |  |  |  |  |  |  |  |
| Miminigash River |  |  |  |  |  |  |  |  |
| Skinners Pond |  |  |  |  |  |  |  |  |
| Nail Pond |  |  |  |  |  |  |  |  |
| Curtisdale River^f^ |  |  |  |  |  |  |  |  |
| Inspector River |  |  |  |  |  |  |  |  |
| Mores River^g^ |  |  |  |  |  |  |  |  |
| Trout Newbarton |  |  |  |  |  |  |  |  |
| Breakdown by river not reported |  |  |  |  |  |  |  |  |
| Total number released | 4,200 | 2,582 | 37,701 | 44,483 | 5,453 | 40,138 | 45,591 | 90,074 |
| Total number released, annual sums only |  |  |  |  |  |  |  | 90,074 |

Table S1 continued

| River name Year | 1999 | 1999 | 1999 | 1999 | 1999 | 2000 | 2001 | 2002 |
| --- | --- | --- | --- | --- | --- | --- | --- | --- |
| Broodstock origin^a,b^ | Probably MR HR | Probably MR HR | Probably MR HR | MR HR |  |  |  |  |
| Run timing of broodstock | early and late | early and late | early and late | early and late |  |  |  |  |
| Young salmon were reared in semi-natural ponds | None | All |  | All |  |  |  |  |
| Stage | 1+ parr | 2+ smolts | Total, non-Morell | 2+ smolts | Total, all rivers |  |  | 2+ smolts |
| Tignish River |  |  |  |  |  |  |  |  |
| Mill River (Cains+Carruthers Brooks) |  |  |  |  |  |  |  | 2,904 |
| Trout River (Coleman) |  |  | 21,000 |  | 21,000 |  |  |  |
| Trout River (Tyne Valley) |  |  |  |  |  |  |  |  |
| Indian River |  |  |  |  |  |  |  |  |
| Hy Brook, Morrisons Pond |  |  |  |  |  |  |  |  |
| Trout River (Millvale) |  |  |  |  |  |  |  |  |
| Hunter River |  |  |  |  |  |  |  |  |
| Wheatley River |  |  |  |  |  |  |  |  |
| Black River |  |  |  |  |  |  |  |  |
| Bells Creek (Gurneys River) |  |  |  |  |  |  |  |  |
| Winter River |  |  |  |  |  |  |  |  |
| Morell River |  |  |  | 45,224 | 45,224 |  |  |  |
| Marie River |  |  |  |  |  |  |  |  |
| Midgell River |  |  |  |  |  |  |  |  |
| St. Peters River |  |  |  |  |  |  |  |  |
| Schooner Pond |  |  |  |  |  |  |  |  |
| McAskill Creek (Goose River) |  |  |  |  |  |  |  |  |
| Naufrage River |  |  |  |  |  |  |  |  |
| Cross Creek |  |  |  |  |  |  |  |  |
| North Lake Creek |  |  |  |  |  |  |  |  |
| Bakers River^e^ |  |  |  |  |  |  |  |  |
| Black Pond Creek |  |  |  |  |  |  |  |  |
| Souris River |  |  |  |  |  |  |  |  |
| Rollo Bay |  |  |  |  |  |  |  |  |
| Fortune River |  |  |  |  |  |  |  |  |
| Cardigan River |  |  |  |  |  |  |  |  |
| Brudenell River |  |  |  |  |  |  |  |  |
| Montague River |  |  |  |  |  |  |  |  |
| Valleyfield River | 3,500 | 3,200 | 6,700 |  | 6,700 |  |  |  |
| Sturgeon River |  |  |  |  |  |  |  |  |
| Murray River |  |  |  |  |  |  |  |  |
| Fox River |  |  |  |  |  |  |  |  |
| Belle River |  |  |  |  |  |  |  |  |
| Vernon River |  |  |  |  |  |  |  |  |
| Forbes Creek (southeast branch of Fullertons ) |  |  |  |  |  |  |  |  |
| Johnstons River |  |  |  |  |  |  |  |  |
| Glenfinnan River (Sherrys Creek) |  |  |  |  |  |  |  |  |
| Head of Hillsborough River |  |  |  |  |  |  |  |  |
| Hillsborough (East) River, unspecified location |  |  |  |  |  |  |  |  |
| North River |  |  |  |  |  |  |  |  |
| Clyde River |  |  |  |  |  |  |  |  |
| West River |  |  |  |  |  |  |  |  |
| Desable River |  |  |  |  |  |  |  |  |
| Westmoreland River |  |  |  |  |  |  |  |  |
| Tryon River |  |  |  |  |  |  |  |  |
| Dunk River |  |  |  |  |  |  |  |  |
| Wilmot River |  |  |  |  |  |  |  |  |
| Miminigash River |  |  |  |  |  |  |  |  |
| Skinners Pond |  |  |  |  |  |  |  |  |
| Nail Pond |  |  |  |  |  |  |  |  |
| Curtisdale River^f^ |  |  |  |  |  |  |  |  |
| Inspector River |  |  |  |  |  |  |  |  |
| Mores River^g^ |  |  |  |  |  |  |  |  |
| Trout Newbarton |  |  |  |  |  |  |  |  |
| Breakdown by river not reported |  |  |  |  |  |  |  |  |
| Total number released | 3,500 | 3,200 | 27,700 | 45,224 | 72,924 |  |  | 2,904 |
| Total number released, annual sums only |  |  |  |  | 72,924 |  |  |  |

Table S1 continued

| River name Year | 2002 | 2003 | 2004 | 2005 | 2006 | 2007 | 2008 | 2009 |
| --- | --- | --- | --- | --- | --- | --- | --- | --- |
| Broodstock origin^a,b^ |  |  | MR HR |  |  |  |  | MR |
| Run timing of broodstock |  |  | Mixed early and late |  |  |  |  |  |
| Young salmon were reared in semi-natural ponds |  |  |  |  |  |  |  |  |
| Stage | Total, non-Morell |  | 2+ smolts |  |  |  |  | Fall fingerling |
| Tignish River |  |  |  |  |  |  |  |  |
| Mill River (Cains+Carruthers Brooks) | 2,904 |  |  |  |  |  |  |  |
| Trout River (Coleman) | 5,827 |  |  |  |  |  |  |  |
| Trout River (Tyne Valley) |  |  |  |  |  |  |  |  |
| Indian River |  |  |  |  |  |  |  |  |
| Hy Brook, Morrisons Pond |  |  |  |  |  |  |  |  |
| Trout River (Millvale) |  |  |  |  |  |  |  |  |
| Hunter River |  |  |  |  |  |  |  |  |
| Wheatley River |  |  |  |  |  |  |  |  |
| Black River |  |  |  |  |  |  |  |  |
| Bells Creek (Gurneys River) |  |  |  |  |  |  |  |  |
| Winter River |  |  |  |  |  |  |  |  |
| Morell River |  |  | 40,800 |  |  |  |  | 10,500 |
| Marie River |  |  |  |  |  |  |  |  |
| Midgell River |  |  |  |  |  |  |  |  |
| St. Peters River |  |  |  |  |  |  |  |  |
| Schooner Pond |  |  |  |  |  |  |  |  |
| McAskill Creek (Goose River) |  |  |  |  |  |  |  |  |
| Naufrage River |  |  |  |  |  |  |  |  |
| Cross Creek |  |  |  |  |  |  |  |  |
| North Lake Creek |  |  |  |  |  |  |  |  |
| Bakers River^e^ |  |  |  |  |  |  |  |  |
| Black Pond Creek |  |  |  |  |  |  |  |  |
| Souris River |  |  |  |  |  |  |  |  |
| Rollo Bay |  |  |  |  |  |  |  |  |
| Fortune River |  |  |  |  |  |  |  |  |
| Cardigan River |  |  |  |  |  |  |  |  |
| Brudenell River |  |  |  |  |  |  |  |  |
| Montague River |  |  |  |  |  |  |  |  |
| Valleyfield River |  |  |  |  |  |  |  |  |
| Sturgeon River |  |  |  |  |  |  |  |  |
| Murray River |  |  |  |  |  |  |  |  |
| Fox River |  |  |  |  |  |  |  |  |
| Belle River |  |  |  |  |  |  |  |  |
| Vernon River |  |  |  |  |  |  |  |  |
| Forbes Creek (southeast branch of Fullertons ) |  |  |  |  |  |  |  |  |
| Johnstons River |  |  |  |  |  |  |  |  |
| Glenfinnan River (Sherrys Creek) |  |  |  |  |  |  |  |  |
| Head of Hillsborough River |  |  |  |  |  |  |  |  |
| Hillsborough (East) River, unspecified location |  |  |  |  |  |  |  |  |
| North River |  |  |  |  |  |  |  |  |
| Clyde River |  |  |  |  |  |  |  |  |
| West River |  |  |  |  |  |  |  |  |
| Desable River |  |  |  |  |  |  |  |  |
| Westmoreland River |  |  |  |  |  |  |  |  |
| Tryon River |  |  |  |  |  |  |  |  |
| Dunk River | 30,899 |  |  |  |  |  |  |  |
| Wilmot River |  |  |  |  |  |  |  |  |
| Miminigash River |  |  |  |  |  |  |  |  |
| Skinners Pond |  |  |  |  |  |  |  |  |
| Nail Pond |  |  |  |  |  |  |  |  |
| Curtisdale River^f^ |  |  |  |  |  |  |  |  |
| Inspector River |  |  |  |  |  |  |  |  |
| Mores River^g^ |  |  |  |  |  |  |  |  |
| Trout Newbarton |  |  |  |  |  |  |  |  |
| Breakdown by river not reported |  |  |  |  |  |  |  |  |
| Total number released | 39,630 |  | 40,800 |  |  |  |  | 10,500 |
| Total number released, annual sums only | 39,630 |  | 40,800 |  |  |  |  | 10,500 |

Table S1 continued

| River name Year | 2010 | 2011 | 2012 | 2013 | 2014 | 2015 | 2016 | 2017 |
| --- | --- | --- | --- | --- | --- | --- | --- | --- |
| Broodstock origin^a,b^ |  | MR | MR |  |  | MR | MR | MR |
| Run timing of broodstock |  |  |  |  |  |  |  |  |
| Young salmon were reared in semi-natural ponds |  |  |  |  |  |  |  |  |
| Stage |  | Fall fingerling | Fry |  |  | Fry | Feeding fry, spring release | Stage not reported |
| Tignish River |  |  |  |  |  |  |  |  |
| Mill River (Cains+Carruthers Brooks) |  |  |  |  |  |  |  |  |
| Trout River (Coleman) |  |  |  |  |  |  |  |  |
| Trout River (Tyne Valley) |  |  |  |  |  |  |  |  |
| Indian River |  |  |  |  |  |  |  |  |
| Hy Brook, Morrisons Pond |  |  |  |  |  |  |  |  |
| Trout River (Millvale) |  |  |  |  |  |  |  |  |
| Hunter River |  |  |  |  |  |  |  |  |
| Wheatley River |  |  |  |  |  |  |  |  |
| Black River |  |  |  |  |  |  |  |  |
| Bells Creek (Gurneys River) |  |  |  |  |  |  |  |  |
| Winter River |  |  |  |  |  |  |  |  |
| Morell River |  | 7,500 | 15,000 |  |  |  | 60,000 | 50,000 |
| Marie River |  |  |  |  |  |  |  |  |
| Midgell River |  |  |  |  |  |  |  |  |
| St. Peters River |  |  |  |  |  |  |  |  |
| Schooner Pond |  |  |  |  |  |  |  |  |
| McAskill Creek (Goose River) |  |  |  |  |  |  |  |  |
| Naufrage River |  |  |  |  |  |  |  |  |
| Cross Creek |  |  |  |  |  |  |  |  |
| North Lake Creek |  |  |  |  |  |  |  |  |
| Bakers River^e^ |  |  |  |  |  |  |  |  |
| Black Pond Creek |  |  |  |  |  |  |  |  |
| Souris River |  |  |  |  |  |  |  |  |
| Rollo Bay |  |  |  |  |  |  |  |  |
| Fortune River |  |  |  |  |  |  |  |  |
| Cardigan River |  |  |  |  |  |  |  |  |
| Brudenell River |  |  |  |  |  |  |  |  |
| Montague River |  |  |  |  |  |  |  |  |
| Valleyfield River |  |  |  |  |  |  |  |  |
| Sturgeon River |  |  |  |  |  |  |  |  |
| Murray River |  |  |  |  |  |  |  |  |
| Fox River |  |  |  |  |  |  |  |  |
| Belle River |  |  |  |  |  |  |  |  |
| Vernon River |  |  |  |  |  |  |  |  |
| Forbes Creek (southeast branch of Fullertons ) |  |  |  |  |  |  |  |  |
| Johnstons River |  |  |  |  |  |  |  |  |
| Glenfinnan River (Sherrys Creek) |  |  |  |  |  |  |  |  |
| Head of Hillsborough River |  |  |  |  |  |  |  |  |
| Hillsborough (East) River, unspecified location |  |  |  |  |  |  |  |  |
| North River |  |  |  |  |  |  |  |  |
| Clyde River |  |  |  |  |  |  |  |  |
| West River |  |  |  |  |  |  | 60,000 |  |
| Desable River |  |  |  |  |  |  |  |  |
| Westmoreland River |  |  |  |  |  |  |  |  |
| Tryon River |  |  |  |  |  |  |  |  |
| Dunk River |  |  |  |  |  |  |  |  |
| Wilmot River |  |  |  |  |  |  |  |  |
| Miminigash River |  |  |  |  |  |  |  |  |
| Skinners Pond |  |  |  |  |  |  |  |  |
| Nail Pond |  |  |  |  |  |  |  |  |
| Curtisdale River^f^ |  |  |  |  |  |  |  |  |
| Inspector River |  |  |  |  |  |  |  |  |
| Mores River^g^ |  |  |  |  |  |  |  |  |
| Trout Newbarton |  |  |  |  |  |  |  |  |
| Breakdown by river not reported |  |  |  |  | 55,000 | 80,000 |  |  |
| Total number released |  | 7,500 | 15,000 |  | 55,000 | 80,000 | 120,000 | 50,000 |
| Total number released, annual sums only |  | 7,500 | 15,000 |  | 55,000 | 80,000 | 120,000 | 50,000 |

Table S1 continued

| River name Year | 2018 | 2019 | 2020 | 2021 | 2022 | 2023 |
| --- | --- | --- | --- | --- | --- | --- |
| Broodstock origin^a,b^ | MR | MR | MR | MR | MR | MR |
| Run timing of broodstock |  |  |  |  |  |  |
| Young salmon were reared in semi-natural ponds |  |  |  |  |  |  |
| Stage | Stage not reported | 0+ | Unfed fry | Unfed fry | Unfed fry | Unfed fry |
| Tignish River |  |  |  |  |  |  |
| Mill River (Cains+Carruthers Brooks) |  |  |  |  |  |  |
| Trout River (Coleman) |  |  |  |  |  |  |
| Trout River (Tyne Valley) |  |  |  |  |  |  |
| Indian River |  |  |  |  |  |  |
| Hy Brook, Morrisons Pond |  |  |  |  |  |  |
| Trout River (Millvale) |  |  |  |  |  |  |
| Hunter River |  |  |  |  |  |  |
| Wheatley River |  |  |  |  |  |  |
| Black River |  |  |  |  |  |  |
| Bells Creek (Gurneys River) |  |  |  |  |  |  |
| Winter River |  |  |  |  |  |  |
| Morell River | 12,000 | 50,000 | 63,000 | 75,000 | 60,000 | 45,000 |
| Marie River |  |  |  |  |  |  |
| Midgell River |  |  |  |  |  |  |
| St. Peters River |  |  |  |  |  |  |
| Schooner Pond |  |  |  |  |  |  |
| McAskill Creek (Goose River) |  |  |  |  |  |  |
| Naufrage River |  |  |  |  |  |  |
| Cross Creek |  |  |  |  |  |  |
| North Lake Creek |  |  |  |  |  |  |
| Bakers River^e^ |  |  |  |  |  |  |
| Black Pond Creek |  |  |  |  |  |  |
| Souris River |  |  |  |  |  |  |
| Rollo Bay |  |  |  |  |  |  |
| Fortune River |  |  |  |  |  |  |
| Cardigan River |  |  |  |  |  |  |
| Brudenell River |  |  |  |  |  |  |
| Montague River |  |  |  |  |  |  |
| Valleyfield River |  |  |  |  |  |  |
| Sturgeon River |  |  |  |  |  |  |
| Murray River |  |  |  |  |  |  |
| Fox River |  |  |  |  |  |  |
| Belle River |  |  |  |  |  |  |
| Vernon River |  |  |  |  |  |  |
| Forbes Creek (southeast branch of Fullertons ) |  |  |  |  |  |  |
| Johnstons River |  |  |  |  |  |  |
| Glenfinnan River (Sherrys Creek) |  |  |  |  |  |  |
| Head of Hillsborough River |  |  |  |  |  |  |
| Hillsborough (East) River, unspecified location |  |  |  |  |  |  |
| North River |  |  |  |  |  |  |
| Clyde River |  |  |  |  |  |  |
| West River | 3,000 | 31,000 | 15,000 | 36,000 | 30,000 |  |
| Desable River |  |  |  |  |  |  |
| Westmoreland River |  |  |  |  |  |  |
| Tryon River |  |  |  |  |  |  |
| Dunk River |  |  |  |  |  |  |
| Wilmot River |  |  |  |  |  |  |
| Miminigash River |  |  |  |  |  |  |
| Skinners Pond |  |  |  |  |  |  |
| Nail Pond |  |  |  |  |  |  |
| Curtisdale River^f^ |  |  |  |  |  |  |
| Inspector River |  |  |  |  |  |  |
| Mores River^g^ |  |  |  |  |  |  |
| Trout Newbarton |  |  |  |  |  |  |
| Breakdown by river not reported |  |  |  |  |  |  |
| Total number released | 15,000 | 81,000 | 78,000 | 111,000 | 90,000 | 45,000 |
| Total number released, annual sums only | 15,000 | 81,000 | 78,000 | 111,000 | 90,000 | 45,000 |

Table S1 continued

| River name Year | 1880-1974 | | 1975-1987 | | 1988-2023 | | 1880-2023 | |
| --- | --- | --- | --- | --- | --- | --- | --- | --- |
|  |  |  |  |  |  |  |  |  |
|  | Number Stocked | Stocking Intensity 100 m^2^ | Number Stocked | Stocking Intensity 100 m^2^ | Number Stocked | Stocking Intensity 100 m^2^ | Number Stocked | Stocking Intensity 100 m^2^ |
|  |  |  |  |  |  |  |  |  |
|  |  |  |  |  |  |  |  |  |
| Tignish River | 149,180 | 2.69624 | 0 | 0.000 | 0 | 0.000 | 149,180 | 1.779 |
| Mill River (Cains+Carruthers Brooks) | 20,300 | 0.36653 | 8,489 | 1.120 | 31,375 | 1.495 | 60,164 | 0.717 |
| Trout River (Coleman) | 325,000 | 2.44008 | 0 | 0.000 | 44,348 | 0.879 | 369,348 | 1.829 |
| Trout River (Tyne Valley) | 117,000 | 1.94621 | 0 | 0.000 | 0 | 0.000 | 117,000 | 1.284 |
| Indian River | 340,000 | 11.42474 | 0 | 0.000 | 0 | 0.000 | 340,000 | 7.537 |
| Hy Brook, Morrisons Pond | 200 |  | 0 |  | 0 |  | 200 |  |
| Trout River (Millvale) | 30,000 | 0.45250 | 0 | 0.000 | 0 | 0.000 | 30,000 | 0.299 |
| Hunter River | 55,000 | 0.49798 | 0 | 0.000 | 0 | 0.000 | 55,000 | 0.329 |
| Wheatley River | 589,000 | 8.16718 | 0 | 0.000 | 0 | 0.000 | 589,000 | 5.388 |
| Black River | 204,000 | 7.86368 | 0 | 0.000 | 0 | 0.000 | 204,000 | 5.188 |
| Bells Creek (Gurneys River) | 160,400 | 4.46444 | 0 | 0.000 | 0 | 0.000 | 160,400 | 2.945 |
| Winter River | 2,817,069 | 32.54606 | 0 | 0.000 | 0 | 0.000 | 2,817,069 | 21.471 |
| Morell River | 11,921,428 | 52.90953 | 169,446 | 5.496 | 920,389 | 10.779 | 13,011,263 | 38.097 |
| Marie River | 124,000 | 3.39838 | 0 | 0.000 | 0 | 0.000 | 124,000 | 2.242 |
| Midgell River | 2,156,600 | 27.17635 | 0 | 0.000 | 62,831 | 2.089 | 2,219,431 | 18.451 |
| St. Peters River | 1,077,040 | 19.43555 | 0 | 0.000 | 0 | 0.000 | 1,077,040 | 12.822 |
| Schooner Pond | 333,600 | 13.81226 | 0 | 0.000 | 0 | 0.000 | 333,600 | 9.112 |
| McAskill Creek (Goose River) | 55,000 | 4.17222 | 0 | 0.000 | 0 | 0.000 | 55,000 | 2.753 |
| Naufrage River | 1,136,440 | 20.97342 | 0 | 0.000 | 0 | 0.000 | 1,136,440 | 13.837 |
| Cross Creek | 76,120 | 1.38167 | 0 | 0.000 | 0 | 0.000 | 76,120 | 0.912 |
| North Lake Creek | 139,620 | 2.35166 | 0 | 0.000 | 0 | 0.000 | 139,620 | 1.551 |
| Bakers River^e^ | 252,000 |  | 0 |  | 0 |  | 252,000 |  |
| Black Pond Creek | 68,000 | 3.81734 | 0 | 0.000 | 0 | 0.000 | 68,000 | 2.518 |
| Souris River | 62,800 | 0.95009 | 0 | 0.000 | 0 | 0.000 | 62,800 | 0.627 |
| Rollo Bay | 30,000 | 1.12602 | 0 | 0.000 | 0 | 0.000 | 30,000 | 0.743 |
| Fortune River | 420,600 | 4.48785 | 0 | 0.000 | 0 | 0.000 | 420,600 | 2.961 |
| Cardigan River | 445,847 | 8.03464 | 0 | 0.000 | 0 | 0.000 | 445,847 | 5.301 |
| Brudenell River | 70,000 | 1.01803 | 0 | 0.000 | 0 | 0.000 | 70,000 | 0.672 |
| Montague River | 257,100 | 2.70949 | 0 | 0.000 | 0 | 0.000 | 257,100 | 1.788 |
| Valleyfield River | 0 | 0.00000 | 0 | 0.000 | 355,540 | 7.746 | 355,540 | 1.936 |
| Sturgeon River | 158,800 | 2.11409 | 0 | 0.000 | 0 | 0.000 | 158,800 | 1.395 |
| Murray River | 382,000 | 4.32811 | 0 | 0.000 | 0 | 0.000 | 382,000 | 2.855 |
| Fox River | 25,000 | 1.88790 | 0 | 0.000 | 0 | 0.000 | 25,000 | 1.245 |
| Belle River | 425,000 | 9.51399 | 0 | 0.000 | 0 | 0.000 | 425,000 | 6.277 |
| Vernon River | 75,000 | 0.87200 | 0 | 0.000 | 0 | 0.000 | 75,000 | 0.575 |
| Forbes Creek (southeast branch of Fullertons ) | 62,000 |  | 0 |  | 0 |  | 62,000 |  |
| Johnstons River | 194,120 | 3.97383 | 0 | 0.000 | 0 | 0.000 | 194,120 | 2.622 |
| Glenfinnan River (Sherrys Creek) | 148,500 | 3.58909 | 0 | 0.000 | 0 | 0.000 | 148,500 | 2.368 |
| Head of Hillsborough River | 592,220 | 8.96807 | 0 | 0.000 | 0 | 0.000 | 592,220 | 5.916 |
| Hillsborough (East) River, unspecified location | 70,000 |  | 0 |  | 0 |  | 70,000 |  |
| North River | 993,500 | 8.06617 | 0 | 0.000 | 0 | 0.000 | 993,500 | 5.321 |
| Clyde River | 144,000 | 2.77875 | 0 | 0.000 | 0 | 0.000 | 144,000 | 1.833 |
| West River | 310,100 | 1.76922 | 0 | 0.000 | 285,128 | 4.293 | 595,228 | 2.240 |
| Desable River | 30,000 | 0.55164 | 0 | 0.000 | 0 | 0.000 | 30,000 | 0.364 |
| Westmoreland River | 30,000 | 0.55892 | 0 | 0.000 | 0 | 0.000 | 30,000 | 0.369 |
| Tryon River | 10,000 | 0.14270 | 0 | 0.000 | 0 | 0.000 | 10,000 | 0.094 |
| Dunk River | 2,701,200 | 14.72653 | 0 | 0.000 | 85,053 | 1.224 | 2,786,253 | 10.021 |
| Wilmot River | 105,000 | 1.01236 | 0 | 0.000 | 0 | 0.000 | 105,000 | 0.668 |
| Miminigash River | 196,000 | 5.90497 | 0 | 0.000 | 0 | 0.000 | 196,000 | 3.896 |
| Skinners Pond | 40,800 | 3.72362 | 0 | 0.000 | 0 | 0.000 | 40,800 | 2.457 |
| Nail Pond | 60,800 | 2.97335 | 0 | 0.000 | 0 | 0.000 | 60,800 | 1.962 |
| Curtisdale River^f^ | 355 |  | 0 |  | 0 |  | 355 |  |
| Inspector River | 10,000 |  | 0 |  | 0 |  | 10,000 |  |
| Mores River^g^ | 21,000 |  | 0 |  | 0 |  | 21,000 |  |
| Trout Newbarton | x |  | 0 |  |  |  | x |  |
| Breakdown by river not reported | 4,770,000 |  | 0 |  | 135,000 |  | 4,905,000 |  |
| Total number released | 34,988,739 |  |  |  |  |  |  |  |
| Total number released, annual sums only | 34,988,739 | 10.85975 | 177,935 | 0.404 | 1,919,664 | 1.572 | 37,086,338 | 7.594 |

Table S1 footer:

| ^a^DR: Dunk River; RP-NS: River Phillip Nova Scotia; WR: West River; SJ-NB: Saint John River New Brunswick; MR-NB: Miramichi River New Brunswick; MR: Morell River; |
| --- |
| NWMR-NB: Northwest Miramichi River New Brunswick; RR-NB: Restigouche River New Brunswick; HR: fish hatched in hatcheries that were |
| released and later recaptured for use as broodstock. |
| ^b^This footnote explains why some broodstock origins are shown as "probable." |
| The government of Canada operated salmonid hatcheries at Kellys Pond PEI in what is now Stratford from 1905 to the late 1950s, and at |
| Cardigan PEI beginning in 1937 (Cairns et al. 2010). Stocking records specify broodstock origin for most years up to 1916, but origin is generally not reported in the following decades. |
| However, other historical sources contain multiple mentions of broodstock collecting in the Morell River and no mention of collecting elsewhere |
| (Cairns et al. 2010). Broodstock collection in the Morell appears to have ended in 1950 (Cairns et al. 2010). |
| Broodstock origin is therefore listed as probably Morell for the years 1925-1951, except for those years in which the diaries specifically state the origin. |
| Beginning in the late 1970s, a concerted effort was launched to restore Atlantic salmon populations in larger PEI rivers, especially the Morell, but also the |
| Mill, Valleyfield, West, and Dunk (Davidson and Angus 1994). This program had the specific aim to build early salmon runs to increase angling opportunities. |
| This was done by intensively stocking salmon of early-run New Brunswick lineage in the Morell River, and then using the Morell as a broodstock source after early runs were established. |
| Stocking records for the 1970s to the 1990s give broodstock origins of salmon released into the Morell, but origins are not specified for releases into other rivers. |
| Because the stocking program operated in an integrated fashion, it is likely that broodstock origin in a given year was the same across all rivers. |
| Broodstock origins of salmon released in the Morell are therefore listed as the probable broodstock origins of salmon released into other rivers in the same year. |
| ^c^Watershed areas and stream areas are from Cairns and MacFarlane 2015 where available. Otherwise, watershed areas are from a PEI watershed map and stream areas are from |
| a linear regresssion of stream areas and watershed areas from five large PEI streams. The equation is stream area (in m^2^) = 1.309.08 x watershed area (in km^2^) (Cairns et al. 2010).. |
| ^d^x means that the river received stocked Atlantic salmon, but the number released was not recorded. |
| ^e^identified by H. Arthur Smith, PEI Fish and Wildlife Division, retired, as a tributary of the estuary of North Lake Creek. The Cummins (1928) atlas shows Bakers as landowners in the area. |
| ^f^This might be a branch of the North River. The Meacham (1880) atlas shows a Curtis Creek in this area and the Cummins (1928) atlas shows Curtis as landowners in the area. |
| ^g^This might be the branch of the Sturgeon River where Moores Pond is located. |

Table S2: Standardization rules for comparison of microsatellite data between Moore et al. (2014) and the present study

| **Locus** | **Grove study to Moore et al. (2014)** |
| --- | --- |
| **SSsp2210** | No Change |
| **SSsp2215** | Add 1bp |
| **Ssa197** | Subtract 5bp |
| **Ssa85** | Subtract 3bp |
| **SsaD486** | Add 4bp |
| **SsosI417** | Subtract 2bp |


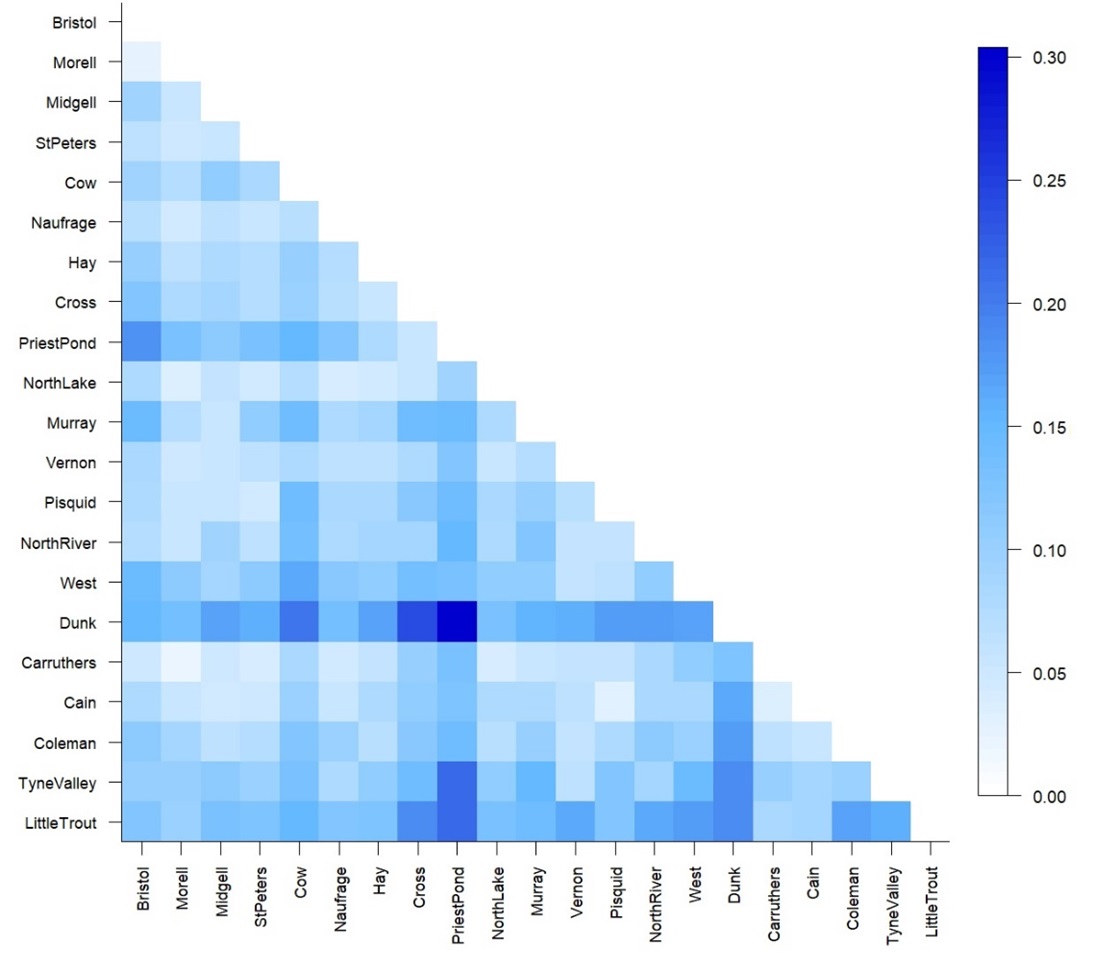


Figure S1. Heat map of pairwise FST values for 21 PEI Atlantic salmon populations. Darker cells indicate larger FST estimates thus greater genetic divergence, whereas lighter or white cells indicate lower estimates.


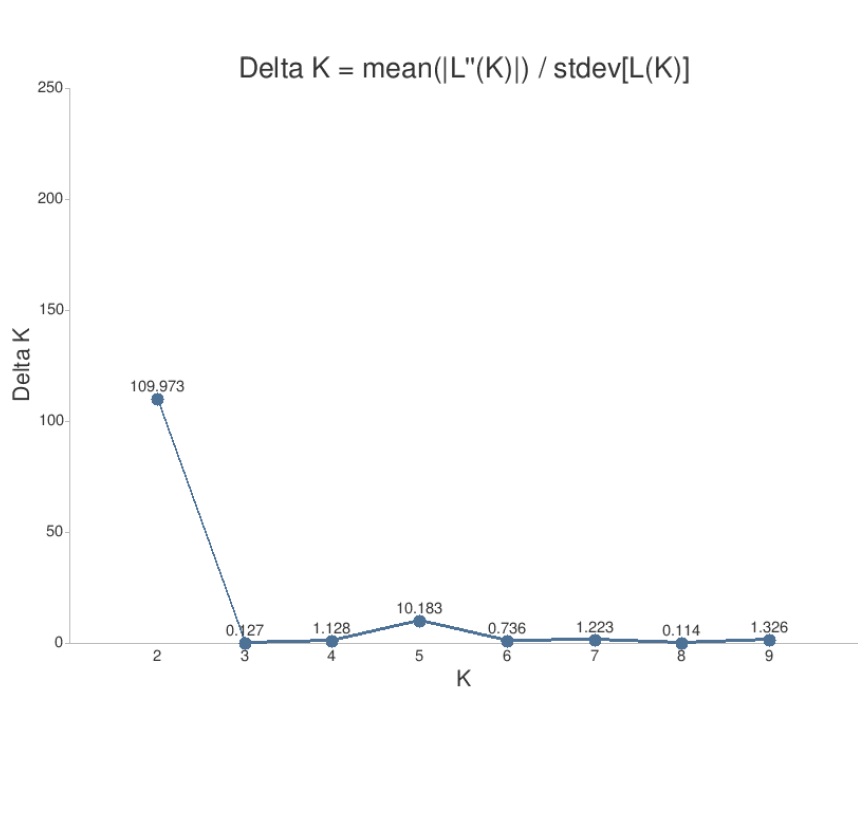


Figure S2. Delta K to determine appropriate K values from STRUCTURE data
